# Jusvinza, an anti-inflammatory drug derived from the human heat-shock protein 60, for critically ill COVID-19 patients. An observational study

**DOI:** 10.1371/journal.pone.0281111

**Published:** 2023-02-02

**Authors:** Rafael Venegas-Rodríguez, Anabel Serrano-Díaz, Ruben Peña-Ruiz, Raul Santana-Sánchez, Mabel Hernández-Cedeño, Aliusha Rittoles Navarro, Inti Grecesqui-Cruz, Liam Pérez-Aguilera, Anadys Segura-Fernández, Leticia Rosario-Cruz, Gilliam Martínez-Donato, Gerardo Guillén-Nieto, Maria del Carmen Domínguez- Horta

**Affiliations:** 1 Luis Díaz Soto Hospital, Habana del Este, Havana, Cuba; 2 Biomedical Research Department, Center for Genetic Engineering and Biotechnology, Havana, Cuba; Aminu Kano Teaching Hospital, NIGERIA

## Abstract

This paper presents the results of an observational and retrospective study on the therapeutic effects of Jusvinza, an immunomodulatory peptide with anti-inflammatory properties for critically ill COVID-19 patients. This peptide induces regulatory mechanisms on the immune response in experimental systems and in patients with Rheumatoid Arthritis. Exploratory research in COVID-19 patients revealed that Jusvinza promotes clinical and radiological improvement. The aim of this study is to describe the clinical outcome and variations of several inflammatory biomarkers in a cohort of critically ill COVID-19 patients, divided into two groups during the observational research: one group received Jusvinza and the other did not. Research physicians extracted the patients´ data from their hospital’s clinical records. The study analyzed 345 medical records, and 249 records from critically ill patients were included. The data covered the demographic characteristics, vital signs, ventilatory parameters and inflammatory biomarkers. Survival outcome was significantly higher in the group receiving Jusvinza (90.4%) compared to the group without Jusvinza (39.5%). Furthermore, in patients treated with Jusvinza there was a significant improvement in ventilatory parameters and a reduction in inflammation and coagulation biomarkers. Our findings show that Jusvinza could control the extent of inflammation in COVID-19 patients. This study indicates that Jusvinza is a helpful drug for the treatment of diseases characterized by hyperinflammation.

## Introduction

COVID-19 was first described by the end of 2019 in the Wuhan province of China [1]. The rapid worldwide spread of this disease led ‎World Health Organization (WHO) to declare it as a pandemic in March 2020. As of November 3, 2022, more than 600 million people have been infected with severe acute respiratory syndrome coronavirus virus 2 (SARS-CoV-2), and total deaths have exceeded 6,6million [2].

The clinical spectrum of COVID-19 is very wide and complex [3]. Patients clinical features may range from asymptomatic to those showing a rapid progression toward an acute respiratory distress syndrome (ARDS) and death [4]. Patients progressing to severe stages present an exacerbated inflammatory response, evidenced by the increase in the serum concentration of inflammation biomarkers [5].

On the other hand, several studies have reported a link between SARS-CoV-2 infection and the onset of a procoagulant condition, which increases the risk of arterial and venous thrombotic complications, with a fatal outcome [6–8]. The excessive activation of the innate immune system, caused by this virus, induces a significant increase in the concentration of several mediators of inflammation, which contribute to a consequent damage in the microvascular system and the activation of the coagulation process [9].

Under these conditions, the treatment of hyperinflammation is crucial [10]. Approved treatments for autoimmune and inflammatory diseases are normally used, however, these drugs are immunosuppressive and they can lead to the deterioration of the general condition of the COVID-19 patient [11, 12]. In Cuba, the drug called Jusvinza is used in COVID-19 patients with signs of hyperinflammation. This drug received an Emergency Use Authorization, emitted by the Cuban Regulatory Authority (CECMED) [13].

The peptide Jusvinza, initially named CIGB-814, was developed for the treatment of autoimmune diseases, specifically for rheumatoid arthritis (RA). The molecule induces regulatory mechanisms of immune response in experimental systems and in patients with RA [14–20]. The name of this peptide was changed to CIGB-258 during the research period with COVID-19 patients. Exploratory studies in COVID-19 patients revealed that Jusvinza promotes clinical and radiological improvement linked to a decrease in systemic inflammation biomarkers [21].

The inclusion of Jusvinza in the Cuban guideline approved by the Ministry of Public Health for COVID-19 took place on April 27th, 2020. The use of this drug has contributed to decrease the fatality rate in Cuba. In April 2020, the fatality rate in Cuba was 4.16. The monthly case fatality rate decreased to 1.2% in June [22] and in March 2021 it was 0.46 [23].

The objective of this study is to describe the clinical outcome and variations of several inflammation biomarkers in a cohort of critically ill COVID-19 patients, divided into two groups during the observational research: one group exposed to Jusvinza and the other did not.

## Methods

### Study design and cohort selection

This work was a retrospective and observational (non-interventional) study. The data were obtained from the patients´ clinical records (CR). This study was conducted at the Dr Luis Diaz Soto Hospital in Havana, Cuba. The patients were included in the data base according to the following criteria:

1. Positive SARS-CoV-2 diagnosis by reverse-transcriptase polymerase chain reaction
2. Classified as critically ill patients according to the Cuban national protocol approved by the Ministry of Public Health for COVID-19 [24]. The criteria for this classification are: (a) acute respiratory distress syndrome (ARDS) evidenced by the ratio of the arterial oxygen partial pressure to the fraction of inspired oxygen (P/F) ≤ 300 mm Hg [25]; (b) Sequential Organ Failure Assessment (SOFA) >2; (c) Bilateral multilobar interstitial pattern > 50% in chest x-rays.
3. None of the patients died during the first 48 hours at the ICU
4. Patients not included in any clinical trial
5. Patients hospitalized within the time frame from March 21, 2021 until July 29, 2021

The patients were excluded in the data base according to the following criteria:

1. Patients with SAR-CoV-2 infection not confirmed
2. Pregnant women
3. Patients who died within 48 hours of care in ICU
4. Patients included in other clinical investigation

**Fig 1:**
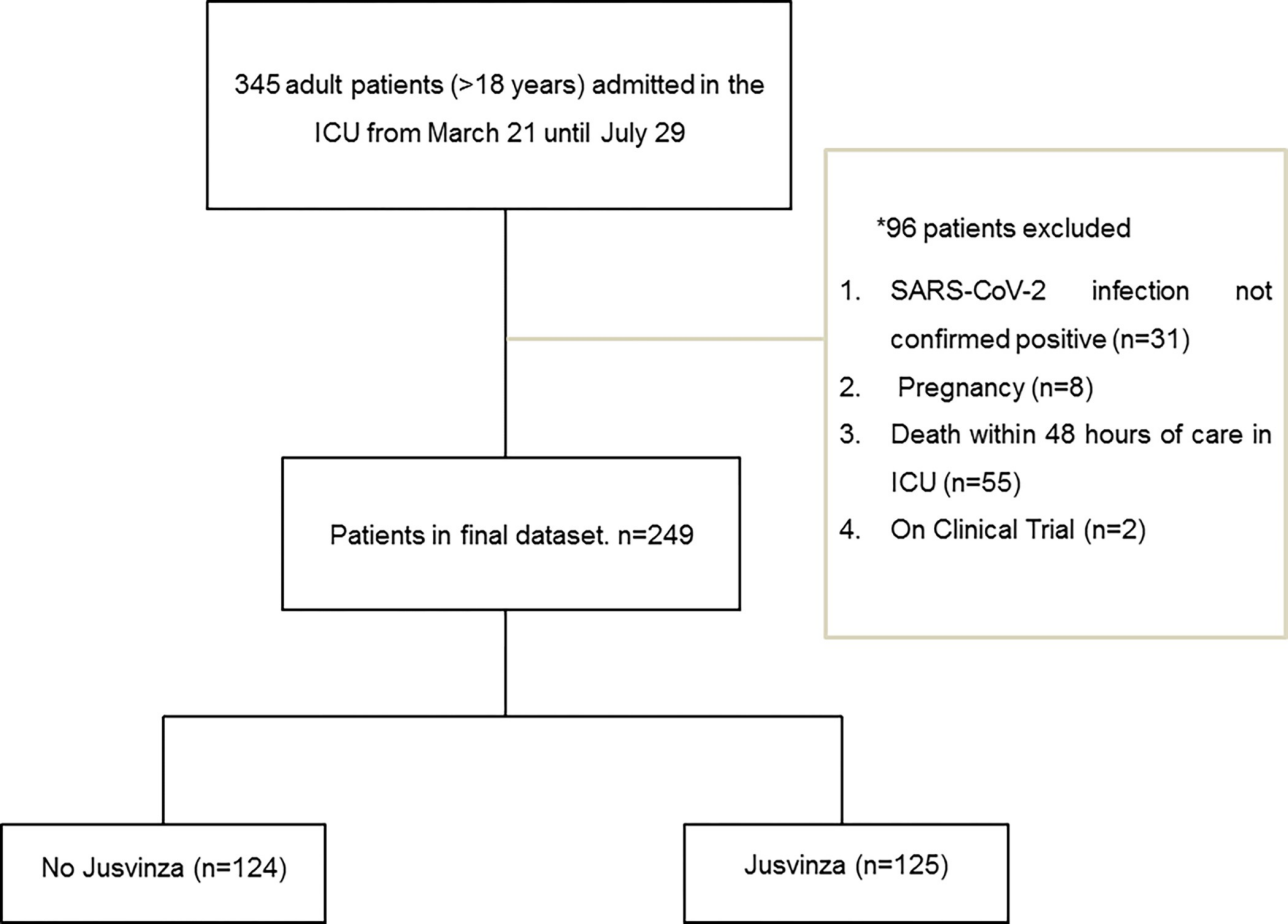
Cohort selection flow diagram. Flow Diagram of patient sampling strategy of cared at ICU in “Dr Luis Diaz Soto Hospital”.*96 patients were excluded from this study, because they did not meet the inclusion criteria. Follow-up occurred until the research cut-off date of August 30.

This study analyzed 345 medical records and included 249 critically ill patients (Fig 1).

### Ethical consideration

This study was conducted according to the Helsinki Declaration for research in humans [26] and the International Conference on Harmonization [27] guidelines. It was approved by the hospital´s Ethics and Scientific Committee and registered as RPCEC00000394 at the Cuban Clinical Trial Registry (https://rpcec.sld.cu/trials/RPCEC00000394-En).

### Data sources and variables assessed

Research physicians extracted the data stored in the CR of each patient. The data were anonymously recorded to ensure confidentiality. The information included demographic characteristics, vital signs (mean arterial pressure, heart rate and respiratory rate), laboratory test results and ventilatory parameters.

Laboratory tests obtained from the CR were: Erythrocyte Sedimentation Rate (ESR), white blood cell count (WBC), the neutrophil-to-lymphocyte ratio (NLR) in peripheral blood, and serum values of lactate dehydrogenase (LDH), dimer D (DD), ferritin, fibrinogen, C-reactive protein (CRP) and interleukin (IL)-6.

Laboratory test values were obtained for all patients included in this study, except for IL-6. IL-6 values from CR were acquired from March 21 until May 23. IL-6 values included 13 patients exposed to Jusvinza and eight patients not treated with Jusvinza.

The ventilatory parameters taken from the CR were: partial pressure of arterial blood oxygen (PO2), the ratio of arterial blood oxygen partial pressure to the fraction of inspired oxygen (P/F) and oxygen saturation (SO_2_). History of hypertension, diabetes mellitus, chronic lung disease or asthma, coronary artery disease and cerebrovascular disease were extracted from CR. When not listed in the CR, comorbidities were recorded as absent.

Data on safety were collected from the CR according to Regulation 45/2007 from the Cuban Regulatory Authority: “Requirements for reporting adverse events in ongoing clinical trials, based on WHO regulations.” This regulation conforms to the “National Cancer Institute Common Toxicity Criteria Adverse Events version 3.0” (National Cancer Institute, Frederick, MD, USA). The follow-up of patients took place until the research cut-off date of August 30^th^.

Information collected from the CR showed that all patients received the standard therapy established in the Cuban guideline approved by the Ministry of Public Health for COVID-19 [24], which included low-molecular-weight heparin, steroids and antibiotics.

The management of ARDS described in the CR included a staggered strategy of different oxygenation methods, according to the guideline for COVID-19 from the WHO [28].

The patients received high-flow nasal oxygen (HFNO) using nasal cannula [29] or non-invasive ventilation with the continuous positive airway pressure (CPAP) variant, according to the Rox [30] index or the HACOR scale [31], respectively. In some cases, these oxygenation therapies were combined with awake prone positioning. The patients that did not improve after short trials (about 1 hour) were subjected to endotracheal intubation. Patients who arrived at the ICU with severe ARDS (PaO2/FiO2 < 150) were managed with Invasive mechanical ventilation in prone ventilation for 12–16 hours per day [32].

### Jusvinza exposure

For the therapeutic effect of Jusvinza, the patients were divided into two groups– 1) treated with Jusvinza, –2) not treated with Jusvinza (control group).

The treatment with Jusvinza was defined as appointments for drug administration in the CR. If no data on the administration of Jusvinza appeared in the CR, it was considered that these patients did not receive the drug.

The Jusvinza administration algorithm was the following:

Patients were treated with 2 mg of Jusvinza every eight hours by intravenous route. According to CR, the patients were treated with Jusvinza until their serious clinical and radiological conditions were resolved, evidenced by:

- Patient not required oxygen therapy in the last 48 hours;
- Patient had ≥ 50% improvement in inflammatory and coagulation biomarkers;
- Patient had improvement on chest x-ray, with >50% resolution of initial lesions.

The treatment with Jusvinza was depended exclusively on the availability of this drug.

### Endpoints

The primary endpoint was death during the follow-up through August 30, 2021. Mortality was extracted from CR according a medical note announcing that the patient was deceased and the time of death. The secondary endpoints were the effects of Jusvinza on vital signs, ventilation parameters, inflammation and coagulation biomarkers and the IL-6 concentration in the serum.

### Statistical analysis

Data were analyzed using GraphPad Prism version 9.04 (GraphPad Software, San Diego California, USA). Categorical variables (Table 1) were expressed as the numbers and proportions (%), and compared using a Fisher’s exact test.

**Table 1:** Demographic characteristics, comorbidities and clinical outcomes of patients with COVID-19.

Characteristics	All patients(n = 249)	No Jusvinza(n = 124, 49.8%)	Jusvinza(n = 125, 50.2%)	*P value* ^*a*^
Age, media (range)	70 (59–89)	71 (59–89)	69 (65–81)	0.8499
Sex (Female/male)	131/118	66/58	65/60	0.8992
Race (white/mixed)	224/25	113/11	111/14	0.6740
**Comorbidities**				
Arterial hypertension	187 (75.1%)	89 (71.8%)	98 (78.4%)	0.2437
Ischemic heart	31 (12.4%)	13 (10.5%)	18 (14.4%)	0.4432
Diabetes mellitus	76 (30.5%)	41 (33.1%)	35 (28.0%)	0.4112
Chronic obstructive pulmonary disease	11 (4.4%)	5 (4.0%)	6 (4.8%)	>0.9999
Bronchial asthma	24 (9.6%)	12 (9.7%)	12 (9.6%)	>0.9999
Obesity^**b**^	100 (40.2%)	52 (41.9%)	48 (38.4%)	0.6063
≥ 2 comorbidities	147 (59.0%)	79 (63.7%)	68 (54.4%)	0.3769
Any comorbidities	2 (0.8%)	2 (1.6%)	-	0.2470
**Clinical outcomes**				
Total days on ICU	15.1 ± 9.8	19.3 ± 5.6	11 ± 2.8	**<0.0001**
Alive, discharged from hospital	162 (65.1%)	49 (39.5%)	113 (90.4%)	**<0.0001**
Dead	87 (34.9%)	75 (60.5%)	12 (9.6%)	**<0.0001**

P values for comparisons between Jusvinza and No Jusvinza treated patients. P < 0.05 was considered statistically significant. Age was analyzed with a **Mann Whitney**. The other variables were analyzed using a **Fisher’s exact test**

**b** Obesity was defined as a BMI of >30 kg/m^2^

Age and total days on ICU were analysis with a Mann-Whitney test. Survival time between the group patients exposure to Jusvinza and the group patients did not receive Jusvinza were compared using a Log-rank (Mantel-Cox) test in Kaplan-Meier curve.

Data on vital signs, ventilation parameters, NLR, LDH, DD, ferritin, fibrinogen, CRP, ESR and IL-6 were examined for normality and equal variance with Kolmogorov-Smirnov and Bartlett’s tests, respectively. Vital signs and ventilation parameters were analyzed using Friedman`test and Dunns`s correction test for multiple comparisons. NLR, LDH, DD, ferritin, fibrinogen, CRP and ESR was analyzed using Kruskal-Wallis test and Dunn’s correction test for multiple comparisons. IL-6 was analyzed using Wilcoxon matched-pairs signed rank test.The values were taken from the patients for comparisons during the first week at the ICU. Time zero (T0) corresponded to the values at the first blood test, when patients were admitted to the ICU. P values < 0.05 were considered statistically significant.

## Results

### Characteristics of the cohort

From 345 COVID-19 patients who were admitted to the ICU between March 21 and July 29, 2021, 96 patients were excluded from the study, because they did not meet the inclusion criteria. We included in this study 249 COVID-19 patients in critical conditions (Fig 1). Demographic characteristics and comorbidities of patients are summarized in Table 1.

The median age of patients was 70 years old. There were 247 patients with one or more comorbidities, including arterial hypertension, cardiovascular disease, diabetes mellitus, chronic obstructive pulmonary disease, bronchial asthma and obesity. More than two comorbidities were present in 59% of the patients; only two patients did not have comorbidities.

### Characteristics of the Jusvinza and non-Jusvinza groups. Primary endpoint

There were no significant differences in terms of age, sex, race and comorbidities between the groups treated or not treated with Jusvinza (Table 1).The average time at the ICU for the group treated with Jusvinza was 11 days, while for non-Jusvinza group was 19 days. The primary endpoint regarding death occurred in 12 patients (9. 6%) receiving Jusvinza and 75 patients (60.5%) who did not. Survival outcome was significantly higher in the group that received Jusvinza compared to the group without Jusvinza (Table 1).

**Fig 2:**
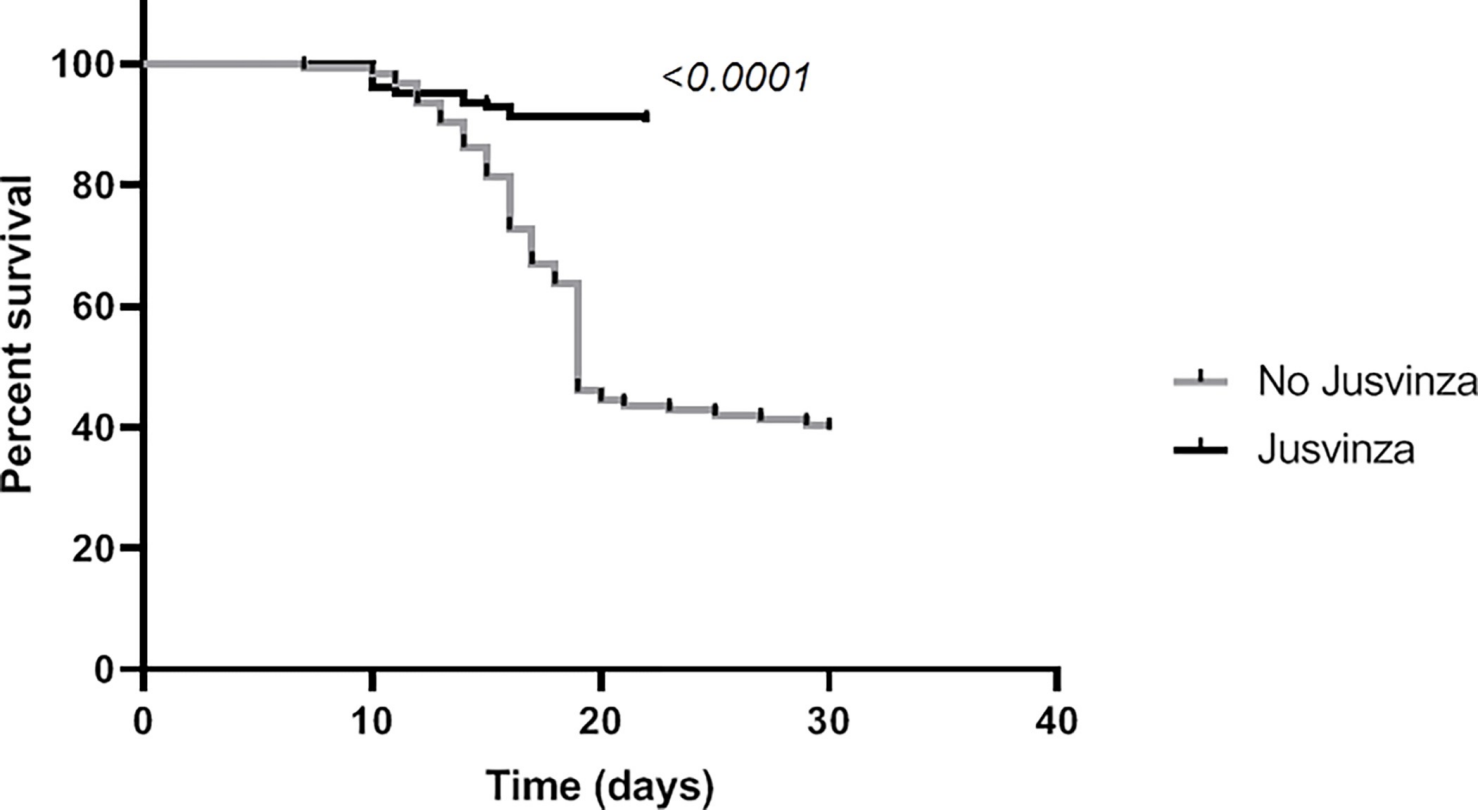
Survival time between the two groups (Kaplan-Meier curve). Gray line correspond to the group patients did not received Jusvinza and the black line correspond to the group patients exposure to Jusvinza, maximum time on ICU from each group was of 30 and 22 days, respectively. Statiscally analysis was made using a Log-rank (Mantel-Cox) test. p < 0.05 was considered statistically significant.

In addition, survival time between the two groups was compare by a Kaplan-Meier curve (Fig 2). This curve illustrates there were no deaths in the first six days. Deaths increased after day 10. However, the group exposed to Jusvinza had a statistically superior survival.

Furthermore, no adverse events described had a causal relationship with Jusvinza according the data extracted from CR.

### Secondary endpoints

Table 2 shows the values of the mean arterial blood pressure, heart rate and respiratory rate in the two groups. We observed a significant improvement in these three vital signs at 48 hours, 72 hours and 7 days in the group treated with Jusvinza. In contrast, these signs worsen in patients not receiving Jusvinza.

**Table 2:** Vital signs and ventilation parameters.

	Reference range	No Jusvinza							Jusvinza						
		T0	48h	*p value*	72h	*p value*	Day 7	*p value*	Before treatment	48h	*p value*	72h	*p value*	Day 7	*p value*
**Sign vitals**															
Mean arterial pressure (mmHg)	70–100	66 (49–83)	66 (55–73)	***<0*.*0001***	66 (45–73)	***<0*.*0001***	66 (55–73)	***<0*.*0001***	66 (53–116)	85 (73–98)	***<0*.*0001***	83 (73–98)	***<0*.*0001***	83 (73–98)	***<0*.*0001***
Heart rate (bpm^a^)	60–100	111 (78–165)	115 (110–150)	*0*.*3137*	130 (110–227)	***<0*.*0001***	130 (110–227)	***<0*.*0001***	119 (98–142)	88 (76–100)	***<0*.*0001***	85 (76–135)	***<0*.*0001***	85 (76–135)	***<0*.*0001***
Respiration rate (bpm^b^)	12–16	33 (29–44)	38 (29–46)	*0*.*0584*	39 (34–45)	***<0*.*0001***	39 (34–45)	***<0*.*0001***	35 (21–40)	22 (18–38)	***<0*.*0001***	19 (18–38)	***<0*.*0001***	19 (18–38)	***<0*.*0001***
**Ventilation parameters**															
SO2 (%)	93–100	87 (82–90)	88 (80–92)	*>0*.*9999*	83 (80–99)	***<0*.*0001***	83 (80–99)	***<0*.*0001***	90 (85–94)	95 (94–97)	***<0*.*0001***	95 (82–96)	***<0*.*0001***	95 (82–96)	***<0*.*0001***
PO2 (mmHg)	75–100	55 (49–61)	54 (44–79)	*0*.*0624*	54 (52–62)	***<0*.*0001***	54 (52–62)	***0*.*0042***	54 (50–59)	68 (66–78)	***<0*.*0001***	79 (51–88)	***<0*.*0001***	79 (51–88)	***<0*.*0001***
P/F	>300	183 (163–203)	132 (69–579)	***<0*.*0001***	89 (65–890	***<0*.*0001***	77 (65–890)	***<0*.*0001***	183 (167–197)	220 (110–259)	***0*.*0002***	263 (73–300)	***<0*.*0001***	250 (76–300)	***<0*.*0001***

**a**- beats per minute

**b**-breaths per minute. Values are represented at median per range. No Jusvinza N = 124; Jusvinza N = 125

**p values** resulted from comparisons at 48h (hours), 72h and day 7, regarding to T0. Time zero (T0) corresponded to parameter values in the first blood draw from patients, when they were admitted at the ICU. **p < 0.05** was considered statistically significant, Friedman’s test, and Dunn’s multiple comparisons test

All the patients included in this study had ARDS, evidenced by a PO2 / FiO2 (P/F) ratio ≤ 300 mmHg, according to the Berlin criteria [21]. SO2, PO2 and P/F significantly improved at 48 hours, 72 hours and 7 days in the group treated with Jusvinza. However, these ventilatory parameters worsened significantly in patients not treated with Jusvinza (Table 2).

**Fig 3:**
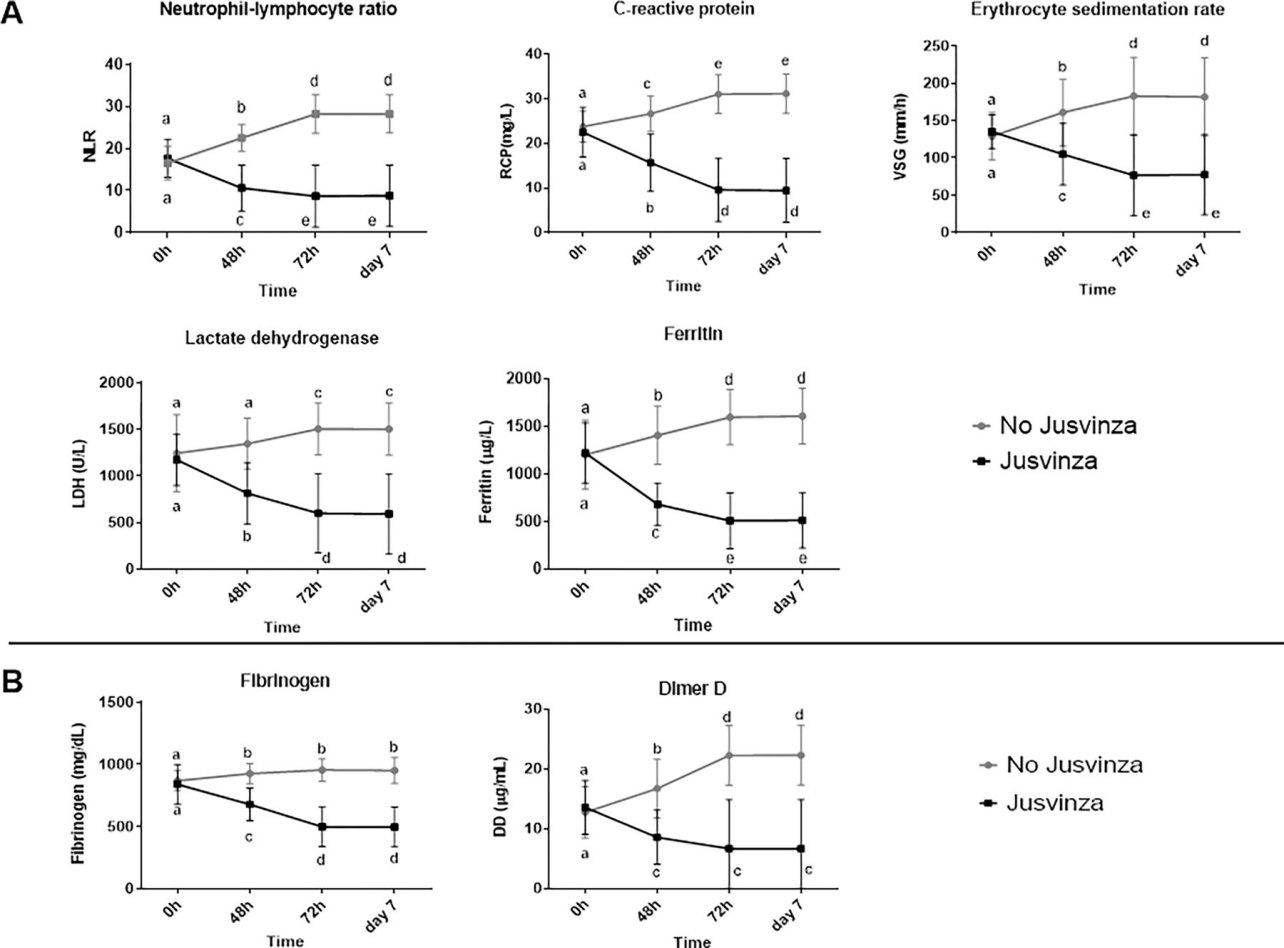
Inflammatory (A) and coagulopathy (B) biomarkers at T0 and 48h, 72h and 7 days. NLR, CRP, ERS, LDH and ferritin values were abstracted from HR. Graphs and statistical analysis where made at GraphPad Prism 8.02. Biomarker values are represented as the media ± standard deviation. Gray line correspond to the group patients did not receive Jusvinza (N = 124) and the black line correspond to the group patients exposure to Jusvinza (N = 125). Statistical differences were analyzed using a Kruskal-Wallis and Dunn post-test. Significant statistically difference (***p<0*.*05***) about time and between both groups, are represented with different letters.

Biomarkers associated with inflammation: NLR, CRP, ERS, LDH and ferritin are shown in Fig 3A. The levels of these five biomarkers were similar in the two groups of patients at T0. Serum concentration of these biomarkers decrease significantly at 48 hours, 72 hours and at 7 days in patients treated with Jusvinza, compared to T0. Likewise, we observed a significant decrease in the levels of these biomarkers in the group treated with Jusvinza at 48 hours, 72 hours and 7 days, compared to the group without Jusvinza. A similar behaviour was observed for the biomarkers associated with coagulation: DD and fibrinogen (Fig 3B).

**Fig 4:**
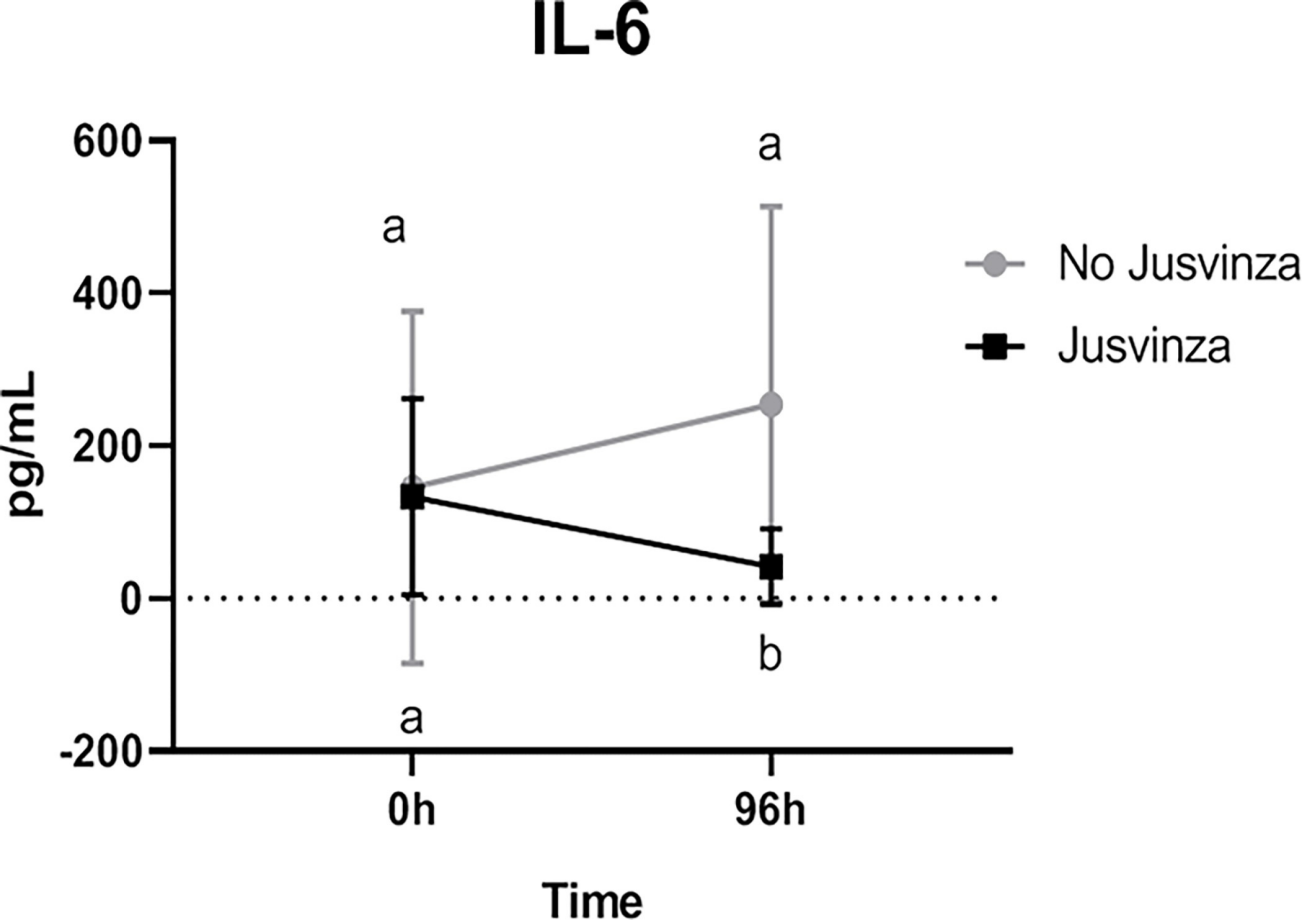
Effect of Jusvinza on cytokine IL-6 at T0 and 96 hours. Il-6 values were abstracted from HR. IL-6 values are represented as the media ± standard deviation. Gray line correspond to the group patients did not receive Jusvinza (N = 8) and the black line correspond to the group patients exposure to Jusvinza (N = 13). Differences were analyzed using a Wilcoxon matched-pairs signed rank test. Significant statistically difference (***p<0*.*05***) about time and between both groups, are represented with different letters.

Furthermore, IL-6 levels were analysed independently in the two groups. IL-6 levels were significantly higher (P = 0.0154) in the group that did not receive Jusvinza than in the group treated with Jusvinza, at 96 hours after starting the treatment. However, no significant difference was detected between the two groups at T0 (Fig 4).

## Discussion

Hyperinflammation caused by SARS-CoV-2 is closely linked with the progress of coagulopathies and a high risk of thrombosis. Several authors postulate that COVID-19 induces a prothrombotic state, attributable to the combination of the hyperinflammation and hypoxia [6–8, 33]. Pathological studies indicate the presence of numerous large and small thrombi in COVID-19 patients [34]. Pulmonary endothelial cells contribute to the start and spread of ARDS in COVID-19 patients, by altering the integrity of the vessel barrier, which generates a procoagulative effects, inducing vascular inflammation and the consequent infiltration of inflammatory cells [35]. In Cuba, Jusvinza is used to treat COVID-19 patients with signs of hyperinflammation, under an Emergency Use Authorization [13].

This drug induces a clinical and radiological improvement in seriously and critically ill patients with COVID-19, linked with the decrease of inflammation biomarkers [21].

This observational investigation describes the clinical outcome and variations of several biomarkers associated with inflammation and coagulation in two groups of patients: a group receiving Jusvinza and another one without it.

The treatment with Jusvinza was determined by availability of this drug, during the period in which this study was carried out. This study coincided with the maximum peak of COVID-19 infections in Cuba [36].

Most patients included in this study had comorbidities and many of them had two or more of these pathological conditions that complicate the course of COVID-19 [37–39].

This clinical improvement is sustained over time, evidenced by a survival of 90.4% of the patients treated with Jusvinza, which was significantly higher than the survival of patients not treated with this peptide (39.5%). The primary endpoint of this study was death during the follow-up through August 30, 2021. The results indicated that the survival of patients exposed to Jusvinza at follow-up was significantly higher than the group not treated with this peptide. Likewise, patients not treated with Jusvinza had a longer ICU stay than those exposed to Jusvinza.

These results coincide with a retrospective observational study that included all Cuban patients treated in ICU from March 11 to July 30, 2020. This study showed that treatment with Jusvinza decreased the probability of dying from 80% to about 25% [40].

In addition, monitoring data from the Ministry of Public Health of Cuba in UCI from January to August, 2021, showed the recovery rate of the Jusvinza (n = 676) cohort was 85% compared with 35% in a group not treated with Jusvinza (n = 195). This last group received standard of care, but not received Jusvinza due to non-disposal of this drug.

Similar to previous reports, signs of immunosuppression and adverse effects associated with Jusvinza were not observed in this observational study. The treatment with Jusvinza has been well tolerated and safe for patients with rheumatoid arthritis included in a phase-I clinical trial [18]. Likewise, critically ill patients with COVID-19 treated with Jusvinza did not show symptoms of possible immunosuppression and no adverse events associated with this drug were reported during treatment or in the follow-up stage [21].

On the other hand, this observational study shows that in Jusvinza-treated patients there was a significant reduction in hypoxia. Furthermore, this study showed that Jusvinza reduces inflammation, since there was a significant decrease in NLR. This biomarker has been widely used as a predictor of inflammation in COVID-19 patients. COVID-19 patients that progress to severe stages are characterized by marked lymphopenia and neutrophilia [41, 42].

Likewise, the treatment with Jusvinza significantly reduces the inflammation biomarkers. These results indicate that Jusvinza could be able to control hyperinflammation.

Notably, a decrease in circulating IL-6 was found in patients treated with Jusvinza compared to the control group. High levels of IL-6, associated with disease severity, have been widely reported in COVID-19 patients [43, 44]. This cytokine has been involved in the progression of virus infections and it is considered one of the most important cytokines in infections, along with IL-1 and TNF-α [45]. The reduction of IL-6 indicates the decline of inflammation.

Furthermore, Jusvinza significantly reduces two biomarkers associated with coagulation. High serum concentrations of DD and fibrinogen are directly correlated with severe forms of COVID-19 and a poor prognosis for patients [6]. The reduction of these biomarkers shows that the peptide could prevent venous and arterial thrombotic events in patients with COVID-19. All results must be confirmed with controlled and randomized clinical trials.

The molecular mechanism by which SARS-CoV-2 induces hypercoagulation in patients is not fully understood, but it is closely related to hyperinflammation. SARS-CoV-2 can infect endothelial cells and induce an immune response, which affects the endothelium and causes the activation of neutrophils, with the consequent formation of extracellular neutrophil traps (NET), complement deposition and platelet activation [46]. Besides, this virus induces pyroptosis of endothelial cells, which increases the secretion of pro-inflammatory cytokines. These processes can induce the development of systemic thrombotic events [47]. In this sense, we are studying the effect of Jusvinza on NET.

We have shown that the treatment with Jusvinza decreases NLR and helps restore neutrophil and lymphocyte counts in critically ill COVID-19 patients. Moreover, Jusvinza decreases inflammatory cytokines and induces an increase in regulatory T cells, which control the extent of inflammation [21]. The molecular and cellular effects induced by Jusvinza could inhibit hyperinflammation and vascular damage that cause the thrombotic processes.

## Conclusions

The survival of the patients treated with Jusvinza was significantly higher than in the patients not treated with this peptide. This study shows that the treatment with Jusvinza was associated with a decrease in IL-6 and several biomarkers of inflammation and coagulation in critically ill patients with COVID-19. The anti-inflammatory effect of Jusvinza constitutes an attractive therapeutic approach for a wide range of diseases characterized by hyperinflammation.

## Supporting information

S1 Checklist TREND statement checklist. (PDF)

S1 Method Clinical protocol. Protocol of the Retrospective Study of Jusvinza. (English version).
(PDF)

S2 Method Clinical protocol (Spanish version). (PDF)

S1 Table Supporting information files. Serial analyses of clinical parameters and plasma biomarkers at T0, 48 hours and days 7.
(PDF)
