# Supplementary material for: Jusvinza, an anti-inflammatory drug derived from the human heat-shock protein 60, for critically ill COVID-19 patients. An observational study
Source: PLoS One. 2023 Feb 2;18(2):e0281111. doi: 10.1371/journal.pone.0281111 (PMC9894446; doi:10.1371/journal.pone.0281111)
Supplement: S1 Method — Protocol of the Retrospective Study of Jusvinza. (English version). (PDF) [file pone.0281111.s002.pdf]

The information contained in this document is the property of the CIGB. It cannot be reproduced, published or revealed to others without the explicit authorization of the promoter.

PROTOCOL OF THE RETROSPECTIVE STUDY

"VALENTÍA" STUDY

"Retrospective observational study of the administration of Jusvinza according to the action protocol of CUBA, for the management of COVID-19 at the Dr Luis Diaz Soto Hospital"

Study code: INV/CIGB-258/003/21

Version: 1.0

Havana, May 4, 2021

INDICE

SUMMARY *4*

LIST OF ABBREVIATIONS USED AND DEFINITION OF THE TERMS **6**

I. GENERAL INFORMATION **8**

1.1. Title of the clinical trial: “Retrospective observational study of the administration of Jusvinza according to the action protocol of CUBA, for the management of COVID-19 at the Dr Luis Díaz Soto Military Hospital” 8

1.2. Code: INV/CIGB-258/003/21 8

1.3. Sponsors 8

1.4. Participating institution 8

1.5 Persons in charge of the organization and implementation of the study, data collection and processing – CIGB, Havana. .8

II. INTRODUCTION **10**

2.1. Main data on the problem and its context. 10

III. OBJECTIVES **12**

3.1. General Objective *12*

3.2. Specific objectives *12*

3.3 Hypothesis *13*

IV. MEDICAL DEONTOLOGY **13**

4.1. Ethics and Review Committee (ERC) / Ethics for Scientific Research Committee (ESRC) 13

4.2. Ethical issues for the implementation of the trial 13

4.3. Instructions for obtaining the informed consent 14

4.4. Ethical responsibilities of all participants in the study 14

V. GENERAL CONCEPT **15**

5.1 Design of the trial 15

5.2 Identification of the patients 15

5.3 Factors that may be introduced to reduce bias 15

VI. SELECTION OF THE SUBJECTS **15**

6.1. Universe of patients 15

VII. TREATMENTS STUDIED **16**

VIII. EVALUATION VARIABLES **16**

8.1. Protocol variables 16

8.1.1. Main variables *16*

8.1.2. Secondary variables *17*

8.1.3. Control variables *17*

8.2. Criteria for the success or failure of the individual, and of the therapy 18

IX. ADVERSE EVENTS **18**

9.1 Adverse reactions that may be observed and methods used to record them 18

X. DATA COLLECTION AND HANDLING **19**

10.1. Information recording form 19

10.2. Procedure for conserving the information 20

XI. STATISTICS **20**

11.1 Number of patients planned 20

11.2 Plan for the statistical analysis 20
11.2.1 Set of data analyzed *20*
XII. ASSURANCE 20
XIII. GENERAL SCHEDULE 22
Stage 22
XIV. PRACTICAL CONSIDERATIONS 23
14.1. Distribution of the duties and responsibilities in the protocol. 23
14.1.1. Responsibilities of the promoter (CIGB) *23*
14.1.2. Monitors *23*
14.3.3. Specialists in charge, and their designated research team *23*
14.4. Procedures for the flow of documents 24
14.5. Considerations on the problems of confidentiality, dissemination of results and other legal issues 24
XVI. REFERENCES 26

SUMMARY:

Title	"Retrospective observational study of the administration of Jusvinza according to the action protocol of CUBA for the management of COVID-19 at the Dr Luis Diaz Soto Hospital"
Patient population	Patients ≥ 19 years of age, who were positive to SARS-CoV-2 by RT-PCR, classified as moderate, severe and critically ill patients, according to the action protocol of Cuba for the management of COVID-19
Rationale of the study	Patients with COVID-19 that progress toward the more severe stages of the disease show a marked hyper-inflammation that can lead to cardiovascular collapse and multiple organ failure and produce death. In this setting, we recommend the identification and treatment of hyper-inflammation with the aim of reducing mortality. In Cuba we use the drug Jusvinza for the treatment of moderate, severe and critical COVID-19 patients. Jusvinza is an immunomodulator peptide with anti-inflammatory properties.
Objectives of the study	**General objective:** To observe its effectiveness in the reduction of hyper-inflammation and the safety of the administration of Jusvinza in patients having COVID-19 whose condition was classified as moderate, severe and critical.<br>**Specific objectives**<br>• Describe the clinical progress of the high-risk patients towards severe or critical stages or death<br>• Describe the progress of the inflammatory markers of the patients included in the study.<br>• Describe the radiologic progress of the patients included in the study.<br>• Describe the adverse events.
Hypothesis	On considering that with the extensive study of the use of Jusvinza in Cuba, 85 % of the severe patients recovered, we expect that 90% of the high-risk patients treated with Jusvinza will not progress to the severe conditions of the disease and will recover.
Main variables	Percentage of high-risk, severe and critical patients recovering after the treatment with Jusvinza.
Secondary variables	1. Describe the clinical evolution of the patients.<br>2. Describe the radiological evolution.<br>3. Quantify the inflammatory biomarkers in the serum (NLR, C-reactive protein, erythrocyte sedimentation, Ferritin, LDH, D-dimer, CPK, ASAT, ALAT).<br>4. Quantify the cytokines: IL-6, TNFα and IL-10.<br>5. Quantify the percentage of lymphocytes CD4, CD8 and Treg.<br>6. Presence and characterization of adverse events.

Design of the study	Retrospective, observational, monocentric study<br>We will collect the retrospective information of each patients using the clinical records as the source of primary information, as well as clinical laboratory results and radiologic imaging stored in the GALEN program of the participating institution.
Number of patients	We will include all positive patients to SARS-CoV-2 by RT-PCR with the available information, classified as of high-risk, severe and critical conditions, who were prescribed the use of Jusvinza.
Frequency and duration of the therapy	According to the indications of the Action Protocol
Drug under study	Drug: Jusvinza (2.5 mg or 1.25 mg), intravenous administration.
Safety evaluation	• Symptoms<br>• Vital signs<br>• Analytical results<br>• Imaging results

LIST OF ABBREVIATIONS USED AND DEFINITION OF THE TERMS

GCP: Good Clinical Practices: A standard for the design, conduction, implementation, monitoring, auditing, recording, analysis and reporting of clinical trials that offer the assurance that the data and the results reported are credible and accurate, and that the rights, integrity and confidentiality of the subjects of the study are protected. (Extracted from regulation 21 – 08: requirements for the authorization and modification of clinical trials. CECMED)

CECMED: Center for the State Control of Drugs, Medical Equipment and Devices.

ESRC / ERC: Ethics for Scientific Research Committee / Ethics and Review Committee.

CIGB: Center for Genetic Engineering and Biotechnology.

DRF: Data Recording Form.

Adverse event: It is defined as any unfavorable medical incident occurring to a subject participating in a clinical trial after the administration of a pharmaceutical product. This incident may not necessarily have a causal relationship with the treatment. An adverse event may therefore be an unfavorable or unexpected sign (e.g. an abnormal laboratory finding), symptom or disease that is temporarily associated with the use of a medicinal product. (Extracted from the Guidelines on Good Clinical Practices, CECMED, 2000)

Unexpected adverse event: Any adverse event in which the specificity or severity is not consistent with the risk information described in the protocol or in the researcher's handbook (if there is one). It also refers to an adverse event that has not been previously observed.

Jusvinza: immuno-modulating peptide CIGB-258.

MINSAP: Ministry of Public Health.

RT-PCR: Real Time Polymerase Chain Reaction.

Adverse reaction: It refers to an adverse event that is considered to be related in a causal manner with the research product; this includes overdose and interactions with other drugs. Any undesired harmful response produced by a pharmaceutical product at any dose, should be considered an adverse reaction to drugs. One well accepted definition of a reaction to drugs is found in the Technical Report of the World Health Organization (Series No. 850, 1995) that states: "and adverse reaction is a harmful and

unintended response to a pharmaceutical product that occurs at a dose that is normally used in persons for prophylaxis, diagnosis, therapy, or to modify a physiological function. In clinical trials, the damage caused by overdose, abuse or dependence, and interactions with other products, should be considered adverse reactions".

SARS-CoV: Severe Acute Respiratory Syndrome-Coronavirus.

I. GENERAL INFORMATION

1.1. Title of the clinical trial: "Retrospective observational study of the administration of Jusvinza according to the action protocol of CUBA, for the management of COVID-19 at the Dr Luis Díaz Soto Military Hospital"

1.2. Code: INV/CIGB-258/003/21

1.3. Sponsors

❖ Center for Genetic Engineering and Biotechnology (CIGB).
Biotechnological and Pharmaceutical Industries Group, BioCubaFarma.
Ave. 31 e/ 158 y 190, Cubanacán, Playa, Havana, Zip code 6162, P.O. Box 11600, Cuba.
☎ (53-7)-2716022; Fax (53-7)-2716070 / 2736008; Web: http://www.cigb.edu.cu

1.4. Participating institution

- " Dr. Luis Díaz Soto" Hospital, Havana.

1.5 Persons in charge of the organization and implementation of the study, data collection and processing– CIGB, Havana.

- María del Carmen Domínguez Horta, PhD: Doctor of Biological Sciences; Researcher and Full Professor.
- Mabel Hernández Cedeño, MS: Researcher of the Biomedical Research Division
- Anabel Sierra, BS: Researcher of the Biomedical Research Division
- Dr Deylis Chacón: Head of the Emergency Unit of the Central Military Hospital "Dr Luis Díaz Soto"
- Mj. Dr Leticia del Rosario. Second Head of the Emergency Unit of the Central Military Hospital "Dr Luis Díaz Soto"

1.6. Monitors – Clinical Research Division of the CIGB, Havana

- Mabel Hernández Cedeño, MS: Researcher of the Biomedical Research Division
- Anabel Sierra, BS: Researcher of the Biomedical Research Division

1.7. Persons in charge of data management and statistical analysis – CIGB, Havana.

- Mabel Hernández Cedeño, MS: Researcher of the Biomedical Research Division
- Anabel Sierra, BS: Researcher of the Biomedical Research Division

1.8. Consultant – CIGB, Havana

- Gerardo Enrique Guillén Nieto, PhD: Doctor of Biological Sciences; Bachelor of Science and Master's Degree in Chemical Sciences; Researcher, Professor and Senior Academician. Director of Biomedical Research; Secretary of the Scientific Council of the CIGB, Havana.

1.9. Ethics and Review Committee

The biomedical study will be carried out retrospectively, taking into account the available data from the clinical records of the patients treated with Jusvinza.

The source of information for data collection are the Clinical Records and the clinical laboratory results and the available imaging results of each patient through the GALEM program. The approval from the Ethics Committee will be obtained for accessing the information of the patients.

The protocol will be presented in the public registry of clinical trials of CENCEC.

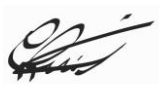

II. INTRODUCTION

2.1. Main data on the problem and its context.

Patients having COVID-19 that progress towards severe and critical conditions present a marked hyper-inflammation. This stage of the disease is mediated by a high concentration of pro-inflammatory cytokines, a biological event known as the “cytokine storm”. As the hyper-inflammation progresses, these patients can reach cardiovascular collapse and multiple organ failure that would lead to death (i,ii)

In this setting, the identification and treatment of hyper-inflammation is recommended, with the aim of reducing mortality. In Cuba, we use the drug named Jusvinza for the treatment of moderate, severe and critical COVID-19 patients. Jusvinza is an immuno-modulator peptide with anti-inflammatory properties. The peptide was developed at the CIGB for the treatment of autoimmune diseases, specifically for rheumatoid arthritis (RA) (iii).

This peptide was shown to increase the frequency of regulatory T cells (Treg) with the phenotype $CD4+CD25^{high}Foxp3+$ in *ex vivo* assays with peripheral blood mononuclear cells (PBMC) from patients with RA (rheumatoid arthritis), but not in healthy donors; these cells have a suppressing activity (iv). Furthermore, on administrating the drug through the subcutaneous route, the peptide induces a significant increase of the Treg cell population showing phenotype CD4+Foxp3+ in the lymphatic nodes, in contact with the administration site, and in the spleen of BALB/c mice. Moreover, this peptide efficiently inhibits inflammation in arthritis models (v).

On the other hand, the pharmacokinetic and biodistribution studies through three routes, intravenous, intradermal and subcutaneous routes, demonstrated that the peptide has a broad distribution towards different organs, which include the gastrointestinal tract, liver, lungs, etc. The maximum concentration in the blood is reached after half an hour and its clearance occurs at approximately 6 hours (vi). Toxicology studies in three animal species demonstrate that the peptide has a good safety profile.

These results at the preclinical research stage resulted in the authorization granted by the Regulatory Authority for Drugs, Medical Equipment and Devices of the Republic of Cuba (CECMED) for the evaluation of the peptide in a phase I clinical trial in patients with RA (RPCEC00000238). In this phase I clinical trial, the product showed its safety, pharmacokinetic profile and there was preliminary evidence of its therapeutic effect (vii,viii, ix). At present, we are processing the results of a Phase II

clinical trial in patients with RA (RPCEC00000230), to evaluate the efficacy and safety of this therapeutic candidate. This is a controlled, randomized and triple blind trial with 187 patients. We have observed a good safety profile.

This set of results endorsed the granting for the compassionate use of Jusvinza by CECMED in the treatment of critical patients with COVID-19 (RPCEC00000313). The action protocol with Jusvinza was started on March 31, 2020, in the intensive care unit (ICU) of the Luís Díaz Soto Military Hospital, of Havana. The medical specialists described in their reports that as of 48 hours of the treatment with Jusvinza, the patients started showing clinical, gasometrical and radiologic improvement. These 12 patients were extubated and they recovered; they continue under a strict surveillance in their health areas. The laboratory parameters indicated that the patients had lymphopenia and a trend towards neutrophilia before starting the treatment. However, during the course of the treatment, the levels of lymphocytes and neutrophils reached their normal values. At the same time, the markers associated to hyper-inflammation (C-reactive protein, ferritin, dehydrogenase lactate, fibrinogens, creatinine and transaminases) were normalized during the treatment. The concentration of the three cytokines involved in the cytokine storm (IL-6, TNF-α, IL-1 and IL-10) were normalized during the course of the treatment (x). These results and the safety profile of Jusvinza enabled led to the authorization for its use in the treatment of severe COVID-19 patients, and the inclusion of Jusvinza will be approved for the national treatment protocol in severe and critically ill patients.

The introduction of Jusvinza in the national protocol approved by the Ministry of Public Health for the treatment of severe and critical cases of COVID-19, took place on April 27, 2020, as well as the extension of its use in all Cuban hospitals with COVID-19 cases. The use of Jusvinza has had an impact in decreasing the lethality rate in Cuba. The lethality rate in April of 2020 in Cuba was of 4.16 (xi) and in March of 2021 it was of 0.46 (xii).

The treatment with Jusvinza has been safe for the patients. Molecular studies made in Cuba, when extending the use of Jusvinza to all hospitals with COVID-19 patients, have confirmed the results obtained during the compassionate use study. It was demonstrated that the treatment with Jusvinza is able to reduce hyper-inflammation in COVID-19 patients. The administration of Jusvinza in severe patients prevents the progression of these cases to a critical phase of the disease. The effectiveness of the treatment in severe patients surpasses 86%. The levels of C-Reactive Protein (CRP) decrease

significantly in patients at 72 hours after the treatment. A significant inverse correlation was found between the improvement of the ventilating parameters and the CRP in critical patients with invasive mechanical ventilation. Other inflammation biomarkers such as ferritin, dehydrogenase lactate, creatinine and transaminases, decrease significantly. Several studies report that these patients are characterized because of their neutrophilia and lymphopenia, with a neutrophil/lymphocyte ratio (NLR) above 5.1 (xiii). The treatment with Jusvinza reduced NLR to normal values. Furthermore, it reduced the levels of Calprotectin, a protein secreted by monocytes and neutrophils during inflammatory processes (xiv). The reduction of the Calprotectin was significantly correlated to the decrease of neutrophils. Moreover, reports show that the deregulation of the immune response in COVID-19 patients is associated to an increase in Granzyme B and Perforin, and a decrease in the percentage of Treg (xv, xvi). The results of our patients indicate a significant decrease of Granzyme B and Perforin, at 96 hours of the treatment, coinciding with the decrease of interleukins IL-6, IL-10 and TNF-α. The percentage of Treg increased at 48 hours of the treatment in the severe patients studied (xvii). The increase of the Treg in COVID-19 patients is consistent with the mechanism of action of Jusvinza in patients with RA (iii,iv).

The overall results made it possible to conclude that the treatment with Jusvinza decreases the hyperinflammation that characterizes the patients with COVID-19.

III. OBJECTIVES

3.1. General Objective

- To observe its effectiveness in the reduction of hyper-inflammation and the safety of the administration of Jusvinza in patients having COVID-19 whose condition was classified as moderate, severe and critical.

3.2. Specific objectives

1. Describe the clinical progress of the high-risk patients towards severe or critical stages or death
2. Describe the progress of the inflammatory markers of the patients included in the study.
3. Describe the radiologic progress of the patients in the study.

4. Describe the adverse events.

3.3 Hypothesis

On considering that with the extensive study of the use of Jusvinza in Cuba, 85 % of the severe patients recovered, we expect that 90% of the high-risk patients treated with Jusvinza will not progress to the severe conditions of the disease and will recover.

IV. MEDICAL DEONTOLOGY

4.1. Ethics and Review Committee (ERC) / Ethics for Scientific Research Committee (ESRC)

To start the implementation of the clinical trial protocol in the service unit, it is necessary to obtain the declaration of the Institutional Ethics Committee, which grants the certification after making its assessments and corresponding analyses that the document (version 1.0):

- ✓ Complies with the Helsinki Declaration (Ethical principles for medical research in humans, adopted by the World Medical Assembly, Fortaleza, Brazil, 2013).
- ✓ Complies with the norms and ethical criteria established in national and international ethics and legal regulations code in force in Cuba (Guidelines for Good Clinical Practices, CECMED 2000, Cuba; Guide to Good Clinical Practices of the International Conference for Harmonization – ICH E-6).
- ✓ Includes the protection of the rights and wellbeing of the patients involved.
- ✓ Appropriately describes the selection criteria of the patients.

4.2.Ethical issues for the implementation of the trial

The retrospective study is properly endorsed from the ethical viewpoint due to the following reasons:

a. It is approved by the ERC/ESRC. At the same time, as a contribution to the transparency of the research, the protocol will be published in the Cuban Public Registry of Clinical Trials.

b. A clinical intervention on the patients will not be carried out; their evolution will be observed as soon as they are treated with the Jusvinza drug. There is evidence of the reduction of inflammation in patients with COVID-19 treated with Jusvinza. The treatment with Jusvinza has been safe for the

patients. The molecular studies made in Cuba, during the extension of the use of Jusvinza to all hospitals with COVID-19 patients, have confirmed the results obtained during the compassionate use study, demonstrating that the Jusvinza treatment is able to reduce inflammation in COVID-19 patients. The administration of Jusvinza in severely ill patients prevents the progress to the critical stage of the disease. The effectiveness of the treatment in severely ill patients surpasses 85%.

c. The results obtained from processing the information of patients treated with Jusvinza will produce a benefit for the COVID-19 patients that will undergo the treatment in the future.

d. The integrity of the participants in the research will be respected, ensuring the confidentiality of all the data of the patients.

4.3. Instructions for obtaining the informed consent

Does not apply.

4.4. Ethical responsibilities of all participants in the study

Researcher: Offers access to the information of the patient recorded in the Clinical Records

Institution: Ensures the maintenance and appropriate use of the facilities by the researchers and submits the protocol to the approval of the Ethics and Review Committee (facilitated by the researcher in charge).

Research team: Ensures the compliance of the assigned responsibilities.

Promoter: Ensures the reliable collection of the information, while providing confidentiality to the data of the patients.

Ethics and Review Committee: Reviews and approves the protocol of the trial, ensuring the protection of the rights of the patients involved in the study and providing the public assurance of that protection. Verifies the progress of the study and the adherence of the researchers to the protocol.

V. GENERAL CONCEPT

5.1 Design of the trial

A retrospective, observational, monocentric study will be carried out with the primary aim of evaluating the effect of Jusvinza in the reduction of inflammation and the safety of its intravenous administration.

All patients with available information that have been treated with Jusvinza, according to the action protocol, between March 21 and July 29 of 2021, will be included.

5.2 Identification of the patients

Each patient will be identified by a code that indicates the "consecutive inclusion number", followed by the initials of the name/or names and surnames of the patient. This identification must be contained in the documents corresponding to each patient. For example, for the first patient included in the study, the identification code is 001-XYZM, where X is the initial of the first name, Y is the initial of the middle name (if there is one), Z is the initial of the first surname and M is that of the second surname.

5.3 Factors that may be introduced to reduce bias

- Before preparing this protocol, there was an exchange of ideas with specialists linked to the management of the use of Jusvinza in COVID-19 patients at the Central Military Hospital Dr Luis Diaz Soto and the researchers of the Clinical Research Division, and of the Biomedical Research Division of the CIGB. The clinical experiences of the use of Jusvinza in COVID-19 patients were presented and discussed. This has made it possible to endorse the research and define the present experimental design.
- The analysis and discussion of the protocol, and its proficiency, will favor the adherence and compliance of the GCP by all researchers.

VI. SELECTION OF THE SUBJECTS

6.1. Universe of patients

The universe is formed by adult patients that live permanently in Cuba (with full constitutional rights), that are positive to SARS-CoV-2 according to the RT-qPCR, who are hospitalized at Dr Luis Díaz Soto hospital and have been prescribed the treatment with Jusvinza, according to the action protocol, in the period of March 21 to July 29, 2021.

6.2. Inclusion criteria

1) Patients ≥ 19 years of age.

2) Patients positive to SARS-CoV-2 according to the confirmatory RT-qPCR test, who are either moderately, severely, critically ill or dead, and have been prescribed the treatment with Jusvinza, in the period from March 21 to July 29 of 2021.

3) Patients that have not died in the first 48 hours of hospitalization.

4) Patients that have not been included in other clinical trials.

VII. TREATMENTS STUDIED

- **JUSVINZA®**

It is a sterile lyophilized powder containing 2.5 mg or 1.25 mg, in 2R glass flasks.

Active ingredients: Immunomodulator CIGB-258 peptide, obtained by chemical synthesis at the Center for Genetic Engineering and Biotechnology (CIGB, Havana, Cuba).

The composition of each vial is shown in the following table:

Component	Amount	Function	Quality Standard
CIGB-258 peptide	2.5 mg o 1.25mg	Immunomodulator	NP 4380C Injectable according to the manufacturer
Sucrose	20.0 mg	Stabilizer	NP 252 / USP
Acetic acid 50 mM. pH 4	3.0 mg	Acid, constituting the buffer	NP 020 / USP

Action: anti-inflammatory and immunomodulator effect.

VIII. EVALUATION VARIABLES

8.1. Protocol variables

8.1.1. Main variables

- **Evaluation of the anti-inflammatory effect:**

- ✓ We will calculate the percentage of high-risk patients recovering after a seven-day treatment with Jusvinza.
- ✓ We will calculate the percentage of severely ill patients recovering after a ten-day treatment with Jusvinza.
- ✓ We will calculate the percentage of critically ill patients recovering after a fifteen-day treatment with Jusvinza.

8.1.2. Secondary variables

1. Describe the clinical evolution of the patients.
2. Describe of the radiological evolution of the patients.
3. Quantify the inflammatory biomarkers in the serum (NLR, C-reactive protein, erythrocyte sedimentation, Ferritin, LDH, dimer-D, CPK, ASAT, ALAT).
4. Quantify the cytokines: IL-6, TNFα e IL-10.
5. Quantify the percentage of lymphocytes CD4, CD8 and Treg.
6. Describe the adverse events.

8.1.3. Control variables

Because of their possible effect on the therapeutic response, we will consider the following:

a. Adherence of the patient to the treatment.
b. Personal pathological history.
c. Age (years)
d. Sex (male / female).
e. Skin color (white, non-white).
f. Symptomatology (symptomatic, asymptomatic, pre-symptomatic)
g. Time taken between the positive diagnosis of SARS-CoV-2 and the start of the first symptoms or contact.
h. Previous treatments and those concomitant with the research product (anti-viral drugs, basic diseases)

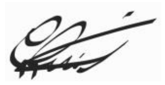

i. Body mass index.

j. Toxic habits (smoking, alcohol consumption).

8.2. Criteria for the success or failure of the individual, and of the therapy

□ We will consider an **individual success** if the patient recovers; if there are no severe adverse events with a demonstrated causality.

□ We will consider a **therapeutic success** if the hypothesis is demonstrated, in which 90% of the high-risk patients treated with Jusvinza do not reach the severe phases of the disease and then recover.

□ We will consider an **individual failure** when a patient progresses toward a more severe condition or death and if there is a definitive interruption of the treatment due to the presence of severe adverse events, with a causal relationship attributable to the product under study.

□ We will consider a **therapeutic failure** when patients progress towards the more severe stages of the disease.

IX. ADVERSE EVENTS

9.1 Adverse reactions that may be observed and methods used to record them

<u>The adverse events are classified</u> according to type, duration, intensity, causality relationship, actions taken, and results, <u>in the following grades</u>[xviii]:

Grade **1** ***(Mild)***	Asymptomatic or symptoms of mild intensity. Only clinical observation or diagnostic observation are needed. Does not require treatment.
Grade **2** ***(Moderate)***	Requires minimal intervention, i.e. local or non-invasive.
Grade **3** ***(Severe)***	Not immediately life-threatening for the patient. Requiring hospitalization (or its prolongation). Disabling
Grade **4** ***(Severe)***	Life-threatening for the patient. Requires urgent intervention.
Grade **5** ***(Severe)***	Death related to the adverse event.

The analysis of the causality relationship between the adverse event and the drug under study will be carried out using the following qualitative analyses[xix]:

1. <u>Definitive</u>: An event that **1)** shows a reasonable chronological relationship; **2)** follows a known response to the drug under study; **3)** there is no reasonable explanation that it may be produced by

other factors such as the clinical condition of the individual or concomitant drugs administered; **4)** it disappears when its administration is stopped, and reappears when it is renewed.

2. Probable: An event that **1)** shows a reasonable chronological relationship after the administration of the drug; **2)** shows a known response pattern to the drug under study; **3)** there is no explanation due to other factors such as the clinical condition of the individual or concomitant drugs administered; **4)** it disappears when the administration is stopped, but it is not confirmed with its re-exposure.

3. Possible: An event that **1)** shows a reasonable chronological relationship; **2)** it may follow, or not, a known response pattern of the drug under study, but **3)** it may be produced by other factors such as the clinical condition of the individual or concomitant drugs administered.

4. Doubtful: the event is more probably related to other factors than to the drug involved.

No adverse events associated to the administration of Jusvinza by the intra-venous route have been described.

X. DATA COLLECTION AND HANDLING

10.1. Information recording form

The information of the protocol is collected in the following forms:

Form	The form is filled when	Information collected
Data Recording Form<br>CR of each patient	There is a clinical record, reviewed by the physician in charge.	Results of progress tests (hematological, biochemical and molecular tests) imaging, data on the administration of the product and adverse events.

The specialist and a capable and trained person will be responsible for filling all these forms by using the clinical records of the patients as the primary source of information.

The notes will be taken in black or blue ink (preferably black), and without crossing-out or erasing any information, or having illegible letters or words. When a correction is required, the incorrect value or information will be crossed-out with a single line and the correct result will be noted down; information is never erased.

10.2. Procedure for conserving the information

All the information derived from the protocol will be filed at the clinical site, including the clinical records, the DRF and electronic or printed documents. This information will be stored in locked metal archives, for which access is only granted to the specialists, monitors and the management committee of the protocol.

During the implementation of the protocol, the information related to the patients will be stored in duplicate. After it is submitted to the promoter center, a copy of it will be stored at the archive of the clinical site.

The DRF of all patients will be stored on a physical support (CD-R), in the passive archive of the Clinical Trials Department of the CIGB, for at least 15 years after the protocol is completed.

XI. STATISTICS

11.1 Number of patients planned

We consider as the number of patients, all those having information according to this study, at the Dr. Luis Díaz Soto Hospital and complying with the inclusion criteria.

11.2 Plan for the statistical analysis

11.2.1 Set of data analyzed

There are two groups:

"Treated with Jusvinza": defined as the patients that have been treated with Jusvinza, according to the the information in their clinical records.

"Not treated with Jusvinza": defined as the patients that do not have any information in their clinical records of having been treated with Jusvinza.

.

XII. ASSURANCE

CIGB ensures the necessary resources and the logistics that are required for the implementation of this study.

XIII. GENERAL SCHEDULE

Stage	Start	End
Coordination and preparation of the protocol (version 1.0)	Abril 2021	Abril 2021
Review and approval by the Biomedical Research Division (version 1.0)	May 5 2021	May 7 2021
Review and approval by the Ethics Committee for the Study (version 1.0)	May 14-20 2021	
Start of data collection	May 2021	September 2021
Processing and analysis of final results	July 2021	September 2021
Preparation of the final report	October 2021	November 2021
Submitting the first report on the evolution of critically ill patients		January 2022
Submitting the second report on the evolution of severely ill and moderately ill patients		March 2022

XIV. PRACTICAL CONSIDERATIONS

14.1. Distribution of the duties and responsibilities in the protocol.

14.1.1. Responsibilities of the promoter (CIGB)

a) Carry out the process of designing and the proposal of the study with all participants.

b) Designate the monitor in charge of the protocol as the representative of the interests of the promoter.

c) Request a report on the pertinence of the research to CECMED.

d) Carry out the statistical processing of the data.

e) Notify CECMED on the results of the protocol for its inclusion in the dossier for the registration of the product.

f) Store the primary information and all data for 15 years.

g) Register the clinical trial in the Registry for the Control of Clinical Trials (RCEC)

14.1.2. Monitors

a) Participate in the design and preparation of the protocol.

b) Notify CECMED on the start of the implementation of the protocol.

c) Ensure the processing of the data and the statistical analysis that will be carried out at the end.

d) Participate together with the specialists in the preparation of the final report, with the results of the protocol and of the articles that will be published (procedure 4.40.040.00 in force in the Clinical Research Division of the CIGB).

14.3.3. Specialists in charge, and their designated research team

a) Participate in the design and preparation of the protocol.

b) Ensure the compliance to Good Clinical Practices.

c) Use, as the source of primary information in the clinical records

d) Maintain the confidentiality of the information generated during the implementation of the protocol. The authorization of the promoter is required for the dissemination of results.

e) Write all reports required, and participate in the discussion and analyses of the results of the protocol.

14.4. Procedures for the flow of documents

The clinical records will be kept in the document warehouse of the Hospital Dr Luis Diaz Soto. Obtaining data from medical records for writing reports and scientific articles will be done with the authorization of the Dr Luis Diaz Soto Hospital management.

14.5. Considerations on the problems of confidentiality, dissemination of results and other legal issues

The medical specialists of the protocol, the promoter, monitors and auditors designated by the promoter, will guarantee that the personal data of the subjects included in the protocol will be handled in accordance with the stipulations established in Law 15/1999 for the protection of personal data and the norms for this. Moreover, the anonymity of the subjects included in the protocol will be maintained, as well as the protection of their identity. We will not give away any personal information on the subjects of the protocol, except for cases under the circumstances expressed by law.

The specialists, the promoter, monitors and auditors designated by the promoter are committed to handle the documentation, information, results and data related to the protocol, according to their confidential and secret nature. They are committed to oversee the restricted circulation of this information and are responsible that all persons that have access to it, according to regulations established in this section, fulfill this obligation.

The monitors and auditors designated by the promoter may have Access to the clinical information and documentation on the subjects included, so that they can verify the accuracy and reliability of the data, but they must not collect the personal identification data of the subjects. They must also facilitate to inspectors of the competent health authorities the access to the data.

The results of the protocol, as well all the papers and reports made, and all industrial property rights derived from it, are of the exclusive property of the promoter. The promoter is in charge of disseminating them at the end of the protocol, whether the results are negative or positive, in the public access media.

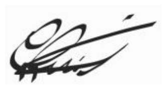

The dissemination of any information generated in this protocol is prohibited without the consent of the promoter. The publication of results in journals by medical specialists from the hospital, or scientific books, and oral presentations or posters in scientific congresses, workshops or meetings, must have first been accepted by the promoter center.

A copy of the manuscript or the original document must be submitted to the CIGB who must learn of its content and perform all timely verifications. CIGB, in a maximum term of 30 days, must report if it agrees or not with the content. If the CIGB considers that it is necessary to postpone the publication or presentation proposed by the specialist, this must be complied. If the CIGB considers that the specialist favors a certain interpretation of the data that could damage the rights of the CIGB, the scientific integrity must be guaranteed, and its interpretation must be ajusted so that it may comply with the criteria of the CIGB. If the parties do not reach an agreement, the specialist must include the interpretation of the CIGB in the publication or presentation. At the end of the above-mentioned term, if the promoter has not responded, it will be considered that the promoter agrees with the document and the physician will proceed to its publication or presentation.

The promoter must first request the corresponding authorization to the medical specialists to be able to use their names in scientific papers, or in any other means of dissemination for commercial or informational purposes.

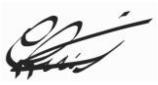

XVI. REFERENCES

[i] Xu Z, Shi L, Wang Y, et al. Pathological findings of COVID-19 associated with acute respiratory distress syndrome. Lancet Respir Med 2020; 8:420-422.

[ii] Zhou F, Yu T, Du R, et al. Clinical course and risk factors for mortality of adult inpatients with COVID-19 in Wuhan, China: a retrospective cohort study. Lancet 2020; 395: 1054-62.

[iii] Domínguez MC, Lorenzo N, Barberá A, Darrasse-Jeze G, Hernandez MV, Torres AM et al. An altered peptide ligand corresponding to a novel epitope from heat-shock protein 60 induces regulatory T cells and suppresses pathogenic response in an animal model of adjuvant induced arthritis. Autoimmunity 2011; 44 :471-82.

[iv] Barberá A, Lorenzo N, van Kooten P, et al. APL1, an altered peptide ligand derived from human heat-shock protein 60, increases the frequency of Tregs and its suppressive capacity against antigen responding effector CD4+T cells from rheumatoid arthritis patients. Cell Stress and Chaperones. 2016; 21:735–744.

[v] Lorenzo N, Altruda F, Silengo L and Dominguez MC. APL-1, an altered peptide ligand derived from heat-shock protein, alone or combined with methotrexate attenuates murine collagen induced arthritis. ClinExpMed 2017; 17:209–216.

[vi] Domínguez MC, Cabrales A, Lorenzo N, Padrón G and Gonzalez LJ. Biodistribution and pharmacokinetic profiles of an Altered Peptide Ligand derived from Heat-shock proteins 60 in Lewis rats. Cell Stress and Chaperones.2020;25(1):133-140
[vii] Dinorah Prada, Jorge Gómez, Norailys Lorenzo, Oreste Corrales, et al. Phase I Clinical Trial with a Novel Altered Peptide Ligand Derived from Human Heat-Shock Protein 60 for Treatment of Rheumatoid Arthritis: Safety, Pharmacokinetics and Preliminary Therapeutic Effects. Journal of Clinical Trials 2018; 8:2167-0870

[viii] Cabrales-Rico, A., Ramos, Y., Besada, V., Del Carmen, D. M., Lorenzo, N. et al (2017): Development and validation of a bioanalytical method based on LC-MS/MS analysis for the quantitation of CIGB-814 peptide in plasma from Rheumatoid Arthritis patients. J Pharm.Biomed.Anal. 143: 130-140.

[ix]Oreste Corrales, Laura Hernández, Dinorah Prada, et al. CIGB-814, an altered peptide ligand derived from human heat-shock protein 60, decreases anti-cyclic citrullinated peptides antibodies in patients with rheumatoid arthritis. Clinical Rheumatology 2019; 38:955–960.

[x] Venegas-Rodriguez R et al (2020). CIGB-258 Immunomodulatory Peptide: Compassionate Use for Critical and Severe COVID-19 Patients. Austin J Pharmacol Ther 8(1).1119.

[xi] Enrique Galbán-García and Pedro Más-Bermejo. (2020). COVID-19 in Cuba: Assessing the National Response. MEDICC Review, Vol 22, No 4

[xii]Ministry of Public Health of Cuba. Ministerio de Salud Pública. Cuba: Parte de cierre del día 31 de marzo a las 12 de la noche [Internet] [cited 2021 Mar 31]. Available from: https://salud.msp.gob.cu/parte-de-cierre-del-dia-31-de-marzo-a-las-12-de-la-noche/

[xiii] Liu J, Liu Y, Xiang P, Pu L, Xiong H, Li C (2020) Neutrophil-to-lymphocyte ratio predicts critical illness patients with 2019 coronavirus disease in the early stage. J Transl Med 18(1):206

[xiv] Chen L, Long X, Xu Q, Tan J, Wang G, Cao Y (2020) Elevated serum levels of S100A8/A9 and HMGB1 at hospital admission are correlated with inferior clinical outcomes in COVID-19 patients. Cellular and Molecular Immunology 17:992–994.

[xv] Zheng M et al (2020) Functional exhaustion of antiviral lymphocytes in COVID-19 patients. Cell Mol Immunol 17:533-535

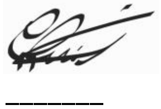

[xvi] Zheng HY et al (2020) Elevated exhaustion levels and reduced functional diversity of T cells in peripheral blood may predict severe progression in COVID-19 patients. Cell. Mol. Immunol. 17, 541–543.

[xvii] Hernandez-Cedeño M et al (2021). CIGB-258, a peptide derived from human heat-shock protein 60, decreases hyperinflammation in COVID-19 patients. Cell Stress and Chaperones. DOI: 10.1007/s12192-021-01197-2

xviii. U.S. Department of Health and Human Services, National Institutes of Health, National Cancer Institute. Common Terminology Criteria for Adverse Events, Version 5.0, November 27, 2017. Available in http://ctep.cancer.gov

xix. Naranjo CA, Shear NH, Busto U. Adverse drug reactions. In: Kalant H and Roschlau WHE. Principles of medical pharmacology. 6th ed. New York: Oxford University Press, 1998:791-800.
