# Supplementary material for: Jusvinza, an anti-inflammatory drug derived from the human heat-shock protein 60, for critically ill COVID-19 patients. An observational study
Source: PLoS One. 2023 Feb 2;18(2):e0281111. doi: 10.1371/journal.pone.0281111 (PMC9894446; doi:10.1371/journal.pone.0281111)

CIGB CENTRO DE INGENIERÍA GENÉTICA Y BIOTECNOLOGÍA

BioCubaFarma GRUPO DE LAS INDUSTRIAS BIOTECNOLÓGICA Y FARMACÉUTICA

La información contenida en este documento es propiedad del CIGB. La misma no puede ser reproducida, publicada o revelada a otros sin la autorización explícita del promotor.

PROTOCOLO DEL ESTUDIO RETROSPECTIVO

ESTUDIO "VALENTÍA"

"Estudio observación retrospectivo de la administración de Jusvinza según el protocolo de actuación de CUBA, para el manejo de la COVID-19 en el Hospital Dr Luis Díaz Soto"

Código del estudio: INV/CIGB-258/003/21

Versión: 1.0

La Habana, 4 de mayo de 2021

INDICE

RESUMEN *4*

LISTA DE ABREVIATURAS UTILIZADAS Y DEFINICIÓN DE TÉRMINOS **6**

I. INFORMACION GENERAL **8**

1.1. Título del ensayo clínico: “Estudio retrospectivo de la administración de Jusvinza según el protocolo de actuación de CUBA para el manejo de la COVID-19, en el Hospital Militar Central Dr. Luis Díaz Soto” 8

1.2. Código: INV/CIGB-258/003/21 8

1.3. Patrocinadores 8

1.4. Institución participante 8

1.5 Responsables de la organización e implementación del estudio, recogida y procesamiento de la información – CIGB, La Habana 8

II. INTRODUCCION **10**

2.1. Datos fundamentales sobre el problema en cuestión y su contexto. 10

III. OBJETIVOS **12**

3.1. Objetivo General *12*
3.2. Objetivos específicos *12*
3.3 Hipótesis *13*

IV. DEONTOLOGIA MÉDICA **13**

4.1. Comité de Ética y Revisión (CER) / Comité de Ética en la Investigación Científica (CEIC) 13

4.2. Aspectos éticos en la conducción del ensayo 13

4.3. Instrucciones para la obtención del consentimiento informado 14

4.4. Responsabilidades éticas de todos los participantes en la investigación 14

V. CONCEPCIÓN GENERAL **16**

5.1 Diseño del ensayo 16

5.2 Identificación de los pacientes 16

5.3 Factores que pueden introducirse para reducir sesgos 16

VI. SELECCIÓN DE LOS SUJETOS **16**

6.1. Universo de pacientes 16

VII. TRATAMIENTOS A INVESTIGAR **17**

VIII. VARIABLES DE EVALUACIÓN **17**

8.1. Variables del protocolo 17

8.1.1. Variables principales *18*
8.1.2. Variables secundarias *18*
8.1.3. Variables de control *18*

8.2. Criterios para el éxito o fracaso individual y de la terapéutica 19

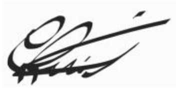

IX. EVENTOS ADVERSOS **19**

9.1 Reacciones adversas que pueden presentarse y métodos para registrarlos 19

X. RECOLECCION Y MANIPULACIÓN DE LOS DATOS **20**

10.1. Modelo de recolección de la información 20

10.2. Procedimientos para conservar la información 21

XI. ESTADÍSTICA **21**

11.1 Número de pacientes planeados 21

11.2 Plan de análisis estadístico 21

11.2.1 Conjunto de datos analizados *21*

XII. ASEGURAMIENTO **22**

XIII. CALENDARIO GENERAL **23**

Etapa 23

XIV. CONSIDERACIONES PRÁCTICAS **24**

14.1. Reparto de los deberes y responsabilidades en el protocolo. 24

14.1.1. Responsabilidades del promotor (CIGB) *24*

14.1.2. Monitores *24*

14.3.3. Especialistas responsables y su equipo de investigación designado *24*

14.4. Procedimientos para el flujo de la documentación 25

14.5. Consideraciones sobre los problemas de confidencialidad, divulgación de los resultados y otros aspectos legales 25

XVI. REFERENCIAS **27**

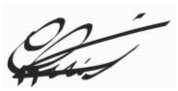

RESUMEN:

Titulo	"Estudio retrospectivo de la administración de Jusvinza según el protocolo de actuación de CUBA para el manejo de la COVID-19, en el Hospital Dr Luis Diaz Soto"
Población de pacientes	Pacientes ≥ 19 años de edad positivos al SARS-CoV-2 por RT-PCR, clasificados como pacientes de moderados, graves y críticos, según protocolo de actuación de CUBA para el manejo de la COVID-19
Racionalidad del estudio	Los pacientes con COVID-19 que transitan hacia las fase graves d ela enfermedad de la enfermedad presentan una marcada hiperinflamación, que los puede llevar al colapso cardiovascular y al fallo múltiple de órganos y conducir a la muerte. En este escenario, se recomienda la identificación y tratamiento de la hiperinflamación, con el objetivo de reducir la mortalidad. En Cuba se usa el medicamento Jusvinza para el tratamiento de los pacientes moderados, graves y críticos con COVID-19. Jusvinza es un péptido inmunomodulador con propiedades anti-inflamatorias.
Objetivos del estudio	**General:** Observar la efectividad en la reducción de la hiperinflamación y la seguridad de la administración de Jusvinza, en pacientes con COVID-19 clasificados como moderados, graves y críticos.<br>**Objetivos específicos**<br>• Describir la evolución clínica de los pacientes de alto riesgo a grave o crítico o fallecido<br>• Describir la evolución de los marcadores inflamatorios de los pacientes incluidos.<br>• Describir la evolución radiológica de los pacientes incluidos.<br>• Describir los eventos adversos.
Hipótesis	Al considerar que en el estudio extensivo del uso de Jusvinza en el país, el 85 % de los pacientes graves se recuperaron; esperamos que el 90% de los pacientes de alto riego tratados con Jusvinza no transiten a las fases graves de la enfermedad y se recuperen.
Variables principal	Porcentaje de pacientes de alto riego, graves y críticos recuperados después del tratamiento con Jusvinza.
Variables secundarias	1. Describir la evolución clínica de los pacientes.<br>2. Describir la evolución radiológica.<br>3. Cuantificar los biomarcadores enflamatorios séricos (NLR, proteína C-reactiva, eritrosedimentación, Ferritina, LDH, dímero-D, CPK, ASAT, ALAT).<br>4. Cuantificar las citocinas: IL-6, TNFα e IL-10.<br>5. Cuantificar el porcentaje de linfocitos CD4, CD8 y Treg.<br>6. Ocurrencia y caracterización de eventos adversos.

Diseño del estudio	Estudio retrospectivo, observacional, monocéntrico.<br>Se recogerá la información retrospectivamente de cada paciente utilizando las historias clínicas, como fuente de información primaria así como los resultados de laboratorio clínico e imágenes radiológicas conservados en el programa GALEN de la institución participante.
Número de pacientes	Se incluirán todos los pacientes positivos al SARS-CoV-2 por RT-PCR con información disponible, clasificados como de alto riego, greves y criticos que se les haya indicado Jusvinza.
Frecuencia y duración de la terapia	Según las indicaciones del Protocolo de Actuación
Medicamento en estudio	Medicamentos: Jusvinza (2,5 mg o 1,25 mg), administración intravenosa.
Evaluación de la seguridad	• Síntomas<br>• Signos vitales<br>• Resultados analíticos<br>• Resultados de imágenes

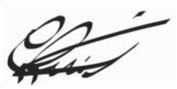

LISTA DE ABREVIATURAS UTILIZADAS Y DEFINICIÓN DE TÉRMINOS

BPC: Buenas Prácticas Clínicas: Un estándar para el diseño, conducción, realización, monitoreo, auditoría, registro, análisis y reporte de estudios clínicos que proporciona una garantía de que los datos y los resultados reportados son creíbles y precisos y de que están protegidos los derechos, integridad y confidencialidad de los sujetos del estudio. (Tomado de la regulación 21 – 08: requisitos para la autorización y modificación de ensayos clínicos. CECMED)

CECMED: Centro para el Control Estatal de Medicamentos, Equipos y Dispositivos Médicos.

CEIC / CER: Comité de Ética de la Investigación Científica / Comité de Ética y Revisión.

CIGB: Centro de Ingeniería Genética y Biotecnología.

CRD: Cuaderno de Recogida de Datos.

Evento Adverso: Es cualquier incidente médico desfavorable que ocurre en un sujeto que participa en un ensayo clínico ante la administración de un producto farmacéutico. Ese incidente no guarda necesariamente una relación causal con el tratamiento. Un evento adverso puede, por tanto, ser un signo no favorable o inesperado (incluido un hallazgo anormal de laboratorio, por ejemplo), síntoma o enfermedad temporalmente asociada con el uso de un producto medicinal. (Tomado de las Directrices sobre Buenas Prácticas Clínicas, CECMED, 2000)

Evento adverso inesperado: Cualquier evento adverso en el que la especificidad o gravedad no es consistente con la información de riesgo descrita en el protocolo o en el manual del investigador (si existe alguno). Se refiere, además, a un evento adverso que no ha sido observado previamente.

Jusvinza: péptido inmunomodulador CIGB-258.

MINSAP: Ministerio de Salud Pública.

PCR-RT: Reacción en cadena de la polimerasa en tiempo real.

Reacción adversa: Se refiere a un evento adverso que se considera relacionado de forma causal con el producto de la investigación; incluye sobredosis e interacciones con otros medicamentos. Toda respuesta nociva no deseada producida por un producto farmacéutico a cualquier dosis, debe ser considerada reacción adversa a drogas. Una definición bien aceptada de una reacción adversa a drogas es hallada en el Reporte Técnico de la Organización Mundial de la Salud (Serie No. 850, 1995) que

plantea: “una reacción adversa es una respuesta nociva y no intencionada frente a un producto farmacéutico que ocurre a dosis normalmente usadas en el hombre para profilaxis, diagnóstico, terapia, o para la modificación de una función fisiológica. En ensayos clínicos, los daños causados por sobredosis, abuso o dependencia, e interacciones con otros productos, deben considerarse reacciones adversas”.

SARS-CoV: Coronavirus del síndrome respiratorio agudo severo.

I. INFORMACION GENERAL

1.1. Título del ensayo clínico: "Estudio retrospectivo de la administración de Jusvinza según el protocolo de actuación de CUBA para el manejo de la COVID-19, en el Hospital Militar Central Dr. Luis Díaz Soto"

1.2. Código: INV/CIGB-258/003/21

1.3. Patrocinadores

❖ Centro de Ingeniería Genética y Biotecnología (CIGB).
Grupo de las Industrias Biotecnológicas y Farmacéuticas BioCubaFarma.
Ave. 31 e/ 158 y 190, Cubanacán, Playa, La Habana, Apartado 6162, C.P. 11600, Cuba.
☎ (53-7)-2716022; Fax (53-7)-2716070 / 2736008; Web: http://www.cigb.edu.cu

1.4. Institución participante

o Hospital "Dr. Luis Díaz Soto", La Habana.

1.5 Responsables de la organización e implementación del estudio, recogida y procesamiento de la información – CIGB, La Habana.

- Dr.C. María del Carmen Domínguez Horta: Doctor en Ciencias Biológicas; Investigador y Profesor Titular.
- MSc. Mabel Hernández Cedeño : Investigadora de la Direccion de Investigaciones Biomédicas
- Lic. Anabel Sierra: Investigadora de la Direccion de Investigaciones Biomédicas
- Dra Deylis Chacón . Jefa de la Unidad de Urgencias Hospital Militar Central "Dr. Luis Díaz Soto"
- My. Dra Leticia del Rosario. Segunda Jefade la Unidad de Urgencias Hospital Militar Central "Dr. Luis Díaz Soto

1.6. Monitores – Dirección de Investigaciones Clínicas del CIGB, La Habana

- MSc. Mabel Hernández Cedeño: Investigadora de la Direccion de Investigaciones Biomédicas
- Lic. Anabel Sierra: Investigadora de la Direccion de Investigaciones Biomédicas

1.7. Responsables del manejo de datos y análisis estadístico – CIGB, La Habana.

- MSc. Mabel Hernández Cedeño : Investigadora de la Direccion de Investigaciones Biomédicas
- Lic. Anabel Sierra: Investigadora de la Direccion de Investigaciones Biomédicas

1.8. Asesor – CIGB, La Habana

- Dr.C. Gerardo Enrique Guillén Nieto: Doctor en Ciencias Biológicas; Licenciado y Máster en Ciencias Químicas; Investigador, Profesor y Académico Titular. Director de Investigaciones Biomédicas; Secretario del Consejo Científico del CIGB, La Habana.

1.9. Comité de Ética y Revisión

El estudio biomédico se realizará retrospectivamente teniendo en cuenta los datos disponibles en las historias clínicas de los pacientes tratados con Jusvinza.

Se utilizará como fuente de información para la recogida de datos, las Historias Clínicas y los resultados de laboratorio clínico y de imágenes disponibles de cada paciente en el programa GALEM. Se obtendrá la aprobación del Comité de ética para acceder a la información de los pacientes.

El protocolo será presentado en el registro público de ensayos clínicos del CENCEC.

II. INTRODUCCION

2.1. Datos fundamentales sobre el problema en cuestión y su contexto.

Los pacientes con COVID-19 que transitan hacia estadios graves y críticos de laCOVID-19 presentan una marcada hiperinflamación. Esta etapa de la enfermedad esta mediada por altas concentraciones de citocinas proinflamatorias, evento biológico conocido como "tormenta de citocinas". A medida que la hiperinflamacion progresa estos pacientes pueden llegar al colapso cardiovascular y al fallo múltiple de órganos que los conduce a la muerte (1,2)

En este escenario, se recomienda la identificación y tratamiento de la hiperinflamación, con el objetivo de reducir la mortalidad. En Cuba se usa el medicamento Jusvinza para el tratamiento de los pacientes moderados, graves y críticos con COVID-19. Jusvinza es un péptido inmunomodulador con propiedades antiinflamatorias. Este péptido fue desarrollado en el CIGB para el tratamiento de las enfermedades autoinmunes, específicamente para la artritis reumatoide (AR) (3).

Se ha comprobado que este péptido incrementa la frecuencia de las células T reguladoras (Treg) con fenotipo CD4+CD25^{high}Foxp3+ en ensayos *ex vivo* con células mononucleares de sangre periférica (PBMC, del inglés *Peripheral Blood Mononuclear Cells*) de pacientes con AR pero no en donantes sanos; estas células tienen actividad supresora (4). Asimismo, al administrarse por vía subcutánea, el péptido induce un aumento significativo de la población de células Treg con fenotipo CD4+Foxp3+ en los nódulos linfáticos en contacto con el sitio de administración y en el bazo de ratones BALB/c. Además, este péptido inhibe eficientemente la inflamación en modelos de artritis (5).

Por otra parte, los estudios de farmacocinética y biodistribución a través de tres vías: intravenosa, intradérmica y subcutánea, demostraron que el péptido tiene una amplia distribución hacia diferentes órganos: tracto gastrointestinal, hígado, pulmones, etc. La máxima concentración en sangre la alcanza a la media hora y su aclaramiento ocurre en 6 horas aproximadamente (6). Los estudios de toxicología en tres especies animales demostraron que el péptido tiene un buen perfil de seguridad.

Estos resultados en la etapa preclínica de investigación, permitieron que la Autoridad Reguladora de Medicamentos, Equipos y Dispositivos Médicos de la República de Cuba (CECMED) concediera la autorización para la evaluación del péptido en un estudio clínico fase I en pacientes con AR

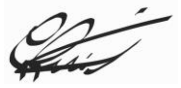

(RPCEC00000238). En este ensayo clínico fase I se demostró la seguridad, perfil fármacocinético y se obtuvieron evidencias preliminares de efecto terapéutico (7,8, 9). Actualmente están en procesamiento los resultados de un ensayo clínico fase II en pacientes con AR (RPCEC00000230), para evaluar la eficacia y seguridad de este candidato terapéutico. Este ensayo es controlado, aleatorizado y a triple ciega, se incluyeron 187 pacientes. Se ha observado un buen perfil de seguridad.

El conjunto de estos resultados avalaron que el CECMED concediera el permiso para el uso compasivo de Jusvinza en el tratamiento de pacientes críticos con COVID-19 (RPCEC00000313). Se inició el protocolo de acción con Jusvinza el 31 de marzo del 2020, en la unidad de cuidados intensivos (UCI) del Hospital Militar Luís Díaz Soto, de la Habana. Los especialistas en sus reportes médicos describieron que a partir de las 48 horas de tratamiento con Jusvinza, los pacientes comenzaron a presentar mejorías clínicas, gasométricas y radiológicas. Estos 12 pacientes fueron extubados y se recuperaron, continúan bajo estricta vigilancia en sus áreas de salud. Los parámetros de laboratorio indicaron que los pacientes antes de iniciar el tratamiento presentaron linfopenia y una tendencia a la neutrofilia. Sin embargo, durante el trascurso del tratamiento los niveles de linfocitos y neutrófilos alcanzaron sus valores normales. De igual forma, los marcadores asociados a la hiperinflamación: proteína C reactiva , ferritina, lactado deshirogenasa, fibrinógenos, creatinina y las transaminasas fueron normalizándose durante el tratamiento. La concentración de tres citocinas involucradas en la tormenta de citocinas: la IL-6, el TNF-α, IL-1 e IL-10, se fueron normalizando en el trascurso del tratamiento (10). El conjunto de estos resultados y el perfil de seguridad del Jusvinza permitieron que se autorizara su uso para el tratamiento de los pacientes graves con COVID-19; y se aprobará la inclusión de Jusvinza en el protocolo nacional de tratamiento para los pacientes graves y críticos.

La incorporación de Jusvinza al protocolo nacional cubano aprobado por el Ministerio de Salud Pública, para el tratamiento de pacientes graves y críticos con COVID-19, tuvo lugar el 27 de abril del 2020, así como la extensión de su uso a todos los hospitales cubanos que atienden COVID-19. El uso de Jusvinza ha impactado en disminuir la tasa de letalidad en Cuba; la tasa de letalidad en Cuba en abril de 2020 fue de 4,16 (11) y en marzo del 2021 fue de 0,46 (12).

El tratamiento con Jusvinza ha sido seguro para los pacientes. Los estudios moleculares realizados en Cuba, durante la fase de extensión del uso de Jusvinza a todos los hospitales que atienden COVID-19, han corroborado los resultados obtenidos durante el estudio de uso compasivo; y han demostrado que la

terapia con Jusvinza es capaz de reducir la hiperinflamación en los pacientes con COVID-19. La administración de Jusvinza en los pacientes graves evita que progresen al estadio crítico de la enfermedad. La efectividad del tratamiento en los pacientes graves supera el 86%. Los niveles de la Proteína C Reactiva (PCR) disminuyen significativamente en los pacientes, a partir de las 72 horas de tratamiento. Se ha podido confirmar una correlación inversamente significativa entre la mejoría de los parámetros ventilatorios y la PCR en los pacientes críticos con ventilación mecánica invasiva. Otros biomarcadores de la inflamación como: ferritina, lactado deshirogenasa, creatinina y las transaminasas disminuyeron significativamente. Diversos trabajos describen que estos pacientes se caracterizan por presentar neutrofilia y linfopenia; con un cociente neutrófilos/linfocitos (NLR) superior a 5,1 (13). La terapia con Jusvinza redujo el NLR a valores normales. Además, redujo los niveles de la Calprotectina, una proteína secretada por monocitos y neutrófilos durante los procesos inflamatorios (14). La reducción de la Calprotectina se correlacionó significativamente con la disminución de los neutrófilos. Además, se ha documentado que la desregulación de la respuesta inmunitaria en los pacientes COVID-19, se asocia con un aumento de la Granzima B y la Perforina y una disminución del porcentaje de las Treg (15, 16). Los resultados en nuestros pacientes indicaron una disminución significativa de Granzima B y Perforina, a las 96 horas de tratamiento, coincidiendo con la disminución de las interleucinas IL-6, IL-10 y el TNF-α. El porcentaje de las Treg aumentó, después de 48 horas de tratamiento, en los pacientes graves estudiados (17). El aumento de las Treg en los pacientes COVID-19 es muy congruente con el mecanismo de acción de Jusvinza en los pacientes con AR (3,4).

Los resultados en su conjunto posibilitaron concluir que la terapia con Jusvinza disminuye la hiperinflamación que caracteriza a los pacientes con COVID-19.

III. OBJETIVOS

3.1. Objetivo General

- Observar la efectividad en la reducción de la hiperinflamación y la seguridad de la administración de Jusvinza, en pacientes con COVID-19 clasificados como moderados, graves y críticos.

3.2. Objetivos específicos

1. Describir la evolución clínica de los pacientes de alto riesgo a grave o crítico o fallecido
2. Describir la evolución de los marcadores inflamatorios de los pacientes.
3. Describir la evolución radiológica de los pacientes.
4. Describir los eventos adversos.

3.3 Hipótesis

Al considerar que en el estudio extensivo del uso de Jusvinza en el país, el 85 % de los pacientes graves se recuperaron; esperamos que el 90% de los pacientes tratados con Jusvinza no transiten a las fases graves de la enfermedad y se recuperen.

IV. DEONTOLOGIA MÉDICA

4.1. Comité de Ética y Revisión (CER) / Comité de Ética en la Investigación Científica (CEIC)

Para iniciar la ejecución del protocolo de ensayo clínico en la unidad asistencial se obtendrá el dictamen del Comité de Ética Institucional quien certificara después de realizada la valoración y análisis correspondiente que el documento (versión 1.0):

- ✓ Se ajusta a la Declaración de Helsinki (Principios éticos para las investigaciones médicas en seres humanos, adoptada por la Asamblea Médica Mundial, Fortaleza, Brasil, 2013).
- ✓ Se ajusta a las normas y criterios éticos establecidos en los códigos nacionales e internacionales de ética y regulaciones legales vigentes en Cuba (Directrices de Buenas Prácticas Clínicas, CECMED 2000, Cuba; Guía de Buenas Prácticas Clínicas de la Conferencia Internacional de Armonización – ICH E-6).
- ✓ Recoge la forma de protección de los derechos y bienestar de los pacientes involucrados.
- ✓ Describe satisfactoriamente los criterios de selección de pacientes.

4.2. Aspectos éticos en la conducción del ensayo

El estudio retrospectivo está debidamente avalado desde el punto de vista ético por las siguientes razones:

a. Es aprobado por el CER/CEIC. De igual forma, como una contribución a la transparencia de la investigación, el protocolo se publicará en el Registro Público Cubano de Ensayos Clínicos.
b. No se realizará intervención clínica sobre los pacientes, se observará la evolución de estos, una vez cuando son tratados con el medicamento Jusvinza. Existen evidencias del efecto en la reducción de la inflamación de los pacientes con COVID-19 tratados con Jusvinza. El tratamiento con Jusvinza ha sido seguro para los pacientes. Los estudios moleculares realizados en Cuba, durante la fase de extensión del uso de Jusvinza a todos los hospitales que atienden COVID-19, han confirmado los resultados obtenidos durante el estudio de uso compasivo; y han demostrado que la terapia con Jusvinza es capaz de reducir la inflamación en los pacientes con COVID-19. La administración de Jusvinza en los pacientes graves evita que progresen al estadio crítico de la enfermedad. La efectividad del tratamiento en los pacientes graves supera el 85%.
c. Los resultados que se obtengan del procesamiento de la información que se recoja de los pacientes tratados con Jusvinza generará un beneficio para los pacientes de COVID-19 que vayan a ser objeto del tratamiento en el futuro.
d. Se respetará la integridad de los participantes en la investigación, asegurando la confidencialidad de todos los datos de los pacientes.

4.3. Instrucciones para la obtención del consentimiento informado

No procede.

4.4. Responsabilidades éticas de todos los participantes en la investigación

Investigador: Brindar acceso a la información del paciente registrada en las Historias Clínicas

Institución: Asegurar el mantenimiento y utilización adecuada de las facilidades por parte del investigador y someter el protocolo a la aprobación por el Comité de Revisión y Ética (facilitado por el investigador responsable).

Equipo de investigación: Garantizar el cumplimiento de las responsabilidades asignadas.

Promotor: Garantizar la recolección de la información de forma fidedigna, respetando la confidencialidad de los datos de los pacientes.

Comité de Revisión y Ética: Revisar y aprobar el protocolo del ensayo asegurando la protección de los derechos de los pacientes involucrados en el estudio y proporcionar una garantía pública de esa protección. Verificar el progreso del estudio y la adherencia de los investigadores al protocolo.

V. CONCEPCIÓN GENERAL

5.1 Diseño del ensayo

Se realizará un estudio retrospectivo, observacional, monocéntrico, con el objetivo primario de evaluar el efecto en la reducción de la inflamación y la seguridad de la administración intravenosa de Jusvinza.

Se incluirán todos los pacientes, de los que se tenga información disponible y que se le haya indicado tratamiento con Jusvinza, según el protocolo de actuación, en el periodo comprendido entre el 21 de marzo y el 29 de julio del 2021.

5.2 Identificación de los pacientes

Cada paciente quedará identificado por un código que indica el "número de inclusión consecutivo", seguido de la siglas correspondientes al nombre/o nombres y apellidos. Esta identificación deberá figurar en los documentos correspondientes a cada paciente. *Ejemplo,* para el 1er paciente incluido en el estudio, el código de identificación será: 001-XYZM, donde X es la inicial del primer nombre, Y es la inicial del segundo nombre (si lo tuviera), Z la inicial del primer apellido y M la del segundo apellido.

5.3 Factores que pueden introducirse para reducir sesgos

- Previo a la confección de este protocolo se efectuó un intercambio con los especialistas vinculados al manejo del uso de Jusvinza en pacientes con COVID-19 en el Hospital Militar Central Dr Luis Diaz Soto; y los investigadores de la Direccion de Investigaciones Clínicas y la Dirección de Investigaciones Biomédicas del CIGB, donde se presentaron y discutieron las experiencias clínicas del uso de Jusvinza en los pacientes con COVID-19, que ha permitido avalar la investigación y permitieron la definición del presente diseño experimental.
- El análisis, discusión y dominio del protocolo, favorecerá la adherencia y cumplimiento de las BPC por parte de todos los investigadores.

VI. SELECCIÓN DE LOS SUJETOS

6.1. Universo de pacientes

El universo estará constituido por aquellos pacientes adultos, residentes permanentes en Cuba (con plenos derechos constitucionales), positivos al SARS-CoV-2 por RT-qPCR ingresado en el Hospital Dr

Luis Díaz Soto, que se les haya indicado tratamiento con Jusvinza, según el protocolo de actuación, en el periodo comprendido entre el 21 de marzo y el 29 de julio del 2021.

6.2. Criterios de inclusión

1) Pacientes con edad ≥ 19 años.

2) Pacientes positivos a SARS-CoV-2 por test confirmatorio de RT-qPCR moderados, graves, críticos o fallecidos y que se les haya indicado tratamiento con Jusvinza, en el periodo comprendido entre el 21 de marzo y el 29 de julio del 2021.

3) Pacientes que no fallezcan en las primeras 48 horas de estar hospitalizados.

4) Pacientes que no estén incluidos en otros ensayos clínicos.

VII. TRATAMIENTOS A INVESTIGAR

- **JUSVINZA®**

Presentado como un polvo liofilizado estéril que contiene 2,5 mg o 1,25 mg, en frascos de cristal 2R.

Ingredientes activos: péptido inmunomodulador CIGB-258, obtenido por síntesis química en el Centro de Ingeniería Genética y Biotecnología (CIGB, La Habana, Cuba).

La composición de cada bulbo se presenta en la siguiente tabla:

Componente	Cantidad	Función	Norma de calidad
Péptido CIGB-258	2,5 mg o 1,25mg	Inmunomodulador	NP 4380C Inyectable según fabricante
Sacarosa	20,0 mg	Estabilizante	NP 252 / USP
Ácido acético 50 mM, pH 4	3,0 mg	Ácido constituyente del tampón	NP 020 / USP

Acción: Efecto anti-inflamatorio e inmunomodulador.

VIII. VARIABLES DE EVALUACIÓN

8.1. Variables del protocolo

8.1.1. Variables principales

❖ **<u>Evaluación del efecto anti-inflamatorio</u>:**

- ✓ Se calculará el porcentaje de pacientes de alto riego recuperados después de siete días de tratamiento con Jusvinza.
- ✓ Se calculará el porcentaje de pacientes de graves recuperados después de diez días de tratamiento con Jusvinza.
- ✓ Se calculará el porcentaje de pacientes de críticos recuperados después de quince días de tratamiento con Jusvinza.

8.1.2. Variables secundarias

1. Describir la evolución clínica de los pacientes.
2. Describir la evolución radiológica.
3. Cuantificar los biomarcadores enflamatorios séricos (NLR, proteína C-reactiva, eritrosedimentación, Ferritina, LDH, dímero-D, CPK, ASAT, ALAT).
4. Cuantificar las citocinas: IL-6, TNFα e IL-10.
5. Cuantificar el porcentaje de linfocitos CD4, CD8 y Treg.
6. Describir los eventos adversos.

8.1.3. Variables de control

Se tendrán en cuenta por su posible efecto sobre la respuesta terapéutica:

a. Adherencia del paciente al tratamiento.
b. Antecedentes patológicos personales.
c. Edad (años)
d. Sexo (masculino / femenino).
e. Color de la piel (blanco, no blanco).
f. Sintomatología (sintomático, asintomático, pre-sintomático)
g. Tiempo entre el diagnóstico positivo de SARS-CoV-2 y comienzo de los primeros síntomas o

contacto.

h. Tratamientos anteriores y concomitantes al producto de investigación (antivirales, enfermedades de base)

i. Índice de masa corporal.

j. Hábitos tóxicos (tabaquismo, consumo de alcohol).

8.2. Criterios para el éxito o fracaso individual y de la terapéutica

👍 Se considerará **éxito individual** si el paciente se recupera, si no ocurren eventos adversos graves con causalidad demostrada.

👍 Se considerará **éxito de la terapéutica** si se demuestra la hipótesis planteada: el 90% de los pacientes de alto riego tratados con Jusvinza no transiten a las fases graves de la enfermedad y se recuperen.

👎 Se considerará **fracaso individual** progreso a la severidad o muerte en cualquier paciente y la interrupción definitiva del tratamiento por aparición de eventos adversos graves, con relación de causalidad atribuible al producto de investigación.

👎 Se considerará **fracaso de la terapéutica**: el tránsito de los pacientes hacia estadios graves de la enfermedad.

IX. EVENTOS ADVERSOS

9.1 Reacciones adversas que pueden presentarse y métodos para registrarlos

Los eventos adversos se clasificarán atendiendo al tipo, duración, intensidad, relación de causalidad, conducta seguida y resultado, en los siguientes grados[18]:

Grado **1** ***(Leve)***	Asintomático o síntomas de intensidad leve. Solo observación clínica o diagnóstica. No requieren tratamiento.
Grado **2** ***(Moderado)***	Requiere una intervención mínima, local o no invasiva.
Grado **3** ***(Grave)***	No compromete, de manera inmediata, la vida del paciente. Requiere hospitalización (o esta se prolonga). Incapacitante.
Grado **4** ***(Grave)***	Pone en peligro la vida del paciente. Requiere intervención urgente.
Grado **5** ***(Grave)***	Muerte relacionada al evento adverso.

El análisis de la relación de causalidad entre el evento adverso y el fármaco en estudio, se realizará empleando el siguiente análisis cualitativo[19]:

1. Definitiva: Un evento que **1)** muestra una relación temporal razonable; **2)** sigue una respuesta conocida a la droga en ensayo; **3)** no existe una explicación razonable de que sea producido por otros factores como el estado clínico del individuo o fármacos concomitantes administrados; **4)** desaparece cuando se detiene su administración y reaparece cuando se reinicia la exposición.
2. Probable: Un evento que **1)** muestra una relación temporal razonable después de la administración del medicamento; **2)** muestra un patrón de respuesta conocido a la droga en ensayo; **3)** no se puede explicar por otros factores tales como el estado clínico del individuo o fármacos concomitantes administrados; **4)** desaparece cuando se detiene su administración, pero no se confirma con la re-exposición.
3. Posible: Un evento que **1)** muestra una relación temporal razonable; **2)** puede o no seguir un patrón de respuesta conocido al fármaco en ensayo; pero que **3)** puede estar producido por otros factores tales como el estado clínico del individuo o fármacos concomitantes administrados.
4. Dudoso: El evento está más probablemente relacionado a otros factores que con el medicamento implicado.

No se han descrito eventos adversos asociados a la administración de Jusvinza por vía intrevenosa.

X. RECOLECCION Y MANIPULACIÓN DE LOS DATOS

10.1. Modelo de recolección de la información

La información del protocolo será recogida en los modelos siguientes:

Modelo	Momento en que se llena	Información que recoge
Datos recogidos de la Historia clínica de los pacientes	Cuando de disponga de la historia clínica, revisada por el medico a cargo.	Resultados de pruebas evolutivas (hematológicas, bioquímicas y moleculares) imágenes, dato de administración del producto, y eventos adversos.

El especialista y un personal capacitado y previamente entrenado será el responsable del llenado de todos estos modelos tomando como fuente primaria de la información de las historias clínicas de los pacientes.

Las anotaciones se realizarán con tinta negra o azul (preferentemente la primera) y no se producirán tachaduras o borrones, ni letras o palabras ilegibles. En caso de necesidad de realizar alguna corrección se tachará con una sola línea el valor o dato incorrecto y se anotará el resultado correcto, nunca se borrará.

10.2. Procedimientos para conservar la información

Toda información derivada del protocolo deberá archivarse en el sitio clínico, incluyendo las historias clínicas, CRD y documentos electrónicos o impresos. Esta información permanecerá almacenada en archivos metálicos con llave, permitiendo el acceso a la misma solamente a los especialistas, monitores y comité de dirección del protocolo.

Durante la ejecución del protocolo, la información relacionada con los pacientes será almacenada por duplicado. Una vez que se entregue la misma al centro promotor, quedará conservada una copia en el archivo del sitio clínico.

Los CRD de todos los pacientes serán conservados en soporte físico (CD-R), en el archivo pasivo del Departamento de Ensayos Clínicos del CIGB, por al menos 15 años después de concluido el protocolo.

XI. ESTADÍSTICA

11.1 Número de pacientes planeados

Se considerarán la cantidad de pacientes todos los que dispongan de información según este estudio, en el Hospital Dr. Luis Díaz Soto y cumplan los criterios de inclusión.

11.2 Plan de análisis estadístico

11.2.1 Conjunto de datos analizados

Se distinguen dos grupos:

"Tratado con Jusvinza": definido como los pacientes que hayan sido tartado con el fármaco Jusvinza, según nota recogida en la historia clínica.

"No tratado con Jusvinza": definido como los pacientes en cuyas historias clínicas no se les encontró datos que fueron tratados con el medicamento Jusvinza.

.

XII. ASEGURAMIENTO

El CIGB garantizará los recursos necesarios y la logística que se deriven de la ejecución de este estudio.

XIII. CALENDARIO GENERAL

Etapa	Inicio	Terminación
Coordinación y preparación del protocolo (versión 1.0)	Abril 2021	Abril 2021
Revisión y aprobación por la Dirección de Investigaciones Biomédicas (versión 1.0)	5 Mayo de 2021	7 Mayo de 2021
Revisión y aprobación por Comité de Ética de la Investigación (versión 1.0)	14-20 de Mayo 2021	
Comienzo de la recolección de datos	Mayo 2021	Septiembre 2021
Procesamiento y análisis de los resultados finales	Julio 2021	Septiembre 2021
Elaboración del informe final	Octubre 2021	Noviembre 2021
Envío del primer reporte sobre la evolución de pacientes críticos		Enero 2022
Envío del segundo reporte sobre la evolución de pacientes graves y moderados		Marzo 2022

XIV. CONSIDERACIONES PRÁCTICAS

14.1. Reparto de los deberes y responsabilidades en el protocolo.

14.1.1. Responsabilidades del promotor (CIGB)

a) Realizar proceso diseño y propuesta del estudio con todos los participantes.

b) Designar el monitor responsable del protocolo como representante de los intereses del Promotor.

c) Solicitar informe de pertinencia de la investigación al CECMED.

d) Efectuar el procesamiento estadístico de los datos.

e) Notificar al CECMED los resultados del protocolo para la inclusión en el expediente de registro del producto.

f) Conservar la información primaria y todos los datos durante 15 años.

g) Registrar el ensayo clínico en el RPCEC

14.1.2. Monitores

a) Participar en el diseño y confección del protocolo.

b) Notificar al CECMED el inicio de la ejecución del protocolo.

c) Garantizar el procesamiento de los datos y el tratamiento estadístico que se realizará finalmente.

d) Participar conjuntamente con los especialistas en la confección del informe final con los resultados del protocolo y de los artículos para publicación (procedimiento 4.40.040.00 vigente en la Dirección de Investigaciones Clínicas del CIGB).

14.3.3. Especialistas responsables y su equipo de investigación designado

a) Participar en el diseño y confección del protocolo.

b) Asegurar el cumplimiento de las Buenas Prácticas Clínicas.

c) Utilizar como fuente de información primaria las historias clínicas, registrar la información de cada en los CRD.

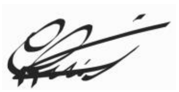

d) Mantener la confidencialidad de la información que se genere durante la ejecución del protocolo. En caso de pretenderse divulgar los resultados se debe consultar la autorización del promotor.

e) Confeccionar todos los informes que se requieran y participar en la discusión y análisis de los resultados del protocolo.

14.4. Procedimientos para el flujo de la documentación

Las historias clínicas serán guardadas en el almacén de documentos del Hospital Dr Luis Diaz Soto. La obtención de los datos de las historias clínicas para redacción de informes y artículos científicos, se hará con la autorización de la dirección del Hospital Dr Luis Diaz Soto.

14.5. Consideraciones sobre los problemas de confidencialidad, divulgación de los resultados y otros aspectos legales

Los especialistas médicos del protocolo, el promotor, los monitores y auditores designados por el promotor garantizarán que los datos de carácter personal de los sujetos incluidos en el protocolo se traten de acuerdo con las previsiones establecidas en la Ley 15/1999 de protección de datos de carácter personal y la normativa que la desarrolla. De igual manera se mantendrá el anonimato de los sujetos incluidos y la protección de su identidad; no se cederá ningún dato de carácter personal de los sujetos del protocolo, excepto en aquellas circunstancias que permita la ley.

Los especialistas el promotor, los monitores y auditores designados por el promotor, se comprometen a tratar la documentación, información, resultados y datos relacionados con el protocolo conforme a su carácter confidencial y secreto, a velar por la circulación restringida de esta información y a hacerse responsable de que esta obligación sea cumplida por todas las personas que deban tener acceso a ella de acuerdo con lo que se establece en este acápite.

Los monitores y auditores designados por el promotor podrán acceder a la información y documentación clínica sobre los sujetos incluidos, a fin de verificar la exactitud y la fiabilidad de los datos, pero no deben recoger los datos personales de identificación de los sujetos. También se debe facilitar el acceso a estos datos a los inspectores de las autoridades sanitarias competentes.

Los resultados del protocolo, así como todos los trabajos e informes realizados y todos los derechos de propiedad industrial derivados de este, son propiedad exclusiva del promotor. Éste está comprometido a

divulgarlos, una vez finalizado el protocolo y ya sean negativos o positivos, en medios de difusión de acceso público.

Se prohíbe la divulgación de cualquier información generada en este protocolo sin el consentimiento del promotor. La publicación de los resultados, por parte de los especialistas médicos de las instituciones hospitalarias, en revistas o libros científicos y las presentaciones orales o carteles en eventos científicos, talleres o reuniones, se tienen que efectuar de común acuerdo con el centro promotor.

Se deberá facilitar al CIGB una copia del manuscrito u original para que pueda conocer su contenido y hacer las comprobaciones oportunas. El CIGB, en un plazo máximo de 30 días, debe comunicar si está de acuerdo o no con el contenido. Si el CIGB considera que es necesario posponer la publicación o presentación propuesta por el especialista, éste deberá hacerlo. Si el CIGB considera que el especialista favorece una interpretación de los datos que puede dañar los derechos del CIGB, se deberá garantizar la integridad científica e intentar adaptar su interpretación de forma que cumpla los criterios del CIGB. Si las partes no llegan a un acuerdo, el especialista deberá incluir en dicha publicación o presentación, la interpretación del CIGB. Vencido el plazo mencionado sin que el promotor haya respondido, se considerará que está de acuerdo y el médico podrá proceder a su publicación o presentación.

El promotor tiene que pedir previamente las autorizaciones correspondientes a los especialistas médicos para poder utilizar sus nombres en publicaciones científicas o en cualquier otro medio de difusión con finalidades comerciales o de divulgación.

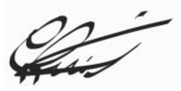

XVI. REFERENCIAS

[1] Xu Z, Shi L, Wang Y, et al. Pathological findings of COVID-19 associated with acute respiratory distress syndrome. Lancet Respir Med 2020; 8:420-422.

[2] Zhou F, Yu T, Du R, et al. Clinical course and risk factors for mortality of adult inpatients with COVID-19 in Wuhan, China: a retrospective cohort study. Lancet 2020; 395: 1054-62.

[3] Domínguez MC, Lorenzo N, Barberá A, Darrasse-Jeze G, Hernandez MV, Torres AM et al. An altered peptide ligand corresponding to a novel epitope from heat-shock protein 60 induces regulatory T cells and suppresses pathogenic response in an animal model of adjuvant induced arthritis. Autoimmunity 2011; 44 :471-82.

[4] Barberá A, Lorenzo N, van Kooten P, et al. APL1, an altered peptide ligand derived from human heat-shock protein 60, increases the frequency of Tregs and its suppressive capacity against antigen responding effector CD4+T cells from rheumatoid arthritis patients. Cell Stress and Chaperones. 2016; 21:735–744.

[5] Lorenzo N, Altruda F, Silengo L and Dominguez MC. APL-1, an altered peptide ligand derived from heat-shock protein, alone or combined with methotrexate attenuates murine collagen induced arthritis. ClinExpMed 2017; 17:209–216.

[6] Domínguez MC, Cabrales A, Lorenzo N, Padrón G and Gonzalez LJ. Biodistribution and pharmacokinetic profiles of an Altered Peptide Ligand derived from Heat-shock proteins 60 in Lewis rats. Cell Stress and Chaperones.2020;25(1):133-140

[7] Dinorah Prada, Jorge Gómez, Norailys Lorenzo, Oreste Corrales, et al. Phase I Clinical Trial with a Novel Altered Peptide Ligand Derived from Human Heat-Shock Protein 60 for Treatment of Rheumatoid Arthritis: Safety, Pharmacokinetics and Preliminary Therapeutic Effects. Journal of Clinical Trials 2018; 8:2167-0870

[8] Cabrales-Rico, A., Ramos, Y., Besada, V., Del Carmen, D. M., Lorenzo, N. et al (2017): Development and validation of a bioanalytical method based on LC-MS/MS analysis for the quantitation of CIGB-814 peptide in plasma from Rheumatoid Arthritis patients. J Pharm.Biomed.Anal. 143: 130-140.

[9] Oreste Corrales, Laura Hernández, Dinorah Prada, et al. CIGB-814, an altered peptide ligand derived from human heat-shock protein 60, decreases anti-cyclic citrullinated peptides antibodies in patients with rheumatoid arthritis. Clinical Rheumatology 2019; 38:955–960.

[10] Venegas-Rodriguez R et al (2020). CIGB-258 Immunomodulatory Peptide: Compassionate Use for Critical and Severe COVID-19 Patients. Austin J Pharmacol Ther 8(1).1119.

[11] Enrique Galbán-García and Pedro Más-Bermejo. (2020). COVID-19 in Cuba: Assessing the National Response. MEDICC Review, Vol 22, No 4

[12] Ministry of Public Health of Cuba. Ministerio de Salud Pública. Cuba: Parte de cierre del día 31 de marzo a las 12 de la noche [Internet] [cited 2021 Mar 31]. Available from: https://salud.msp.gob.cu/parte-de-cierre-del-dia-31-de-marzo-a-las-12-de-la-noche/

[13] Liu J, Liu Y, Xiang P, Pu L, Xiong H, Li C (2020) Neutrophil-to-lymphocyte ratio predicts critical illness patients with 2019 coronavirus disease in the early stage. J Transl Med 18(1):206

[14] Chen L, Long X, Xu Q, Tan J, Wang G, Cao Y (2020) Elevated serum levels of S100A8/A9 and HMGB1 at hospital admission are correlated with inferior clinical outcomes in COVID-19 patients. Cellular and Molecular Immunology 17:992–994.

[15] Zheng M et al (2020) Functional exhaustion of antiviral lymphocytes in COVID-19 patients. Cell Mol Immunol 17:533-535

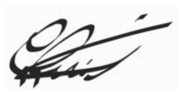

[16] Zheng HY et al (2020) Elevated exhaustion levels and reduced functional diversity of T cells in peripheral blood may predict severe progression in COVID-19 patients. Cell. Mol. Immunol. 17, 541–543.

[17] Hernandez-Cedeño M et al (2021). CIGB-258, a peptide derived from human heat-shock protein 60, decreases hyperinflammation in COVID-19 patients. Cell Stress and Chaperones. DOI: 10.1007/s12192-021-01197-2

18. U.S. Department of Health and Human Services, National Institutes of Health, National Cancer Institute. Common Terminology Criteria for Adverse Events, Version 5.0, November 27, 2017. Available in http://ctep.cancer.gov

19. Naranjo CA, Shear NH, Busto U. Adverse drug reactions. In: Kalant H and Roschlau WHE. Principles of medical pharmacology. 6th ed. New York: Oxford University Press, 1998:791-800.

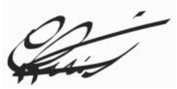

Supplement: S2 Method — (PDF) [file pone.0281111.s003.pdf]
