# Supplementary material for: Jusvinza, an anti-inflammatory drug derived from the human heat-shock protein 60, for critically ill COVID-19 patients. An observational study
Source: PLoS One. 2023 Feb 2;18(2):e0281111. doi: 10.1371/journal.pone.0281111 (PMC9894446; doi:10.1371/journal.pone.0281111)
Supplement: S1 Table — Serial analyses of clinical parameters and plasma biomarkers at T0, 48 hours and days 7. (PDF) [file pone.0281111.s004.pdf]

Legends:

Antes de comenzar el tratamiento: Before beginning treatment **Código**: code **Desenlace**: Outcome **F.Ingreso**: Inicial date **Final tto**: After treatment **Edad**: Age

Raza: Race **IMC**: body mass index **APP**: comorbidities **FC**: heart rate **PA**: blood pressure **PAM**: mean arterial pressure **FR**: respiration rate **Temp**: temperature

INL: Neutrophil/lymphocyte rate **Ferritina**: ferritine **PCR**: C reactive protein **Eritro**: erythrocyte sedimentation rate **Fibrinógeno**: fibrinogen **DD**: D-dimer **V**: Alive
F: dead **HTA**: Arterial Hypertension **AB**: Bronchial Asthma **DM**: Mellitus Diabetes **CI**: Ischemic Heart **EPOC**: Chronic obstructive pulmonary disease **O**: obese
NP: normal weigth **BP**: low weigth SP: overweigth B: White race M: mestiza race

**Jusvinza**								**Antes de comenzar el tratamiento**															
**Código**	**Desenlace**	**F .Ingreso**	**Final tto**	**Edad**	**Raza**	**IMC**	**APP**	**FC**	**PA**	**PAM**	**FR**	**Temp**	**SO_2**	**PO_2**	**P/F**	**INL**	**LDH**	**Ferritina**	**PCR**	**Eritro**	**Fibrinógeno**	**DD**	**IL-6**
1	V	21.3.21	28.3.21	71	B	SP	HTA	112	100/60	73	32	36	87	59	197	18	980	1112	22	111	789	11	
2	V	24.3.21	2.4.21	76	B	SP	CI,HTA	100	110/70	83	40	38	91	56	187	22	1113	1456	22	125	900	15	
3	V	22.3.21	27.3.21	65	B	NP	DM,HTA	111	150/100	116	37	38	90	56	187	26	980	988	19	121	890	16	
4	V	26.3.21	3.4.21	71	B	SP	EPOC	118	100/60	73	35	37	87	56	187	21	978	985	18	112	678	8	
5	V	30.3.21	4.4.21	65	B	NP	HTA	112	80/40	53	36	35	93	50	167	15	1231	899	17	121	974	9	
6	F	21.3.21	24.3.21	65	B	SP	HTA	100	100/60	73	39	38	91	52	173	22	978	1000	26	178	900	12	
7	V	28.3.21	6.4.21	65	B	O	HTA	119	90/50	63	35	37	88	52	173	16	960	987	19	141	567	9	
8	V	23.3.21	1.4.21	69	B	O	HTA, DM	104	90/70	76	35	38	85	55	183	9	1678	1345	31	125	890	12	
9	V	27.3.21	3.4.21	72	M	BP	AB, HTA	119	80/40	53	21	38	89	54	180	19	1456	1768	28	98	900	9	
10	V	21.3.21	28.3.21	66	B	O	HTA	115	90/60	70	36	38	94	55	183	12	980	897	22	112	890	15	
11	V	24.3.21	30.3.21	71	B	SP	HTA	112	100/60	73	32	36	87	59	197	18	980	1112	22	111	789	11	
12	V	25.3.21	31.3.21	76	B	SP	CI,HTA	100	110/70	83	40	38	91	56	187	22	1242	1456	22	125	900	15	
13	V	28.3.21	3.4.21	65	B	NP	DM,HTA	111	150/100	116	37	38	90	56	187	26	980	988	19	121	890	16	
14	F	24.3.21	28.3.21	67	B	O	HTA	99	90/60	75	32	39	90	55	183	21	976	1112	27	188	988	13	
15	V	31.3.21	7.4.21	65	B	O	HTA	129	90/50	63	35	37	90	52	173	16	960	987	19	141	567	9	
16	V	22.3.21	30.3.21	69	B	O	CI, HTA	132	90/70	76	35	38	85	55	188	9	1221	1345	31	125	890	12	
17	V	27.3.21	5.4.21	72	B	O	CI, HTA	119	80/40	53	32	38	89	54	180	19	1168	1768	28	165	900	16	
18	V	25.3.21	2.4.21	71	B	SP	DM	118	100/60	73	35	37	87	56	187	21	978	987	18	126	678	22	

19	V	21.3.21	28.3.21	65	B	NP	EPOC	127	80/40	53	36	35	93	50	190	15	1231	899	17	121	974	21	
20	F	22.3.21	25.3.22	69	B	SP	HTA	112	100/60	72	29	37.5	91	51	170	19	945	998	28	167	956	12	
21	V	24.3.21	31.3.21	65	B	BP	HTA	119	90/50	63	35	37	88	52	189	16	960	987	19	112	567	9	
22	V	1.4.21	9.4.21	69	M	O	CI, HTA	129	90/70	76	35	38	90	55	183	9	998	1345	31	125	890	12	
23	V	20.4.21	28.4.21	72	M	O	DM, HTA	119	80/40	53	34	38	89	54	180	19	1456	1768	28	98	900	11	
24	V	2.4.21	9.4.21	71	B	SP	HTA	118	100/60	73	35	37	90	56	187	21	978	1321	18	126	897	15	
25	V	5.4.21	11.4.21	65	B	NP	EPOC	127	80/40	53	36	35	93	50	187	15	1231	899	17	121	974	9	
26	F	28.3.21	2.4.21	65	B	SP	DM,HTA	100	110/60	76	39	38	89	54	180	23	988	1120	32	187	899	16	
27	V	26.4.21	4.5.21	71	B	SP	HTA	112	100/60	73	32	36	87	59	197	18	980	1112	22	111	789	11	
28	V	11.4.21	19.4.21	76	B	SP	CI,HTA	135	110/70	83	40	38	91	56	187	22	999	1456	22	125	900	15	
29	V	17.4.21	23.4.21	65	B	NP	DM,HTA	132	150/100	116	37	38	90	56	187	26	980	988	19	121	890	16	
30	V	22.4.21	29.4.21	71	B	SP	DM	118	100/60	73	35	37	87	56	187	21	978	789	18	126	678	12	
31	V	7.3.21	13.3.21	65	B	NP	HTA	129	80/40	53	36	35	93	50	190	15	1231	899	17	121	974	14	
32	F	28.3.21	1.4.21	77	B	BP	HTA	102	100/50	69	37	37	91	55	183	22	899	987	26	169	904	11	
33	V	30.4.21	8.5.21	65	B	O	HTA	119	90/50	63	35	37	90	52	190	16	960	987	19	141	567	9	
34	V	15.4.21	23.4.21	69	B	O	CI, HTA	121	90/70	76	35	38	90	55	183	9	1899	1345	31	125	890	12	
35	V	24.3.21	3.4.21	72	B	O	AB, HTA	119	80/40	53	37	38	89	54	180	19	1456	1768	28	155	900	16	
36	V	11.4.21	18.4.21	71	B	SP	DM	118	100/60	73	35	37	87	56	187	21	978	978	18	126	678	13	
37	V	29.4.21	5.6.21	65	B	NP	HTA	112	80/40	53	36	35	93	50	190	15	1231	899	17	121	974	9	
38	V	13.4.21	21.4.21	65	B	O	HTA	119	90/50	63	35	37	88	52	187	16	960	987	19	141	567	9	
39	V	20.4.21	28.4.21	69	M	O	HTA, CI	138	90/70	76	35	38	90	55	183	9	1766	1345	31	125	890	12	
40	V	6.4.21	14.4.21	72	B	BP	CI, HTA	119	80/40	53	21	38	89	54	180	19	1456	1768	28	165	900	9	
41	F	1.4.21	4.4.21	65	B	SP	HTA	109	100/60	67	39	38	90	50	167	22	979	999	29	188	911	14	
42	V	4.4.21	13.4.21	69	M	O	GASTRITIS	126	90/70	76	35	38	85	55	183	9	1677	1345	31	125	890	12	
43	V	14.3.21	23.3.21	72	B	O	DM, HTA	119	80/40	53	31	38	89	54	188	19	1456	1768	28	165	900	14	
44	F	4.4.21	7.4.21	78	B	NP	HTA	99	98/60	73	38	38	88	52	173	21	973	958	31	188	879	11	
45	V	17.3.21	26.3.21	72	M	O	CI, HTA	119	80/40	53	29	38	89	54	180	19	1456	1768	28	165	900	9	
46	V	25.4.21	4.5.21	65	B	O	HTA	119	90/50	63	35	37	88	52	173	16	960	987	19	141	567	9	
47	V	1.5.21	10.5.21	72	B	O	DM, HTA	129	80/40	53	28	38	89	54	180	19	1456	1768	28	165	900	14	175.93
48	F	22.3.21	27.3.21	65	M	NP	DM	100	110/60	77	37	38	91	53	177	19	969	976	29	179	799	10	
49	V	17.5.21	23.5.21	65	B	BP	HTA	119	90/50	63	35	37	88	52	183	16	960	987	19	141	567	9	59.6
50	V	21.5.21	30.5.21	69	M	O	GASTRITIS	125	90/70	76	35	38	85	55	183	9	1700	1345	31	125	890	12	
51	V	26.5.21	3.6.21	72	M	O	AB, HTA	119	80/40	53	28	38	89	54	180	19	1456	1768	28	165	900	9	

52	V	28.4.21	6.5.21	71	B	SP	EPOC	118	100/60	73	35	37	87	56	187	21	978	789	18	126	678	15	
53	V	19.5.21	26.5.21	65	B	NP	HTA	112	80/40	53	36	35	93	50	167	15	1231	899	17	121	974	9	59.79
54	V	12.5.21	21.5.21	72	B	O	DM, HTA	119	80/40	53	31	38	89	54	180	19	1456	1768	28	165	900	9	148.88
55	V	30.5.21	8.6.21	65	B	O	HTA	119	90/50	63	35	37	88	52	183	16	960	987	19	141	567	9	
56	V	19.5.21	26.5.21	65	B	BP	HTA	119	90/50	63	35	37	90	52	183	16	960	987	19	141	567	9	139.71
57	V	7.5.21	16.5.21	69	B	O	HTA, DM	129	90/70	76	35	38	90	55	183	9	965	1345	31	125	890	12	46.49
58	V	29.5.21	7.6.21	72	M	O	AB, HTA	119	80/40	53	21	38	89	54	180	19	1456	1768	28	115	900	13	
59	V	22.4.21	2.5.21	71	B	SP	HTA	118	100/60	73	35	37	87	56	187	21	978	959	18	126	678	19	
60	V	22.5.21	29.5.21	65	B	NP	DM	112	80/40	53	36	35	93	50	167	15	1231	899	17	121	974	9	
61	F	27.4.21	1.5.21	81	B	SP	HTA	112	100/60	69	39	39	92	51	170	17	977	988	28	176	868	12	
62	V	8.5.21	19.5.21	65	B	O	HTA	119	90/50	63	35	37	88	52	173	16	960	987	19	141	567	16	79.6
63	V	16.3.21	25.3.21	69	B	O	HTA, CI	138	90/70	76	35	38	90	55	183	9	1899	1345	31	125	890	12	
64	V	21.3.21	30.3.21	72	B	O	DM, HTA	119	80/40	53	21	38	89	54	180	19	1456	1768	13	165	900	9	
65	V	26.4.21	5.5.21	71	B	SP	EPOC	118	100/60	73	35	37	87	56	187	21	978	1235	15	126	678	17	
66	V	29.5.21	5.6.21	65	B	NP	DM	112	80/40	53	36	35	93	50	167	15	1231	899	17	121	974	16	
67	V	19.3.21	26.3.21	76	B	SP	CI,HTA	129	110/70	83	35	38	91	56	187	22	1242	1456	22	125	900	15	
68	V	11.6.21	19.6.21	65	B	O	HTA	119	90/50	63	35	37	88	52	173	16	960	987	19	141	567	9	
69	V	30.5.21	7.6.21	69	B	O	CI, HTA	104	90/70	76	35	38	85	55	183	9	1899	1345	31	125	890	12	
70	V	6.6.21	14.6.21	72	B	O	AB, HTA	119	80/40	53	33	38	89	54	180	19	1456	1768	28	125	900	9	
71	V	25.3.21	3.4.21	71	B	SP	HTA	137	100/60	73	35	37	87	56	187	21	978	989	18	126	678	8	
72	V	1.6.21	8.6.21	65	B	NP	AB	114	80/40	53	36	35	93	50	167	15	1231	899	17	121	974	9	
73	F	21.3.21	24.3.21	65	B	BP	DM,HTA	115	90/60	79	40	39	91	55	183	19	898	999	28	169	987	12	
74	V	20.5.21	28.5.21	65	B	SP	HTA	119	90/50	63	35	37	91	52	173	16	960	987	19	141	567	9	
75	V	22.4.21	3.5.21	69	B	O	GASTRITIS	129	90/70	76	35	38	85	55	183	9	1452	1345	31	125	890	12	
76	V	2.6.21	11.6.21	72	M	O	AB, HTA	135	80/40	53	40	38	89	54	180	19	1456	1768	28	165	900	9	
77	V	27.4.21	5.4.21	71	B	SP	HTA	128	100/60	73	35	37	87	56	187	21	978	999	18	126	678	11	
78	V	22.5.21	28.5.21	65	B	NP	DM	112	80/40	53	36	35	93	50	167	15	1231	899	17	121	974	9	
79	V	5.4.21	14.4.21	72	B	O	DM, HTA	119	80/40	53	33	38	89	54	180	19	1456	1768	28	165	900	9	
80	V	9.6.21	17.6.21	65	B	SP	HTA	123	90/50	63	35	37	88	52	188	16	960	987	19	141	567	9	
81	F	29.3.21	2.4.21	66	B	SP	HTA	100	100/60	70	29	38	93	52	173	25	981	989	29	187	948	15	
82	V	30.5.21	8.6.21	65	B	SP	HTA	119	90/50	63	35	37	88	52	190	16	960	987	19	141	567	9	
83	V	18.4.21	21.4.21	69	B	O	HTA, DM	130	90/70	76	35	38	85	55	183	9	1899	1345	15	125	890	12	
84	V	16.6.21	25.6.21	72	B	SP	CI, HTA	119	80/40	53	32	38	89	54	180	19	1456	1768	28	165	900	22	

85	V	15.3.21	24.3.21	65	B	O	HTA	130	90/50	63	35	37	88	52	190	16	960	987	19	141	567	9	
86	V	22.5.21	30.5.21	69	B	SP	CI, HTA	128	90/70	76	35	38	90	55	183	9	1655	1345	31	125	890	12	
87	V	4.6.21	13.6.21	72	B	O	DM, HTA	119	80/40	53	33	38	89	54	180	19	1456	1768	28	165	900	19	
88	V	6.4.21	15.4.21	71	B	SP	HTA	122	100/60	73	35	37	87	56	187	21	978	1269	18	126	678	11	
89	V	21.5.21	28.5.21	65	B	NP	DM	127	80/40	53	36	35	93	50	167	15	1231	899	17	121	974	33	
90	V	29.3.21	6.4.21	65	B	O	HTA	119	90/50	63	35	37	90	52	183	16	960	987	19	141	1213	9	
91	V	18.6.21	29.6.21	69	B	SP	GASTRITIS	127	90/70	76	35	38	85	55	183	9	1899	1345	19	125	890	12	
92	V	22.4.21	1.5.21	72	B	BP	DM, HTA	129	80/40	53	32	38	89	54	180	19	1456	1768	28	165	900	15	
93	V	6.6.21	15.6.21	71	B	SP	EPOC	118	100/60	73	35	37	90	56	187	21	978	1231	18	98	1456	22	
94	V	8.6.21	17.6.21	65	B	NP	AB	128	80/40	53	36	35	93	50	187	15	1231	899	17	121	974	22	
95	V	10.6.21	21.6.21	65	B	SP	HTA	119	90/50	63	35	37	92	52	173	16	960	987	19	141	567	18	
96	V	24.5.21	2.6.21	69	B	O	GASTRITIS	127	90/70	76	35	38	93	55	183	9	1621	1345	31	125	890	12	
97	V	19.4.21	28.4.21	72	M	O	AB, HTA	132	80/40	53	34	38	89	54	180	19	1215	1768	18	165	900	19	
98	V	1.6.21	10.6.21	65	B	SP	HTA	123	90/50	63	35	37	92	52	173	16	960	987	19	141	567	18	
99	V	15.5.21	29.6.21	69	B	O	HTA, DM	133	90/70	76	35	38	89	55	183	9	1543	1345	31	125	890	12	2.73
100	V	12.5.21	21.5.21	72	M	O	DM, HTA	119	80/40	53	38	38	89	54	180	19	1456	1768	28	154	900	18	80.32
101	V	2.5.21	11.5.21	71	B	SP	HTA	133	100/60	73	35	37	90	56	187	21	978	1381	18	126	678	19	298.7
102	V	18.3.21	25.3.21	65	B	NP	DM	128	80/40	53	36	35	93	50	167	15	978	899	17	121	974	21	
103	V	9.6.21	16.6.21	65	B	NP	DM,HTA	139	150/100	116	37	38	90	56	187	26	980	988	19	121	890	16	
104	V	19.4.21	27.4.21	71	B	SP	HTA	118	100/60	73	35	37	88	56	187	21	978	976	18	126	678	15	
105	F	28.5.21	1.6.21	75	B	O	HTA,DM	98	100/60	66	40	40	91	55	183	23	979	979	30	179	901	9	
106	V	29.5.21	5.6.21	65	B	BP	HTA	128	90/50	63	24	37	90	52	190	16	960	987	15	111	567	19	
107	V	12.4.21	21.4.21	69	M	O	GASTRITIS	128	90/70	76	35	38	85	55	183	31	1225	1345	31	125	1345	12	
108	V	19.5.21	27.5.21	72	B	SP	AB, HTA	119	80/40	53	33	38	89	54	180	19	689	1768	21	121	900	22	477.3
109	V	28.3.21	5.4.21	65	B	SP	HTA	119	90/50	63	28	37	92	52	190	16	960	987	19	121	567	18	
110	V	7.6.21	15.6.21	69	B	O	HTA, CI	139	90/70	76	38	38	90	55	183	28	998	1345	31	125	890	12	
111	V	30.5.21	7.6.21	72	B	SP	AB, HTA	119	80/40	53	35	38	89	54	180	19	1111	1768	28	99	900	17	
112	V	22.3.21	30.3.21	71	B	SP	DM	128	100/60	73	35	37	87	56	187	21	978	987	18	96	912	15	
113	V	15.5.21	22.5.21	65	B	NP	HTA	128	80/40	53	36	35	93	50	167	15	789	998	17	121	974	21	37.9
114	V	18.6.21	29.6.21	65	B	O	HTA	125	90/50	63	33	37	90	52	190	16	960	999	19	136	879	17	
115	V	18.4.21	27.4.21	69	B	SP	CI, HTA	142	90/70	76	35	38	90	55	183	20	1221	1345	22	125	890	12	
116	V	6.6.21	15.6.21	72	B	O	DM, HTA	130	80/40	53	40	38	89	54	180	19	1121	1768	11	98	876	22	
117	V	9.6.21	18.6.21	71	B	SP	DM	129	100/60	73	35	37	90	56	187	21	978	1100	18	98	678	15	

118	V	3.5.21	9.5.21	65	B	NP	HTA	125	80/40	53	36	35	93	50	190	15	1231	1358	17	121	974	25	126.1
119	V	6.6.21	13.6.21	76	B	SP	CI,HTA	124	110/70	83	34	38	91	56	187	22	1021	1456	22	125	887	15	
120	V	30.5.21	6.6.21	65	B	SP	HTA	128	90/50	63	35	37	90	52	190	16	960	1123	19	123	969	22	
121	V	10.6.21	21.6.21	69	B	O	HTA, DM	123	90/70	76	35	38	90	55	183	21	1654	1345	21	125	890	12	
122	V	30.5.21	8.6.21	72	B	SP	DM, HTA	119	80/40	53	37	38	93	54	180	19	1200	1500	12	165	900	9	
123	V	22.4.21	30.4.21	71	B	SP	HTA	118	100/60	73	35	37	87	56	187	21	978	789	18	98	678	21	
124	V	5.3.21	11.3.21	65	B	NP	DM	136	80/40	53	36	35	93	50	167	15	1231	899	17	121	974	21	
125	V	18.6.21	29.6.21	76	B	SP	AB	139	110/70	83	40	38	91	56	187	22	999	1456	22	98	900	15	

**Jusvinza**	**48h**														
**Código**	**FC**	**PA**	**PAM**	**FR**	**Temp**	**SO_2**	**PO_2**	**P/F**	**INL**	**LDH**	**Ferritina**	**PCR**	**Eritro**	**Fibrinógeno**	**DD**
1	78	120/70	86	25	36	94	77	257	9	549	754	9	99	389	6
2	79	120/80	93	22	36	95	77	257	11	890	689	11	98	760	11
3	89	115/90	98	21	35	94	68	227	14	786	651	11	76	689	9
4	88	110/70	83	19	35	95	68	227	13	632	467	11	78	459	3
5	89	115/70	85	18	35	96	72	240	8	542	499	10	89	616	6
6	80	110/70	83	38	36	96	67	112	25	1798	1232	31	224	915	18
7	89	110/70	83	22	35	95	66	220	7	659	678	10	89	421	5
8	87	110/80	90	19	36	95	69	138	5	978	659	22	98	634	4
9	89	100/60	73	22	35	94	72	240	11	689	689	19	99	589	6
10	89	110/60	76	20	36	95	68	227	5	589	587	12	78	624	9
11	86	120/70	86	25	36	94	77	257	9	549	754	9	99	698	8
12	100	120/80	93	22	36	95	77	257	11	890	689	11	98	760	11
13	100	115/90	98	21	35	94	68	227	14	786	651	11	76	689	9
14	89	100/70	80	35	37	95	70	117	27	1467	1115	22	226	1111	19
15	89	110/70	83	22	35	95	66	220	7	659	678	10	76	526	6
16	100	110/80	90	19	36	95	69	235	5	978	659	22	98	634	4
17	89	100/60	73	22	35	94	72	240	11	689	765	19	99	689	6
18	92	110/70	83	19	35	95	68	227	13	632	467	11	101	687	8
19	89	115/70	85	18	35	96	72	240	8	542	499	10	89	616	8
20	99	110/70	83	30	35.7	96	68	113	25	1679	1221	31	224	987	16

21	89	110/70	83	22	35	95	66	220	7	659	678	10	111	678	5
22	100	110/80	90	19	36	95	69	212	5	978	659	22	98	634	5
23	99	100/60	73	22	35	94	72	240	11	689	765	19	99	689	6
24	88	110/70	83	19	35	95	68	227	13	632	467	11	101	659	8
25	89	115/70	85	18	35	96	72	240	8	542	499	10	89	616	6
26	88	110/70	83	37	36	96	67	112	24	1798	1324	29	226	919	18
27	89	120/70	86	25	36	94	77	257	9	549	754	9	99	632	8
28	88	120/80	93	22	36	95	77	257	11	890	689	11	98	760	11
29	89	115/90	98	21	35	94	68	227	14	786	455	11	76	689	9
30	100	110/70	83	19	35	95	68	227	13	632	467	11	89	659	8
31	89	115/70	85	18	35	96	72	240	8	542	499	10	89	616	6
32	87	110/70	83	36	37	96	66	110	25	1689	1267	31	224	917	16
33	89	110/70	83	22	35	95	66	220	7	659	678	10	111	641	9
34	93	110/80	90	19	36	95	69	138	5	978	659	22	98	634	9
35	89	100/60	73	22	35	94	72	240	11	689	765	19	99	689	8
36	88	110/70	83	19	35	95	68	227	13	632	467	11	101	669	8
37	99	115/70	85	18	35	96	72	240	8	542	499	10	89	616	6
38	100	110/70	83	22	35	95	66	220	7	659	678	10	111	521	9
39	87	110/80	90	19	36	95	69	138	5	978	659	22	98	634	7
40	89	100/60	73	22	35	94	72	240	11	689	765	19	99	687	6
41	89	110/70	83	36	35	94	67	112	26	1659	1274	28	221	998	18
42	92	110/80	90	19	36	95	69	225	5	978	659	22	98	634	8
43	89	100/60	73	22	35	94	72	240	11	689	765	19	99	689	6
44	78	100/70	80	35	36	96	68	113	25	1893	1287	30	222	1254	26
45	89	100/60	73	22	35	94	72	240	11	689	765	19	99	589	6
46	89	110/70	83	22	35	95	66	220	7	659	678	10	89	621	9
47	89	100/60	73	22	35	94	72	240	11	689	765	19	99	689	6
48	85	100/70	80	33	35.6	96	67	112	23	1764	1310	31	224	915	24
49	89	110/70	83	22	35	95	66	220	7	659	678	10	99	615	5
50	100	110/80	90	19	36	95	69	232	5	978	456	22	98	634	9
51	89	100/60	73	22	35	94	72	240	11	689	765	19	99	689	6
52	88	110/70	83	19	35	95	68	227	13	632	467	11	101	659	7
53	99	115/70	85	18	35	96	72	240	8	542	499	10	89	616	6

54	89	100/60	73	22	35	94	72	240	11	689	765	19	99	689	6
55	89	110/70	83	22	35	95	66	220	7	659	678	10	111	643	9
56	89	110/70	83	22	35	95	66	220	7	659	678	10	111	645	9
57	100	110/80	90	19	36	95	69	245	5	978	659	22	98	634	9
58	89	100/60	73	22	35	94	72	240	11	689	765	19	99	589	6
59	88	110/70	83	19	35	95	68	227	13	632	467	11	79	648	9
60	89	115/70	85	18	35	96	72	240	8	542	499	10	78	616	6
61	80	110/70	83	38	37	95	70	117	21	1782	1286	27	231	915	27
62	89	110/70	83	22	35	95	66	220	7	659	678	10	111	622	5
63	100	110/80	90	19	36	95	69	255	5	978	659	22	98	634	8
64	89	100/60	73	22	35	94	72	240	11	689	765	19	99	689	6
65	88	110/70	83	19	35	95	68	227	13	632	467	11	66	678	8
66	89	115/70	85	18	35	96	72	240	8	542	499	10	89	616	6
67	88	120/80	93	22	36	95	77	257	11	890	689	11	98	760	11
68	89	110/70	83	22	35	95	66	220	7	659	678	10	111	642	5
69	87	110/80	90	19	36	95	69	138	5	978	431	22	98	634	8
70	89	100/60	73	22	35	94	72	240	11	689	765	19	99	589	6
71	88	110/70	83	19	35	95	68	227	13	632	467	11	59	559	8
72	89	115/70	85	18	35	96	72	240	8	542	499	10	89	616	6
73	77	100/70	80	35	36	96	67	112	25	1798	1274	25	228	927	16
74	100	110/70	83	22	35	95	66	220	7	659	678	10	111	525	9
75	92	110/80	90	19	36	95	69	221	5	978	659	22	98	634	8
76	89	100/60	73	22	35	94	72	240	11	689	765	19	99	689	6
77	99	110/70	83	19	35	95	68	227	13	632	467	11	58	669	8
78	89	115/70	85	18	35	96	72	240	8	542	499	10	89	616	6
79	89	100/60	73	22	35	94	72	240	11	689	765	19	99	689	6
80	89	110/70	83	22	35	95	66	220	7	659	289	10	111	621	8
81	76	110/70	83	30	35	96	77	128	28	1699	1234	19	223	987	27
82	89	110/70	83	22	35	95	66	220	7	659	678	10	49	567	5
83	92	110/80	90	19	36	95	69	138	5	978	659	22	98	634	9
84	89	100/60	73	22	35	94	72	240	11	689	765	19	99	589	6
85	89	110/70	83	22	35	95	66	220	7	659	678	10	66	621	8
86	100	110/80	90	19	36	95	69	259	5	978	659	22	98	634	8

87	92	100/60	73	22	35	94	72	240	11	689	765	19	99	689	6
88	88	110/70	83	19	35	95	68	227	13	632	467	11	66	654	6
89	89	115/70	85	18	35	96	72	240	8	542	499	10	89	616	6
90	89	110/70	83	22	35	95	66	220	7	659	678	10	63	631	8
91	87	110/80	90	19	36	95	69	138	5	978	659	22	98	634	6
92	89	100/60	73	22	35	94	72	240	11	689	765	19	99	689	6
93	88	110/70	83	19	35	95	68	227	13	632	467	11	59	656	7
94	89	115/70	85	18	35	96	72	240	8	542	499	10	89	616	6
95	89	110/70	83	22	35	95	66	220	7	659	678	10	76	621	8
96	99	110/80	90	19	36	95	69	138	5	978	659	22	98	634	8
97	89	100/60	73	22	35	94	72	240	11	689	765	19	99	589	6
98	89	110/70	83	22	35	95	66	220	7	659	678	10	65	598	8
99	90	110/80	90	19	36	95	69	138	5	978	659	22	98	634	5
100	89	100/60	73	22	35	94	72	240	11	689	765	19	99	789	6
101	88	110/70	83	19	35	95	68	227	13	632	467	11	79	677	8
102	89	115/70	85	18	35	96	72	240	8	542	499	10	89	616	6
103	89	115/90	98	21	35	94	68	227	14	786	651	11	76	689	9
104	100	110/70	83	20	35	95	68	227	13	632	467	11	76	615	8
105	81	110/70	83	38	36	97	78	130	26	1689	1232	33	230	1342	25
106	89	110/70	83	22	35	95	66	220	7	659	678	10	79	698	8
107	94	110/80	90	19	36	95	69	138	5	978	659	22	98	634	8
108	89	100/60	73	22	35	94	72	240	11	689	765	19	99	689	6
109	89	110/70	83	22	35	95	66	220	7	659	678	10	89	664	9
110	90	110/80	90	20	36	95	69	138	5	978	659	22	98	634	8
111	89	100/60	73	22	35	94	72	240	11	689	765	19	99	689	6
112	100	110/70	83	19	35	95	68	227	13	632	467	11	66	679	8
113	89	115/70	85	18	35	96	72	240	8	542	499	10	89	616	9
114	89	110/70	83	22	35	95	66	220	7	659	339	10	99	643	8
115	93	110/80	90	19	36	95	69	138	5	978	457	22	98	634	8
116	89	100/60	73	22	35	94	72	240	11	689	765	19	99	650	6
117	100	110/70	83	22	35	95	68	227	13	632	467	11	76	789	8
118	89	115/70	85	18	35	96	72	240	8	542	499	10	89	616	6
119	90	120/80	93	22	36	95	77	257	11	890	689	11	98	760	11

120	89	110/70	83	22	35	95	66	220	7	765	435	10	89	678	8
121	92	110/80	90	19	36	95	69	235	5	943	659	22	98	634	8
122	89	100/60	73	22	35	94	72	240	11	689	765	19	99	589	8
123	99	110/70	83	19	35	95	68	227	13	765	467	11	67	768	8
124	89	115/70	85	18	35	96	72	240	8	542	499	10	89	616	6
125	100	120/80	93	22	36	95	77	257	11	890	431	11	98	760	11

Jusvinza	72h															96h
Código	FC	PA	PAM	FR	Temp	SO_2	PO_2	P/F	INL	LDH	Ferritina	PCR	Eritro	Fibrinógeno	DD	IL-6
1	78	120/70	86	22	36	94	88	293	6	439	421	6	65	388	2	
2	76	120/80	93	22	36	95	79	263	7	453	589	4	69	560	2	
3	87	115/90	98	21	35	96	75	250	6	587	378	5	65	455	2	
4	89	110/70	83	19	35	95	79	263	8	379	243	7	68	421	4	
5	76	115/70	85	18	35	96	85	283	7	379	459	8	21	476	5	
6	135	110/70	83	38	36	84	55	79	39	1956	1345	33	232	987	32	
7	89	110/70	83	19	35	96	80	267	5	564	433	5	68	411	3	
8	85	110/80	90	19	36	95	75	250	4	516	639	11	65	456	4	
9	81	100/60	73	22	35	94	77	257	5	578	321	7	55	476	4	
10	92	110/60	76	18	36	96	80	267	3	439	333	9	55	455	4	
11	78	120/70	86	22	36	94	88	293	6	439	421	6	77	388	4	
12	76	120/80	93	22	36	95	79	263	7	659	589	4	69	460	4	
13	87	115/90	98	21	35	96	75	250	9	587	378	5	65	455	4	
14	114	110/70	83	38	34	82	66	94	31	1945	1215	24	238	986	33	
15	89	110/70	83	19	35	96	80	267	5	564	433	5	68	411	3	
16	85	110/80	90	19	36	95	75	250	4	453	639	11	65	456	5	
17	81	100/60	73	22	35	94	77	257	5	578	321	7	55	476	4	
18	89	110/70	83	19	35	95	79	290	8	379	243	7	68	421	5	
19	76	115/70	85	18	35	96	85	283	8	379	459	8	21	476	5	
20	122	110/70	83	38	28	84	65	93	29	1779	º321	33	132	994	30	
21	89	110/70	83	19	35	96	80	267	5	564	433	5	68	411	5	
22	85	110/80	90	19	36	95	75	250	8	645	639	11	65	456	4	
23	81	100/60	73	22	35	94	77	257	5	578	321	7	55	476	4	

24	89	110/70	83	19	35	95	79	263	8	379	243	7	68	421	4	
25	76	115/70	85	18	35	96	85	283	7	379	459	8	45	476	5	
26	117	100/70	80	38	35	88	55	79	29	1898	1424	31	234	928	32	
27	78	120/70	86	22	36	94	88	293	6	439	421	6	65	388	5	
28	76	120/80	93	22	36	95	79	263	7	659	589	4	76	464	5	
29	87	115/90	98	21	35	96	75	250	9	587	378	5	65	455	2	
30	89	110/70	83	19	35	95	79	263	8	379	243	7	68	421	4	
31	76	115/70	85	18	35	96	85	283	8	379	459	8	56	476	5	
32	111	110/70	83	38	34	86	58	83	30	1789	1368	33	232	877	30	
33	89	110/70	83	19	35	96	80	267	5	564	433	5	68	411	3	
34	85	110/80	90	19	36	95	75	250	4	645	639	11	65	456	4	
35	81	100/60	73	22	35	94	77	257	5	578	321	7	55	476	4	
36	89	110/70	83	19	35	95	79	263	8	379	243	7	68	421	4	
37	76	115/70	85	18	35	96	85	283	8	379	459	8	21	476	5	
38	89	110/70	83	19	35	96	80	267	5	564	433	5	68	411	5	
39	85	110/80	90	19	36	95	75	250	4	578	639	11	65	456	4	
40	81	100/60	73	22	35	94	77	257	5	578	321	7	55	476	4	
41	121	100/70	80	38	34	84	55	79	30	1759	1375	30	229	993	32	
42	85	110/80	90	19	36	95	75	250	4	645	639	11	65	456	4	
43	81	100/60	73	22	35	94	77	257	7	578	321	7	55	476	4	
44	117	110/70	83	38	33	85	53	76	29	1993	1388	32	232	985	30	
45	81	100/60	73	22	35	94	77	257	5	578	321	7	55	476	4	
46	89	110/70	83	19	35	96	80	267	5	315	433	5	68	411	3	
47	81	100/60	73	22	35	94	77	257	5	278	321	7	55	476	4	1.31
48	119	110/70	83	38	3º	84	55	79	27	1864	1415	33	231	983	28	
49	89	110/70	83	19	35	96	80	267	5	453	433	5	68	411	3	6.83
50	85	110/80	90	19	36	95	75	250	4	645	639	11	65	456	4	
51	81	100/60	73	22	35	94	77	257	9	578	321	7	55	476	4	
52	89	110/70	83	19	35	95	79	263	8	379	243	7	68	421	4	
53	76	115/70	85	18	35	96	85	283	7	379	459	8	21	476	5	0.88
54	81	100/60	73	22	35	94	77	257	5	578	321	7	55	476	4	50.79
55	89	110/70	83	19	35	96	80	267	5	564	433	5	68	411	3	
56	89	110/70	83	19	35	96	80	267	5	431	433	5	68	411	5	28.01

57	85	110/80	90	19	36	95	75	250	4	564	639	11	65	456	4	17.46
58	81	100/60	73	22	35	94	77	257	5	578	321	7	55	476	4	
59	89	110/70	83	19	35	95	79	263	8	379	243	7	68	421	4	
60	76	115/70	85	18	35	96	85	283	6	379	459	8	39	476	5	
61	135	100/70	80	38	36	87	51	73	25	1882	1387	29	239	999	31	
62	89	110/70	83	19	35	96	80	267	5	564	433	5	68	411	3	3.94
63	85	110/80	90	19	36	95	75	250	4	326	639	11	65	456	4	
64	81	100/60	73	22	35	94	77	257	5	418	321	7	55	476	4	
65	89	110/70	83	19	35	95	79	263	8	379	243	7	68	421	4	
66	76	115/70	85	18	35	96	85	283	9	379	459	8	45	476	5	
67	76	120/80	93	22	36	95	79	263	7	435	589	4	69	560	4	
68	89	110/70	83	19	35	96	80	267	5	399	433	5	68	411	5	
69	85	110/80	90	19	36	95	75	250	4	461	639	11	65	456	4	
70	81	100/60	73	22	35	94	77	257	5	278	321	7	55	476	4	
71	89	110/70	83	19	35	95	79	263	8	379	243	7	68	421	4	
72	76	115/70	85	18	35	96	85	283	6	379	459	8	21	476	5	
73	130	110/70	83	38	33	86	55	79	29	1898	1376	27	236	997	34	
74	89	110/70	83	19	35	96	80	267	5	431	433	5	68	411	3	
75	85	110/80	90	19	36	95	75	250	4	329	639	11	65	456	4	
76	81	100/60	73	22	35	94	77	257	8	456	321	7	55	476	4	
77	89	110/70	83	19	35	95	79	263	8	379	243	7	68	421	4	
78	76	115/70	85	18	35	96	85	283	6	379	459	8	39	476	5	
79	81	100/60	73	22	35	94	77	257	5	578	321	7	55	476	4	
80	89	110/70	83	19	35	96	80	267	5	564	433	5	68	411	5	
81	131	110/70	83	38	28	84	56	80	31	1799	1336	21	231	1001	33	
82	89	110/70	83	19	35	96	80	267	5	564	433	8	68	411	3	
83	85	110/80	90	19	36	95	75	250	4	645	639	11	65	456	4	
84	81	100/60	73	22	35	94	77	257	15	345	321	7	55	476	4	
85	89	110/70	83	19	35	96	80	267	5	412	433	5	68	411	3	
86	85	110/80	90	19	36	95	75	250	4	456	639	11	65	456	4	
87	81	100/60	73	22	35	94	77	257	5	578	321	7	55	476	4	
88	89	110/70	83	19	35	95	79	263	8	379	243	7	68	421	4	
89	76	115/70	85	18	35	96	85	283	6	379	459	8	42	476	5	

90	89	110/70	83	19	35	96	80	267	5	564	433	9	68	411	5	
91	85	110/80	90	19	36	95	75	300	18	478	639	11	65	456	4	
92	81	100/60	73	22	35	94	77	257	5	578	321	7	55	476	4	
93	89	110/70	83	19	35	95	79	263	8	379	243	7	68	421	4	
94	76	115/70	85	18	35	96	85	283	6	379	459	8	21	476	5	
95	89	110/70	83	19	35	96	80	267	5	311	433	5	68	411	3	
96	85	110/80	90	19	36	95	75	250	4	578	639	11	65	456	4	
97	81	100/60	73	22	35	94	77	257	12	432	321	7	55	476	4	
98	89	110/70	83	19	35	96	80	267	5	446	433	5	68	411	3	
99	85	110/80	90	19	36	95	75	250	4	421	639	11	65	456	4	13.34
100	81	100/60	73	22	35	94	77	257	5	578	321	7	55	476	4	3.81
101	89	110/70	83	19	35	95	79	263	8	379	243	7	68	421	4	74.90
102	76	115/70	85	18	35	96	85	283	7	379	459	8	21	476	5	
103	87	115/90	98	21	35	96	75	250	6	587	378	9	65	455	4	
104	89	110/70	83	19	35	95	79	263	8	379	243	7	68	421	4	
105	124	100/70	80	38	36	85	55	79	30	1789	1335	35	335	991	34	
106	89	110/70	83	19	35	96	80	267	5	400	433	5	48	411	3	
107	85	110/80	90	19	36	95	75	250	9	499	639	11	65	456	4	
108	81	100/60	73	22	35	94	77	257	5	419	321	7	55	476	4	123.30
109	89	110/70	83	19	35	96	80	267	5	415	433	5	68	411	5	
110	85	110/80	90	19	36	95	75	250	4	349	639	11	65	456	4	
111	81	100/60	73	22	35	94	77	257	5	478	321	7	55	476	4	
112	89	110/70	83	19	35	95	79	263	8	379	243	7	68	421	4	
113	90	115/70	85	18	35	96	85	283	8	379	459	8	49	476	5	150.30
114	89	110/70	83	19	35	96	80	267	5	467	433	9	68	411	3	
115	85	110/80	90	19	36	95	75	250	9	432	639	11	65	456	4	
116	81	100/60	73	22	35	94	77	257	15	327	321	7	55	476	4	
117	89	110/70	83	19	35	95	79	263	8	379	243	7	68	421	4	
118	90	115/70	85	18	35	96	85	283	9	379	459	8	59	476	5	68.80
119	76	120/80	93	22	36	95	79	263	7	245	589	4	69	432	4	
120	89	110/70	83	19	35	96	80	267	5	467	433	5	68	411	3	
121	85	110/80	90	19	36	95	75	250	8	571	639	11	65	456	4	
122	81	100/60	73	22	35	94	77	257	8	312	321	7	55	321	4	

123	89	110/70	83	19	35	95	79	263	8	379	243	7	68	421	4	
124	76	115/70	85	18	35	96	85	283	8	379	459	8	46	476	5	
125	76	120/80	93	22	36	95	79	300	7	453	589	4	69	453	4	

**Jusvinza**	**7d**														
**Código**	**FC**	**PA**	**PAM**	**FR**	**Temp**	**SO_2**	**PO_2**	**P/F**	**INL**	**LDH**	**Ferritina**	**PCR**	**Eritro**	**Fibrinógeno**	**DD**
1	78	110/70	83	19	35	96	80	267	5	564	433	5	68	411	3
2	76	110/80	90	19	36	95	75	250	4	453	639	11	65	456	5
3	87	100/60	73	22	35	94	77	257	5	578	321	7	55	476	4
4	89	110/70	83	19	35	95	79	290	8	379	243	7	68	421	5
5	76	115/70	85	18	35	96	85	283	8	379	459	8	21	476	5
6	135	110/70	83	38	34	82	66	94	31	1945	1215	24	238	986	33
7	89	120/70	86	22	36	94	88	293	6	439	421	6	77	388	4
8	85	120/80	93	22	36	95	79	263	7	659	589	4	69	460	4
9	81	115/90	98	21	35	96	75	250	9	587	378	5	65	455	4
10	92	115/90	98	21	35	96	75	250	9	587	378	5	65	455	4
11	78	110/70	83	19	35	96	80	267	5	564	433	5	68	411	3
12	76	110/80	90	19	36	95	75	250	4	516	639	11	65	456	4
13	87	100/60	73	22	35	94	77	257	5	578	321	7	55	476	4
14	114	110/70	83	38	36	84	55	79	39	1956	1345	33	232	987	32
15	89	120/70	86	22	36	94	88	293	6	439	421	6	65	388	2
16	85	120/80	93	22	36	95	79	263	7	453	589	4	69	560	2
17	81	115/90	98	21	35	96	75	250	6	587	378	5	65	455	2
18	89	110/70	83	19	35	95	79	263	8	379	243	7	68	421	4
19	76	115/70	85	18	35	96	85	283	7	379	459	8	21	476	5
20	122	100/70	80	38	35	88	55	79	29	1898	1424	31	234	928	32
21	89	120/70	86	22	36	94	88	293	6	439	421	6	65	388	5
22	85	120/80	93	22	36	95	79	263	7	659	589	4	76	464	5
23	81	115/90	98	21	35	96	75	250	9	587	378	5	65	455	2
24	89	110/70	83	19	35	95	79	263	8	379	243	7	68	421	4
25	76	115/70	85	18	35	96	85	283	8	379	459	8	56	476	5
26	117	110/70	83	38	28	84	65	93	29	1779	º321	33	132	994	30

27	78	110/70	83	19	35	96	80	267	5	564	433	5	68	411	3
28	76	110/80	90	19	36	95	75	250	4	645	639	11	65	456	4
29	87	100/60	73	22	35	94	77	257	5	578	321	7	55	476	4
30	89	110/70	83	19	35	95	79	263	8	379	243	7	68	421	4
31	76	115/70	85	18	35	96	85	283	8	379	459	8	21	476	5
32	111	100/70	80	38	34	84	55	79	30	1759	1375	30	229	993	32
33	89	120/70	86	22	36	94	88	293	6	439	421	6	65	388	5
34	85	120/80	93	22	36	95	79	263	7	659	589	4	76	464	5
35	81	115/90	98	21	35	96	75	250	9	587	378	5	65	455	2
36	89	110/70	83	19	35	95	79	263	8	379	243	7	68	421	4
37	76	115/70	85	18	35	96	85	283	8	379	459	8	56	476	5
38	89	110/70	83	19	35	96	80	267	5	564	433	5	68	411	5
39	85	110/80	90	19	36	95	75	250	4	578	639	11	65	456	4
40	81	100/60	73	22	35	94	77	257	5	578	321	7	55	476	4
41	121	110/70	83	38	34	86	58	83	30	1789	1368	33	232	877	30
42	85	100/60	73	22	35	94	77	257	5	578	321	7	55	476	4
43	81	110/70	83	19	35	96	80	267	5	315	433	5	68	411	3
44	117	110/70	83	38	3º	84	55	79	27	1864	1415	33	231	983	28
45	81	110/80	90	19	36	95	75	250	4	645	639	11	65	456	4
46	89	100/60	73	22	35	94	77	257	7	578	321	7	55	476	4
47	81	100/60	73	22	35	94	77	257	5	278	321	7	55	476	4
48	119	110/70	83	38	33	85	53	76	29	1993	1388	32	232	985	30
49	89	110/70	83	19	35	96	80	267	5	564	433	5	68	411	3
50	85	110/80	90	19	36	95	75	250	4	326	639	11	65	456	4
51	81	100/60	73	22	35	94	77	257	5	418	321	7	55	476	4
52	89	110/70	83	19	35	95	79	263	8	379	243	7	68	421	4
53	76	115/70	85	18	35	96	85	283	9	379	459	8	45	476	5
54	81	120/80	93	22	36	95	79	263	7	435	589	4	69	560	4
55	89	110/70	83	19	35	96	80	267	5	399	433	5	68	411	5
56	89	110/80	90	19	36	95	75	250	4	461	639	11	65	456	4
57	85	100/60	73	22	35	94	77	257	5	278	321	7	55	476	4
58	81	110/70	83	19	35	95	79	263	8	379	243	7	68	421	4
59	89	115/70	85	18	35	96	85	283	6	379	459	8	21	476	5

60	76	115/70	85	18	35	96	85	283	6	379	459	8	39	476	5
61	135	110/70	83	38	33	86	55	79	29	1898	1376	27	236	997	34
62	89	110/70	83	19	35	96	80	267	5	453	433	5	68	411	3
63	85	110/80	90	19	36	95	75	250	4	645	639	11	65	456	4
64	81	100/60	73	22	35	94	77	257	9	578	321	7	55	476	4
65	89	110/70	83	19	35	95	79	263	8	379	243	7	68	421	4
66	76	115/70	85	18	35	96	85	283	7	379	459	8	21	476	5
67	76	100/60	73	22	35	94	77	257	5	578	321	7	55	476	4
68	89	110/70	83	19	35	96	80	267	5	564	433	5	68	411	3
69	85	110/70	83	19	35	96	80	267	5	431	433	5	68	411	5
70	81	110/80	90	19	36	95	75	250	4	564	639	11	65	456	4
71	89	100/60	73	22	35	94	77	257	5	578	321	7	55	476	4
72	76	110/70	83	19	35	95	79	263	8	379	243	7	68	421	4
73	130	100/70	80	38	36	87	51	73	25	1882	1387	29	239	999	31
74	89	110/70	83	19	35	96	80	267	5	564	433	8	68	411	3
75	85	110/80	90	19	36	95	75	250	4	645	639	11	65	456	4
76	81	100/60	73	22	35	94	77	257	15	345	321	7	55	476	4
77	89	110/70	83	19	35	96	80	267	5	412	433	5	68	411	3
78	76	110/80	90	19	36	95	75	250	4	456	639	11	65	456	4
79	81	100/60	73	22	35	94	77	257	5	578	321	7	55	476	4
80	89	110/70	83	19	35	95	79	263	8	379	243	7	68	421	4
81	131	100/70	80	38	36	85	55	79	30	1789	1335	35	335	991	34
82	89	110/70	83	19	35	96	80	267	5	431	433	5	68	411	3
83	85	110/80	90	19	36	95	75	250	4	329	639	11	65	456	4
84	81	100/60	73	22	35	94	77	257	8	456	321	7	55	476	4
85	89	110/70	83	19	35	95	79	263	8	379	243	7	68	421	4
86	85	115/70	85	18	35	96	85	283	6	379	459	8	39	476	5
87	81	100/60	73	22	35	94	77	257	5	578	321	7	55	476	4
88	89	110/70	83	19	35	96	80	267	5	564	433	5	68	411	5
89	76	110/70	83	19	35	96	80	267	5	400	433	5	48	411	3
90	89	110/80	90	19	36	95	75	250	9	499	639	11	65	456	4
91	85	100/60	73	22	35	94	77	257	5	419	321	7	55	476	4
92	81	110/70	83	19	35	96	80	267	5	415	433	5	68	411	5

93	89	110/80	90	19	36	95	75	250	4	349	639	11	65	456	4
94	76	100/60	73	22	35	94	77	257	5	478	321	7	55	476	4
95	89	110/70	83	19	35	95	79	263	8	379	243	7	68	421	4
96	85	115/70	85	18	35	96	85	283	8	379	459	8	49	476	5
97	81	110/70	83	19	35	96	80	267	5	467	433	9	68	411	3
98	89	110/80	90	19	36	95	75	250	9	432	639	11	65	456	4
99	85	100/60	73	22	35	94	77	257	15	327	321	7	55	476	4
100	81	110/70	83	19	35	95	79	263	8	379	243	7	68	421	4
101	89	115/70	85	18	35	96	85	283	9	379	459	8	59	476	5
102	76	120/80	93	22	36	95	79	263	7	245	589	4	69	432	4
103	87	110/70	83	19	35	96	80	267	5	467	433	5	68	411	3
104	89	110/80	90	19	36	95	75	250	8	571	639	11	65	456	4
105	124	110/70	83	38	28	84	56	80	31	1799	1336	21	231	1001	33
106	89	115/70	85	18	35	96	85	283	9	379	459	8	59	476	5
107	85	120/80	93	22	36	95	79	263	7	245	589	4	69	432	4
108	81	110/70	83	19	35	96	80	267	5	467	433	5	68	411	3
109	89	110/80	90	19	36	95	75	250	8	571	639	11	65	456	4
110	85	100/60	73	22	35	94	77	257	8	312	321	7	55	321	4
111	81	110/70	83	19	35	95	79	263	8	379	243	7	68	421	4
112	89	115/70	85	18	35	96	85	283	8	379	459	8	46	476	5
113	90	120/80	93	22	36	95	79	300	7	453	589	4	69	453	4
114	89	110/70	83	19	35	96	80	267	5	400	433	5	48	411	3
115	85	110/80	90	19	36	95	75	250	9	499	639	11	65	456	4
116	81	100/60	73	22	35	94	77	257	5	419	321	7	55	476	4
117	89	110/70	83	19	35	96	80	267	5	415	433	5	68	411	5
118	90	110/80	90	19	36	95	75	250	4	349	639	11	65	456	4
119	76	100/60	73	22	35	94	77	257	5	478	321	7	55	476	4
120	89	110/70	83	19	35	95	79	263	8	379	243	7	68	421	4
121	85	115/70	85	18	35	96	85	283	8	379	459	8	49	476	5
122	81	110/70	83	19	35	96	80	267	5	467	433	9	68	411	3
123	89	110/80	90	19	36	95	75	250	9	432	639	11	65	456	4
124	76	100/60	73	22	35	94	77	257	15	327	321	7	55	476	4
125	76	110/70	83	19	35	95	79	263	8	379	243	7	68	421	4

No Jusvinza								Antes de comenzar el tratamiento															
Código	Desenlace	F.Ingreso	Final tto	Edad	Raza	IMC	APP	FC	PA	PAM	FR	Temp	SO_2	PO_2	P/F	INL	LDH	Ferritina	PCR	Eritro	Fibrinógeno	DD	IL-6
1	V	4.4.21	3.5.21	89	B	O	HTA, AB	99	90/60	63	36	38	85	50	167	21	1000	1324	25	122	897	15	
2	V	21.3.21	17.4.21	59	B	NP	DM,HTA	120	80/40	53	38	38	82	49	163	21	989	990	15	110	879	9	
3	V	25.4.21	24.5.21	77	B	O	HTA	115	110/60	76	35	35	87	60	200	22	1421	1234	25	112	763	8	
4	F	1.5.21	19.5.21	87	M	O	AB	99	90/60	63	40	36	88	55	183	15	1256	1543	28	167	875	8	14.7
5	F	22.3.21	7.4.21	67	B	O	DM,HTA	111	100/70	80	41	38	85	60	200	16	2000	1678	26	158	897	7	
6	V	17.5.21	10.6.21	63	M	NP	HTA,CI	99	110/60	76	40	35	90	61	203	19	1200	1456	28	154	871	11	
7	F	21.5.21	10.6.21	87	M	O	HTA,CI	99	90/60	63	40	36	88	55	183	15	1256	1543	28	167	875	8	
8	F	26.5.21	10.6.21	70	B	NP	HTA,CI	109	110/70	83	29	38	87	55	183	8	1457	798	22	99	893	11	
9	F	28.4.21	8.5.21	62	M	O	HTA,CI	104	110/70	83	33	38	89	56	187	19	1266	1221	25	165	978	9	
10	V	19.5.21	15.6.21	87	M	NP	HTA,CI	99	90/60	63	40	36	88	55	183	15	1256	1543	28	167	875	8	
11	V	12.5.21	4.6.21	67	B	O	DM,HTA	111	100/70	80	41	38	85	60	200	16	2000	1678	26	158	897	7	
12	V	30.5.21	16.6.21	69	B	SP	DM	122	80/60	66	30	38	86	55	183	22	989	768	19	101	798	12	
13	F	19.5.21	31.5.21	62	M	O	DM,HTA	104	110/70	83	33	38	89	56	187	19	1266	1221	25	98	978	9	
14	V	7.5.21	4.6.21	87	M	SP	DM,HTA	122	90/60	63	40	36	88	55	183	15	1256	1543	28	167	875	8	489.2
15	F	29.5.21	9.6.21	70	B	NP	HTA,CI	109	110/70	83	29	38	87	55	183	8	1543	798	22	99	893	11	
16	V	22.4.21	20.5.21	69	B	SP	HTA,CI	135	80/60	66	30	38	86	55	183	13	745	768	19	101	798	12	
17	F	22.5.21	3.6.21	70	B	NP	HTA,CI	109	110/70	83	29	38	87	55	183	8	786	798	22	99	893	11	
18	F	27.4.21	13.5.21	87	M	BP	HTA,CI	135	90/60	63	40	36	88	55	183	15	1256	1543	28	79	875	8	
19	V	8.5.21	4.6.21	67	B	BP	DM,HTA	111	100/70	80	41	38	85	60	200	16	2000	1678	26	158	897	7	70.1
20	F	21.3.21	6.4.21	70	B	NP	HTA,CI	109	110/70	83	29	38	87	55	183	8	786	798	22	99	893	11	
21	V	21.3.21	12.4.21	69	B	SP	DM	123	80/60	66	30	38	86	55	183	13	745	768	19	101	798	12	
22	F	26.4.21	13.5.21	62	M	O	EPOC	104	110/70	83	33	38	89	56	187	19	1266	1221	25	165	978	9	
23	V	29.5.21	28.6.21	69	B	SP	GASTRITIS	122	80/60	66	30	38	86	55	183	13	745	768	19	101	798	12	
24	F	21.3.21	9.4.21	87	M	O	HTA,CI	99	90/60	63	40	36	88	55	183	15	1256	1543	28	167	875	8	
25	F	21.3.21	7.4.21	67	B	O	DM,HTA	111	100/70	80	41	38	85	60	200	16	2000	1678	26	94	897	7	
26	V	24.3.21	18.4.21	70	B	NP	HTA,CI	109	110/70	83	29	38	87	55	183	8	786	798	22	99	893	11	
27	F	22.3.21	10.4.21	69	B	SP	DM	128	80/60	66	30	38	86	55	183	13	745	768	19	101	798	12	
28	F	26.3.21	10.4.21	62	M	O	HTA,CI	104	110/70	83	33	38	89	56	187	19	1266	1221	25	165	978	9	

29	F	30.3.21	15.4.21	87	M	O	HTA, AB	133	90/60	63	40	36	88	55	183	15	1256	1543	28	95	875	16	
30	V	21.3.21	16.4.21	67	B	SP	DM,HTA	111	100/70	80	41	38	85	60	200	16	2000	1678	26	158	897	15	
31	F	28.3.21	14.4.21	70	B	NP	AB	109	110/70	83	38	38	87	55	183	22	786	798	22	99	893	11	
32	F	23.3.21	11.4.21	69	B	SP	HTA, AB	129	80/60	66	30	38	86	55	183	13	745	768	19	101	798	12	
33	V	27.3.21	8.4.21	62	M	O	HTA, AB	104	110/70	83	33	38	89	56	187	19	1266	1221	25	165	978	9	
34	F	21.3.21	4.4.21	87	M	O	HTA, AB	99	90/60	63	40	36	88	55	183	15	1256	1543	28	167	875	8	
35	F	24.3.21	6.4.21	70	B	NP	HTA, AB	109	110/70	83	38	38	87	55	183	8	786	798	22	99	893	11	
36	V	25.3.21	17.4.21	69	B	SP	DM	132	80/60	66	30	38	86	55	183	22	745	768	19	101	798	12	
37	F	28.3.21	12.4.21	87	M	O	HTA	154	90/60	63	40	36	88	55	183	15	1256	1543	28	78	875	8	
38	F	24.3.21	10.4.21	70	B	NP	AB	109	110/70	83	36	38	87	55	183	8	786	798	22	99	893	11	
39	F	31.3.21	19.4.21	69	B	SP	DM	78	80/60	66	30	38	86	55	183	13	745	768	19	101	798	12	
40	V	22.3.21	13.4.21	70	B	NP	HTA	109	110/70	83	39	38	87	55	183	22	786	798	22	99	893	11	
41	F	27.3.21	9.4.21	69	B	SP	HTA	78	80/60	66	30	38	86	55	183	22	745	768	19	101	798	12	
42	F	25.3.21	10.4.21	62	M	O	HTA	104	110/70	83	33	38	89	56	187	19	1266	1221	25	79	978	9	
43	V	21.3.21	7.4.21	69	B	SP	DM	135	80/60	66	30	38	86	55	183	13	745	768	19	101	798	12	
44	F	22.3.21	7.4.21	87	M	O	HTA	111	90/60	63	40	36	88	55	183	15	1256	1543	28	167	875	8	
45	F	24.3.21	4.4.21	67	B	SP	DM,HTA	132	100/70	80	41	38	85	60	200	16	2000	1678	26	158	897	7	
46	V	1.4.21	23.4.21	70	B	NP	HTA	126	110/70	83	29	38	87	55	183	8	786	798	22	99	893	11	
47	F	20.4.21	4.5.21	69	B	SP	DM	78	80/60	66	30	38	86	55	183	13	745	768	19	101	798	12	
48	F	2.4.21	21.4.21	62	M	O	EPOC	104	110/70	83	33	38	89	56	187	19	1266	1221	25	99	978	9	
49	F	5.4.21	22.4.21	87	M	BP	HTA	132	90/60	63	40	36	88	55	183	15	1256	1543	28	167	875	8	
50	V	28.3.21	14.4.21	67	B	NP	DM,HTA	128	100/70	80	41	38	85	60	200	16	2000	1678	26	79	897	18	
51	F	26.4.21	15.5.21	69	B	SP	HTA	78	80/60	66	30	38	86	55	183	13	745	768	19	101	798	12	
52	V	11.4.21	9.5.21	62	M	O	HTA	104	110/70	83	33	38	89	56	187	19	1266	1221	25	165	978	9	
53	V	17.4.21	4.5.21	87	M	O	HTA	122	90/60	63	40	36	88	55	183	15	1256	1543	28	167	875	8	
54	F	22.4.21	8.5.21	67	B	O	DM,HTA	111	100/70	80	41	38	85	60	200	16	2000	1678	26	158	897	7	
55	F	21.3.21	02.4.21	69	B	SP	DM	78	80/60	66	30	38	86	55	183	22	1123	768	19	101	798	12	
56	V	11.6.21	26.6.21	62	M	O	HTA	128	110/70	83	33	38	89	56	187	19	1266	1221	25	88	978	9	
57	F	30.5.21	18.6.21	87	M	BP	HTA	122	90/60	63	40	36	88	55	183	15	1256	1543	28	167	875	16	
58	F	6.6.21	23.6.21	67	B	SP	DM,HTA	111	100/70	80	41	38	85	60	200	16	2000	1678	26	158	897	15	
59	F	25.3.21	10.4.21	69	B	SP	DM	121	80/60	66	30	38	86	55	183	13	745	768	19	101	798	12	
60	V	1.6.21	16.6.21	62	M	O	HTA	104	110/70	83	33	38	89	56	187	19	1266	1221	25	165	978	14	
61	F	21.3.21	9.4.21	87	M	SP	HTA	129	90/60	63	40	36	88	55	183	15	1256	1543	28	167	875	8	

62	F	20.5.21	4.6.21	67	B	O	DM,HTA	111	100/70	80	41	38	85	60	200	16	2000	1678	26	158	897	7	
63	V	22.4.21	13.5.21	69	B	SP	HTA	126	80/60	66	30	38	86	55	183	13	987	768	19	101	798	12	
64	F	2.6.21	18.6.21	62	M	O	EPOC	104	110/70	83	33	38	89	56	187	19	1266	1221	25	165	978	9	
65	F	27.4.21	15.5.21	87	M	O	AB	145	90/60	63	40	36	88	55	183	15	1256	1543	28	167	875	8	
66	V	22.5.21	7.6.21	67	B	SP	DM,HTA	111	100/70	80	41	38	85	60	200	16	2000	1678	26	158	897	22	
67	F	5.4.21	24.4.21	69	B	SP	DM	165	80/60	66	30	38	86	55	183	13	745	768	19	101	798	12	
68	F	9.6.21	25.6.21	62	M	O	HTA	104	110/70	83	33	38	89	56	187	19	1266	1221	25	165	978	22	
69	F	29.3.21	14.4.21	87	M	BP	HTA	99	90/60	63	40	36	88	55	183	15	1256	1543	28	167	875	19	
70	V	30.5.21	12.6.21	67	B	O	DM,HTA	111	100/70	80	41	38	85	60	200	16	2000	1678	26	158	897	22	
71	F	18.4.21	7.5.21	69	B	SP	DM	78	80/60	66	30	38	86	55	183	13	745	768	19	101	798	12	
72	V	11.6.21	27.6.21	62	M	O	HTA	133	110/70	83	33	38	89	56	187	19	1266	1221	25	98	978	9	
73	F	21.3.21	5.4.21	87	M	SP	HTA	99	90/60	63	40	36	88	55	183	15	1256	1543	28	167	875	8	
74	F	22.5.21	9.6.21	67	B	O	DM,HTA	111	100/70	80	41	38	85	60	200	16	2000	1678	26	158	897	7	
75	F	4.6.21	23.6.21	70	B	NP	HTA	109	110/70	83	37	38	87	55	183	8	786	798	22	99	893	11	
76	V	6.4.21	27.4.21	69	B	SP	HTA	127	80/60	66	30	38	86	55	183	13	745	768	19	101	798	12	
77	F	21.5.21	9.6.21	62	M	O	HTA	104	110/70	83	33	38	89	56	187	19	1266	1221	25	165	978	19	
78	F	29.3.21	12.4.21	87	M	O	HTA	134	90/60	63	40	36	88	55	183	15	1256	1543	28	167	875	8	
79	F	18.6.21	30.6.21	67	B	BP	DM,HTA	111	100/70	80	41	38	85	60	200	16	2000	1678	26	158	897	22	
80	V	22.4.21	17.5.21	69	B	SP	DM	122	80/60	66	30	38	86	55	183	22	745	768	19	101	798	12	
81	V	26.6.21	17.7.21	62	M	O	EPOC	104	110/70	83	33	38	89	56	187	19	1266	1221	25	165	978	9	
82	F	28.6.21	17.7.21	87	M	O	HTA	99	90/60	63	40	36	88	55	183	15	1256	1543	28	167	875	22	
83	V	29.6.21	24.7.21	67	B	SP	DM,HTA	111	100/70	80	41	38	85	60	200	16	2000	1678	26	158	897	19	
84	F	24.5.21	9.6.21	69	B	SP	HTA	78	80/60	66	30	38	86	55	183	13	745	768	19	101	798	12	
85	V	19.4.21	9.5.21	62	M	O	HTA	114	110/70	83	33	38	89	56	187	19	1266	1221	25	165	978	28	
86	V	30.6.21	21.7.21	70	B	NP	HTA	112	110/70	83	38	38	87	55	183	22	786	798	22	99	893	11	
87	F	15.5.21	3.6.21	69	B	SP	DM	78	80/60	66	30	38	86	55	183	13	745	768	19	101	798	12	9.4
88	F	12.5.21	4.6.21	62	M	O	HTA	104	110/70	83	33	38	89	56	187	19	1266	1221	25	165	978	16	12,9
89	F	2.5.21	19.5.21	87	M	O	HTA	99	90/60	63	40	36	88	55	183	15	1256	1543	28	167	875	12	401
90	V	21.3.21	4.4.21	67	B	SP	DM,HTA	128	100/70	80	41	38	85	60	200	16	2000	1678	26	158	897	12	
91	F	30.6.21	19.7.21	69	B	SP	HTA	78	80/60	66	30	38	86	55	183	13	745	768	19	101	798	12	
92	F	19.4.21	4.5.21	62	M	O	HTA	113	110/70	83	33	38	89	56	187	19	1266	1221	25	165	978	15	
93	V	28.5.21	23.6.21	87	M	BP	AB	128	90/60	63	40	36	88	55	183	15	1256	1543	28	167	875	16	
94	F	29.5.21	17.6.21	67	B	SP	DM,HTA	113	100/70	80	41	38	85	60	200	16	2000	1678	26	158	897	15	

95	F	12.4.21	25.4.21	69	B	SP	HTA	78	80/60	66	30	38	86	55	183	13	745	768	19	101	798	12	
96	V	19.5.21	8.6.21	62	M	O	HTA	104	110/70	83	33	38	89	56	187	19	1266	1221	25	165	978	15	
97	F	28.3.21	15.4.21	87	M	O	HTA	99	90/60	63	40	36	88	55	183	15	1256	1543	28	167	875	18	
98	F	7.7.21	26.7.21	67	B	SP	DM,HTA	111	100/70	80	41	38	85	60	200	16	2000	1678	26	158	897	16	
99	F	30.5.21	19.6.21	69	B	SP	HTA	136	80/60	66	30	38	86	55	183	13	979	768	19	101	798	12	
100	V	22.3.21	19.4.21	62	M	O	HTA	104	110/70	83	33	38	89	56	187	19	1266	1221	25	165	978	14	
101	F	15.5.21	27.5.21	87	M	NP	HTA	122	90/60	63	40	36	88	55	183	15	1256	1543	28	167	875	17	80.5
102	V	5.7.21	30.7.32	67	B	BP	DM,HTA	133	100/70	49	41	38	85	60	200	16	2000	1678	26	98	897	18	
103	V	18.4.21	12.5.21	69	B	SP	DM	122	80/60	66	30	38	86	55	183	22	745	768	19	101	798	12	
104	F	6.6.21	25.6.21	62	M	O	HTA	104	110/70	55	33	38	89	56	187	19	1266	1221	25	103	978	22	
105	F	9.6.21	30.6.21	62	M	O	HTA	113	110/70	54	33	38	89	56	187	19	1266	1221	25	111	978	16	
106	V	3.5.21	27.5.21	69	B	SP	HTA	135	80/60	66	30	38	86	55	183	22	745	768	19	101	798	12	11.6
107	F	6.6.21	5.7.21	87	M	O	HTA	99	90/60	55	40	36	88	55	183	22	1256	1543	28	167	875	18	
108	F	30.5.21	18.6.21	67	B	NP	DM,HTA	125	100/70	80	41	38	85	60	200	16	2000	1678	26	121	897	17	
109	F	10.6.21	5.7.21	69	B	SP	DM	156	80/60	66	30	38	86	55	183	13	1476	768	19	101	798	12	
110	V	30.5.21	21.6.21	62	M	O	EPOC	129	110/70	83	33	38	89	56	187	22	1266	1221	25	99	978	22	
111	V	22.4.21	17.5.21	87	M	SP	HTA	122	90/60	63	40	36	88	55	183	22	1256	1543	28	167	875	17	
112	F	21.3.21	10.5.21	67	B	O	DM,HTA	111	100/70	55	41	38	85	60	200	16	2000	1678	26	158	897	21	
113	V	18.6.21	10.7.21	69	B	SP	HTA	128	80/60	66	30	38	86	55	183	27	1239	768	19	101	798	12	
114	F	28.3.21	27.4.21	62	M	O	AB	104	110/70	83	33	38	89	56	187	19	1266	1221	25	165	777	17	
115	F	30.4.21	27.5.21	87	M	SP	GASTRITIS	99	90/60	63	40	36	88	55	183	15	1256	1543	28	167	875	18	
116	V	15.4.21	10.5.21	67	B	O	DM,HTA	149	100/70	80	41	38	85	60	200	22	1289	1678	26	111	488	18	
117	F	24.3.21	7.4.21	70	B	NP	GASTRITIS	113	110/70	83	44	38	87	55	183	22	1578	798	22	99	689	11	
118	F	11.4.21	18.4.21	69	B	SP	GASTRITIS	127	80/60	66	30	38	86	55	183	13	1231	768	19	101	798	12	
119	F	29.4.21	18.5.21	62	M	O	GASTRITIS	114	110/70	83	33	38	89	56	187	19	1266	1221	25	165	555	16	
120	V	13.4.21	12.5.21	89	B	O	GASTRITIS	122	90/60	63	36	38	85	50	167	21	1000	1324	25	122	897	15	
121	V	20.4.21	20.5.21	59	B	NP	DM,HTA	120	80/40	53	38	38	82	49	163	22	1237	990	15	110	777	19	
122	V	6.4.21	1.5.21	77	B	O	GASTRITIS	115	110/60	76	35	35	87	60	200	22	1421	1234	25	112	763	18	
123	V	1.4.21	26.4.21	69	B	SP		120	80/60	55	30	38	86	55	183	25	1236	768	19	101	798	12	
124	F	4.4.21	23.4.21	70	B	NP		145	110/70	66	36	38	87	55	183	22	1755	798	22	99	893	11	

No Jusvinza	48h														
Código	FC	PA	PAM	FR	Temp	SO_2	PO_2	P/F	INL	LDH	Ferritina	PCR	Eritro	Fibrinógeno	DD
1	110	90/60	63	37	37	85	62	89	34	1254	1452	11	134	899	17
2	130	90/60	63	38	38	80	60	200	19	995	998	18	121	915	14
3	117	90/60	63	40	38	82	60	200	24	1479	1346	19	130	895	12
4	115	90/60	63	40	38	88	55	111	21	1465	1322	19	221	894	11
5	116	100/60	73	41	37	89	55	183	19	1389	1788	28	200	911	12
6	110	90/60	63	40	35	92	57	190	22	1342	1687	31	198	912	15
7	115	90/60	63	40	38	88	55	79	21	1565	1897	32	221	894	11
8	115	90/60	63	38	37	82	69	99	26	1257	1221	24	126	919	13
9	122	100/60	73	34	37	85	55	69	21	1547	1431	29	221	998	18
10	115	90/60	63	40	38	88	55	79	21	1565	1897	32	221	894	11
11	116	100/60	73	41	37	89	55	183	19	1565	1788	28	165	911	12
12	125	80/60	66	29	37	90	79	132	25	985	1354	26	130	987	22
13	122	100/60	73	34	37	85	55	69	21	1432	1431	29	221	998	18
14	115	90/60	63	40	38	88	55	121	21	1565	1897	32	189	894	11
15	115	90/60	63	38	37	82	69	99	26	1567	1221	24	126	919	13
16	125	80/60	66	29	37	90	79	132	25	985	1354	26	130	987	22
17	115	90/60	63	38	37	82	69	99	26	1567	1221	24	126	919	13
18	115	90/60	63	40	38	88	55	79	21	1565	1897	32	221	894	11
19	116	100/60	73	41	37	89	55	183	19	1231	1788	19	179	911	12
20	115	90/60	63	38	37	82	69	99	26	1567	1221	24	126	919	13
21	125	80/60	66	29	37	90	79	132	25	985	1354	26	130	987	22
22	122	100/60	73	34	37	85	55	69	21	1547	1431	29	221	998	18
23	125	80/60	66	29	37	90	79	132	25	985	1354	26	130	987	22
24	115	90/60	63	40	38	88	55	79	21	1565	1897	32	221	894	11
25	116	100/60	73	41	37	89	55	183	19	1678	689	28	200	911	12
26	115	90/60	63	38	37	82	69	118	26	1567	1221	24	99	919	13
27	125	80/60	66	29	37	90	79	132	25	985	1354	26	130	987	22
28	122	100/60	73	34	37	85	55	111	21	1547	1431	29	221	998	18
29	115	90/60	63	40	38	88	55	79	21	1565	879	32	221	894	11
30	116	100/60	73	41	37	89	55	183	19	1468	999	28	89	911	12

31	115	90/60	63	38	37	82	69	99	26	1567	1221	24	126	919	13
32	125	80/60	66	29	37	90	79	132	25	985	1354	26	130	987	22
33	122	100/60	73	34	37	85	55	168	21	1547	1431	29	189	998	18
34	115	90/60	63	40	38	88	55	79	21	1565	1897	32	221	894	11
35	115	90/60	63	38	37	82	69	111	26	1567	1221	24	126	919	13
36	125	80/60	66	29	37	90	79	132	25	985	1354	26	130	987	22
37	115	90/60	63	40	38	88	55	79	21	1565	1279	32	221	894	11
38	115	90/60	63	38	37	82	69	99	26	1567	1221	24	126	919	13
39	125	80/60	66	29	37	90	79	132	25	985	1354	26	130	987	22
40	115	90/60	63	38	37	82	69	99	26	1567	1221	24	126	919	13
41	125	80/60	66	29	37	90	79	132	25	985	1354	26	130	987	22
42	122	100/60	73	34	37	85	55	69	21	1547	1431	29	221	998	18
43	125	80/60	66	29	37	90	79	132	25	985	1354	26	111	987	22
44	115	90/60	63	40	38	88	55	79	21	1565	998	32	221	894	11
45	116	100/60	73	41	37	89	55	183	19	1678	1788	28	200	911	12
46	115	90/60	63	38	37	82	69	99	26	1567	1221	24	178	919	13
47	125	80/60	66	29	37	90	79	132	25	985	1354	26	130	987	22
48	122	100/60	73	34	37	85	55	123	21	1547	1431	29	221	998	18
49	115	90/60	63	40	38	88	55	79	21	1565	987	32	221	894	11
50	116	100/60	73	41	37	89	55	183	19	1678	1788	28	159	911	12
51	125	80/60	66	29	37	90	79	132	25	985	1354	26	130	987	22
52	122	100/60	73	34	37	85	55	132	21	1547	1431	29	221	998	18
53	115	90/60	63	40	38	88	55	127	21	1565	1897	32	221	894	11
54	116	100/60	73	41	37	89	55	183	19	1111	1788	28	200	911	12
55	125	80/60	66	29	37	90	79	132	25	985	1354	26	130	987	22
56	122	100/60	73	34	37	85	55	132	21	1547	1431	29	187	998	18
57	115	90/60	63	40	38	88	55	79	21	1565	1897	32	221	894	11
58	116	100/60	73	41	37	89	55	183	19	1479	1788	28	200	911	12
59	125	80/60	66	29	37	90	79	132	25	985	1354	26	130	987	22
60	122	100/60	73	34	37	85	55	69	21	1547	1431	29	178	998	18
61	115	90/60	63	40	38	88	55	79	21	1565	1897	32	221	894	27
62	116	100/60	73	41	37	89	55	183	19	1342	1788	28	200	911	12
63	125	80/60	66	29	37	90	79	132	25	985	1354	26	130	987	22

64	122	100/60	73	34	37	85	55	69	21	1547	1431	29	221	998	18
65	115	90/60	63	40	38	88	55	79	21	1565	1897	32	221	894	11
66	116	100/60	73	41	37	89	55	183	19	1654	1788	28	185	911	12
67	125	80/60	66	29	37	90	79	132	25	985	1354	26	130	987	22
68	122	100/60	73	34	37	85	55	69	21	1547	1431	29	221	998	18
69	115	90/60	63	40	38	88	55	79	21	1565	1897	32	221	894	29
70	116	100/60	73	41	37	89	55	183	19	1654	1788	28	111	911	12
71	125	80/60	66	29	37	90	79	132	25	985	1354	26	130	987	22
72	122	100/60	73	34	37	85	55	122	21	1547	1431	22	108	998	18
73	115	90/60	63	40	38	88	55	79	21	1565	1897	32	221	894	11
74	116	100/60	73	41	37	89	55	183	19	1344	1365	28	200	911	26
75	115	90/60	63	38	37	82	69	99	26	1567	1221	24	126	919	13
76	125	80/60	66	29	37	90	79	132	25	985	1354	26	130	987	22
77	122	100/60	73	34	37	85	55	111	21	1547	1431	29	221	998	18
78	115	90/60	63	40	38	88	55	126	21	1565	1232	32	221	894	11
79	116	100/60	73	41	37	89	55	183	19	1565	1788	28	200	911	12
80	125	80/60	66	29	37	90	79	132	25	985	1354	26	130	987	22
81	122	100/60	73	34	37	85	55	143	21	1547	1431	22	221	998	18
82	115	90/60	63	40	38	88	55	79	21	1565	1897	32	221	894	11
83	116	100/60	73	41	37	89	55	183	19	1675	1788	28	109	911	12
84	125	80/60	66	39	37	90	66	132	25	985	1354	26	130	987	22
85	122	100/60	73	34	37	85	55	147	21	1547	1431	22	106	998	18
86	115	90/60	63	38	37	82	69	125	26	1567	1221	24	126	919	13
87	125	80/60	66	29	37	90	79	132	25	985	1354	26	130	987	22
88	122	100/60	73	34	37	85	55	69	21	1547	1431	29	221	998	18
89	115	90/60	63	40	38	88	55	79	21	1565	1897	32	221	894	11
90	116	100/60	73	41	37	89	55	183	19	1351	1788	28	121	911	12
91	125	80/60	55	38	37	90	79	129	25	985	1354	26	130	987	22
92	122	100/60	73	34	37	85	55	132	21	1547	1431	29	221	998	18
93	115	90/60	63	40	38	88	55	156	21	1565	1897	22	189	894	11
94	116	100/60	73	41	37	89	55	183	19	1876	1788	28	200	911	12
95	125	80/60	66	29	37	90	79	132	25	985	1354	26	130	987	22
96	122	100/60	73	34	37	85	55	167	21	1547	1431	19	187	998	18

97	115	90/60	63	40	38	88	55	79	21	1565	897	32	221	894	11
98	116	100/60	73	41	37	89	55	183	19	1254	1788	28	99	911	12
99	125	80/60	66	29	37	90	55	132	25	985	1354	26	130	987	22
100	122	100/60	73	34	37	85	55	123	21	1547	1431	22	176	998	18
101	115	90/60	63	40	38	88	55	79	21	1565	854	32	99	894	11
102	116	100/60	55	41	37	89	55	183	19	1342	789	16	109	911	28
103	125	80/60	66	45	37	90	54	132	25	985	1354	16	130	987	22
104	122	100/60	73	34	37	85	55	121	21	1111	1431	29	111	998	18
105	122	100/60	73	34	37	85	55	145	21	1222	1431	29	158	998	18
106	125	80/60	66	29	37	90	55	579	25	985	1354	21	130	987	22
107	145	90/60	63	40	38	88	55	121	21	999	976	32	87	894	22
108	116	100/60	55	41	37	89	55	183	19	918	999	28	179	911	12
109	125	80/60	66	46	37	90	55	132	25	985	1354	26	130	678	22
110	122	100/60	73	34	37	85	55	498	21	988	1431	21	167	655	18
111	115	90/60	63	40	38	88	55	355	21	1565	1365	27	156	894	27
112	116	100/60	73	41	37	89	55	183	19	1322	1788	28	179	645	12
113	125	80/60	66	45	37	90	79	132	25	985	1354	21	130	987	22
114	122	100/60	55	34	37	85	55	126	21	978	589	29	128	578	18
115	115	90/60	63	40	38	88	55	111	21	1432	1422	32	99	699	22
116	116	100/60	55	41	37	89	55	556	19	1342	1111	21	169	911	28
117	150	90/60	63	38	37	82	69	125	26	987	1011	24	99	578	13
118	125	80/60	66	29	37	90	79	132	25	985	687	26	130	987	22
119	122	100/60	55	34	37	85	55	134	21	999	1431	14	99	788	18
120	110	90/60	63	37	37	85	62	156	34	1111	677	22	134	789	17
121	130	90/60	55	38	38	80	44	147	34	995	998	18	121	915	14
122	117	90/60	63	40	38	82	55	183	24	1233	879	19	130	895	12
123	125	80/60	55	29	37	90	56	550	25	985	1354	22	130	987	22
124	115	90/60	55	38	37	82	69	145	26	1567	1221	22	98	919	13

No Jusvinza	72h															96h
Código	FC	PA	PAM	FR	Temp	SO_2	PO_2	P/F	NLR	INL	Ferritina	PCR	Eritro	Fibrinógeno	DD	IL-6
1	110	90/60	63	41	37	83	60	86	28	1326	987	28	135	988	29	
2	135	90/60	63	35	38	80	60	200	19	998	1124	19	133	988	16	
3	118	90/60	63	38	38	82	60	200	29	1431	2143	21	137	888	21	
4	118	90/60	63	45	38	82	54	77	34	1443	2111	39	198	987	27	603,1
5	119	100/60	73	39	37	89	54	180	22	1478	1888	29	145	987	17	
6	120	90/60	63	40	35	92	56	187	29	1476	1789	31	222	915	19	
7	118	90/60	63	45	38	82	54	77	34	1567	2111	39	198	987	27	
8	125	90/60	63	38	37	82	60	86	29	1249	1325	29	155	943	18	
9	130	100/60	73	34	37	82	52	65	35	1356	1532	31	344	999	29	
10	118	90/60	63	45	38	82	54	77	34	1567	3675	39	198	987	27	
11	136	100/60	73	39	37	89	54	180	22	1675	1888	29	198	987	17	
12	130	80/60	66	39	37	83	62	154	25	1111	1421	29	165	965	19	
13	130	100/60	73	34	37	82	52	65	35	1601	1532	31	176	999	29	
14	138	90/60	63	45	38	82	54	131	27	1567	1209	39	198	987	27	300
15	143	90/60	63	38	37	82	60	86	29	1467	1325	29	155	943	18	
16	130	80/60	66	39	37	83	62	89	28	1111	1421	29	9	965	19	
17	125	90/60	63	38	37	82	60	86	29	1689	1325	29	155	943	18	
18	129	90/60	63	45	38	82	54	77	34	1567	1986	39	188	987	27	
19	129	100/60	73	39	37	89	54	180	22	1465	1888	29	198	987	17	76,3
20	125	90/60	63	38	37	82	60	86	29	1275	1325	29	155	943	18	
21	130	80/60	66	39	37	83	62	111	28	1111	1421	29	187	965	19	
22	130	100/60	73	34	37	82	52	65	35	1322	1532	31	99	999	29	
23	130	80/60	66	39	37	83	62	121	28	1111	1421	29	178	965	19	
24	132	90/60	63	45	38	82	54	77	34	1567	2103	39	198	987	27	
25	119	100/60	73	39	37	89	54	180	22	1566	1888	29	198	987	17	
26	158	90/60	63	38	37	82	60	111	29	1689	1325	29	155	943	18	
27	130	80/60	66	39	37	83	62	89	28	1111	1421	29	221	965	19	
28	130	100/60	73	34	37	82	52	65	35	1601	1532	31	122	999	29	
29	145	90/60	63	45	38	82	54	77	34	1567	2311	39	198	987	27	

30	139	100/60	73	39	37	89	54	180	22	1766	1888	29	198	987	17	
31	125	90/60	63	38	37	82	60	86	29	1689	1325	29	155	943	18	
32	130	80/60	66	39	37	83	62	89	28	1111	1123	29	168	965	19	
33	130	100/60	73	34	37	82	52	142	26	1601	1421	31	165	999	29	
34	135	90/60	63	45	38	82	54	77	34	1567	1987	39	198	987	27	
35	148	90/60	63	38	37	82	60	86	29	1433	1325	29	155	943	18	
36	130	80/60	66	39	37	83	62	132	27	1111	1421	29	149	965	19	
37	118	90/60	63	45	38	82	54	77	34	1567	1945	39	198	987	27	
38	125	90/60	63	38	37	82	60	86	29	1655	1325	29	155	943	18	
39	130	80/60	66	39	37	83	62	89	28	1111	1421	29	221	965	19	
40	156	90/60	63	38	37	82	60	154	25	1365	1325	29	155	943	18	
41	130	80/60	66	39	37	83	62	89	28	1111	1421	29	221	965	19	
42	130	100/60	73	34	37	82	52	65	35	1601	1532	31	222	999	29	
43	130	80/60	66	39	37	83	62	89	28	1111	1421	29	221	965	19	
44	118	90/60	63	45	38	82	54	77	34	1567	1765	39	198	987	27	
45	119	100/60	73	39	37	89	54	180	22	1232	1888	29	198	987	17	
46	125	90/60	63	38	37	82	60	123	22	1689	1325	29	155	943	18	
47	130	80/60	66	39	37	83	62	89	28	1111	1421	29	221	965	19	
48	130	100/60	73	34	37	82	52	65	35	1601	1532	31	189	999	29	
49	118	90/60	63	45	38	82	54	77	34	1567	1678	39	198	987	27	
50	167	100/60	73	39	37	89	54	180	22	1456	1888	29	198	987	17	
51	130	80/60	66	39	37	83	62	89	28	1111	1421	29	221	965	19	
52	130	100/60	73	34	37	82	52	145	27	1601	1532	31	167	999	29	
53	118	90/60	63	45	38	82	54	77	27	1567	1879	39	198	987	27	
54	119	100/60	73	39	37	89	54	180	22	1899	1888	29	198	987	17	
55	130	80/60	66	39	37	83	62	89	28	1111	1421	29	221	965	19	
56	130	100/60	73	34	37	82	52	65	22	1601	1532	31	199	999	29	
57	145	90/60	63	45	38	82	54	77	34	1567	2001	39	198	987	27	
58	139	100/60	73	39	37	89	54	180	22	1899	1888	29	198	987	17	
59	130	80/60	66	39	37	83	62	89	28	1111	1421	29	221	965	19	
60	130	100/60	73	34	37	82	52	111	26	1601	1532	31	344	999	29	
61	165	90/60	63	45	38	82	54	77	34	1567	2111	39	198	987	27	
62	119	100/60	73	39	37	89	54	180	22	1899	1888	29	198	987	17	

63	130	80/60	66	39	37	83	62	89	28	1111	1421	29	221	965	19	
64	130	100/60	73	34	37	82	52	65	35	1601	1532	31	344	999	29	
65	118	90/60	63	45	38	82	54	77	34	1567	2006	39	198	987	27	
66	135	100/60	73	39	37	89	54	180	22	1899	1888	29	198	987	17	
67	130	80/60	66	39	37	83	62	89	28	1111	1421	29	89	965	19	
68	130	100/60	73	34	37	82	52	65	35	1601	1532	31	211	999	29	
69	118	90/60	63	45	38	82	54	77	34	1567	2111	39	198	987	27	
70	129	100/60	73	39	37	89	54	180	22	1899	1888	29	198	987	17	
71	130	80/60	66	39	37	83	62	89	28	1111	1421	29	221	965	19	
72	130	100/60	73	34	37	82	52	121	35	1601	1532	31	232	999	29	
73	118	90/60	63	45	38	82	54	77	34	1567	1896	39	198	987	27	
74	119	100/60	73	39	37	89	54	180	22	1899	1888	29	198	987	17	
75	125	90/60	63	38	37	82	60	86	29	1689	1325	29	155	943	18	
76	130	80/60	66	39	37	83	62	121	25	1111	1421	29	221	965	19	
77	130	100/60	73	34	37	82	52	65	35	1601	1532	31	344	999	29	
78	135	90/60	63	45	38	82	54	77	34	1567	1897	39	198	987	27	
79	129	100/60	73	39	37	89	54	180	22	1899	1888	29	198	987	17	
80	130	80/60	66	39	37	83	62	89	28	1111	1421	29	221	965	19	
81	130	100/60	73	34	37	82	52	111	27	1601	1532	31	111	999	29	
82	129	90/60	63	45	38	82	54	77	34	1567	1234	39	198	987	27	
83	146	100/60	73	39	37	89	54	180	22	1899	1888	29	198	987	17	
84	130	80/60	66	39	37	83	62	89	28	1111	1421	29	221	965	19	
85	130	100/60	73	34	37	82	52	123	35	1601	1532	31	199	999	29	
86	160	90/60	63	38	37	82	60	86	29	1689	1325	29	155	943	18	
87	150	80/60	66	39	37	83	62	89	28	1111	1421	29	221	965	19	38,2
88	130	100/60	73	34	37	82	52	65	35	1601	1532	31	99	999	29	580,2
89	118	90/60	63	45	38	82	54	77	34	1567	897	39	198	987	27	243,7
90	137	100/60	73	39	37	89	54	180	22	1899	1888	29	198	987	17	
91	130	80/60	66	39	37	83	62	89	28	1111	1421	29	221	965	19	
92	130	100/60	73	34	37	82	52	65	35	1601	1532	31	209	999	29	
93	138	90/60	63	45	38	87	54	145	27	1567	1897	39	198	987	27	
94	136	100/60	73	39	37	89	54	180	22	1899	1888	29	198	987	17	
95	130	80/60	66	39	37	83	62	89	28	1111	1421	29	221	965	19	

96	130	100/60	73	34	37	82	52	65	35	1601	1532	31	199	999	29	
97	227	90/60	63	45	38	82	54	77	34	1567	1789	39	198	987	27	
98	142	100/60	73	39	37	89	54	180	22	1899	1888	29	198	987	17	
99	189	80/60	66	39	37	87	62	89	28	1111	1421	29	221	965	19	
100	130	100/60	73	34	37	82	52	459	28	1601	1532	31	209	999	29	
101	133	90/60	63	45	38	82	54	77	34	1567	1999	39	198	987	27	90,3
102	139	100/60	55	39	37	89	54	890	22	1899	1888	29	198	987	17	
103	130	80/60	66	39	37	88	62	567	25	1111	1421	29	221	965	19	
104	130	100/60	55	34	37	82	52	65	29	1601	1532	31	79	999	29	
105	130	100/60	73	34	37	82	52	65	28	1601	1532	31	122	999	29	
106	166	80/60	66	39	37	99	62	689	28	1111	1421	29	99	965	19	51.4
107	158	90/60	63	45	38	90	54	77	27	1567	1987	39	198	987	27	
108	134	100/60	55	39	37	89	54	180	22	1899	1888	29	198	987	17	
109	130	80/60	66	39	37	83	62	89	28	1111	1421	29	167	965	19	
110	145	100/60	73	34	37	88	52	156	27	1601	1532	31	199	999	29	
111	139	90/60	55	45	38	88	54	166	22	1567	2111	39	198	987	27	
112	165	100/60	73	39	37	89	54	180	22	1378	1888	29	89	987	17	
113	145	80/60	55	39	37	99	62	355	28	1200	1421	29	177	965	19	
114	130	100/60	55	34	37	88	52	65	29	1601	1237	31	98	678	29	
115	145	90/60	55	45	38	82	54	77	27	1567	2009	39	198	987	27	
116	189	100/60	73	39	37	89	54	180	22	1222	1888	29	89	987	17	
117	176	90/60	63	38	37	82	60	86	29	1689	1325	29	78	589	18	
118	145	80/60	66	39	37	83	62	89	28	2333	1421	29	99	655	19	
119	130	100/60	73	34	37	82	52	65	29	1601	1231	31	178	999	29	
120	137	90/60	55	41	37	99	60	111	27	1326	1111	28	135	689	29	
121	135	90/60	63	35	38	99	60	200	19	1765	1124	19	88	677	16	
122	145	90/60	55	38	38	82	60	556	29	1543	1988	21	137	588	21	
123	155	80/60	55	39	37	99	62	457	26	2356	1421	29	87	378	19	
124	145	90/60	45	38	37	88	60	98	29	1689	1325	29	155	943	18	

No Jusvinza	7d														
Código	FC	PA	PAM	FR	Temp	SO_2	PO_2	P/F	INL	LDH	Ferritina	PCR	Eritro	Fibrinógeno	DD
1	130	80/60	66	39	37	83	62	89	28	1111	1421	29	9	965	19
2	129	100/60	73	39	37	89	54	180	22	1465	1888	29	198	987	17
3	130	80/60	66	39	37	83	62	111	28	1111	1421	29	187	965	19
4	125	90/60	63	38	37	82	60	86	29	1689	1325	29	155	943	18
5	129	90/60	63	45	38	82	54	77	34	1567	1986	39	188	987	27
6	138	90/60	63	45	38	82	54	131	27	1567	1209	39	198	987	27
7	132	90/60	63	45	38	82	54	77	34	1567	2103	39	198	987	27
8	119	100/60	73	39	37	89	54	180	22	1566	1888	29	198	987	17
9	130	80/60	66	39	37	83	62	89	28	1111	1421	29	221	965	19
10	167	100/60	73	39	37	89	54	180	22	1456	1888	29	198	987	17
11	130	80/60	66	39	37	83	62	121	28	1111	1421	29	178	965	19
12	158	90/60	63	38	37	82	60	111	29	1689	1325	29	155	943	18
13	125	90/60	63	38	37	82	60	86	29	1275	1325	29	155	943	18
14	120	90/60	63	40	35	92	56	187	29	1476	1789	31	222	915	19
15	130	100/60	73	34	37	82	52	65	35	1322	1532	31	99	999	29
16	139	100/60	73	39	37	89	54	180	22	1766	1888	29	198	987	17
17	118	90/60	63	45	38	82	54	77	34	1443	2111	39	198	987	27
18	119	100/60	73	39	37	89	54	180	22	1478	1888	29	145	987	17
19	130	100/60	73	34	37	82	52	142	26	1601	1421	31	165	999	29
20	130	100/60	73	34	37	82	52	65	35	1601	1532	31	176	999	29
21	125	90/60	63	38	37	82	60	123	22	1689	1325	29	155	943	18
22	143	90/60	63	38	37	82	60	86	29	1467	1325	29	155	943	18
23	130	80/60	66	39	37	83	62	132	27	1111	1421	29	149	965	19
24	130	80/60	66	39	37	83	62	89	28	1111	1421	29	221	965	19
25	130	100/60	73	34	37	82	52	65	35	1601	1532	31	122	999	29
26	156	90/60	63	38	37	82	60	154	25	1365	1325	29	155	943	18
27	118	90/60	63	45	38	82	54	77	34	1567	2111	39	198	987	27
28	125	90/60	63	38	37	82	60	86	29	1249	1325	29	155	943	18
29	130	100/60	73	34	37	82	52	65	35	1356	1532	31	344	999	29
30	130	80/60	66	39	37	83	62	89	28	1111	1421	29	221	965	19

31	145	90/60	63	45	38	82	54	77	34	1567	2311	39	198	987	27
32	118	90/60	63	45	38	82	54	77	34	1567	1945	39	198	987	27
33	130	80/60	66	39	37	83	62	89	28	1111	1421	29	221	965	19
34	125	90/60	63	38	37	82	60	86	29	1689	1325	29	155	943	18
35	130	80/60	66	39	37	83	62	89	28	1111	1123	29	168	965	19
36	135	100/60	73	39	37	89	54	180	22	1899	1888	29	198	987	17
37	130	80/60	66	39	37	83	62	89	28	1111	1421	29	221	965	19
38	130	100/60	73	34	37	82	52	65	35	1601	1532	31	189	999	29
39	118	90/60	63	45	38	82	54	77	34	1567	1678	39	198	987	27
40	118	90/60	63	45	38	82	54	77	27	1567	1879	39	198	987	27
41	118	90/60	63	45	38	82	54	77	34	1567	1765	39	198	987	27
42	119	100/60	73	39	37	89	54	180	22	1232	1888	29	198	987	17
43	130	100/60	73	34	37	82	52	65	22	1601	1532	31	199	999	29
44	135	90/60	63	45	38	82	54	77	34	1567	1987	39	198	987	27
45	148	90/60	63	38	37	82	60	86	29	1433	1325	29	155	943	18
46	130	100/60	73	34	37	82	52	145	27	1601	1532	31	167	999	29
47	125	90/60	63	38	37	82	60	86	29	1655	1325	29	155	943	18
48	130	80/60	66	39	37	83	62	89	28	1111	1421	29	221	965	19
49	130	80/60	66	39	37	83	62	89	28	1111	1421	29	221	965	19
50	130	100/60	73	34	37	82	52	111	26	1601	1532	31	344	999	29
51	130	100/60	73	34	37	82	52	65	35	1601	1532	31	222	999	29
52	136	100/60	73	39	37	89	54	180	22	1675	1888	29	198	987	17
53	130	80/60	66	39	37	83	62	154	25	1111	1421	29	165	965	19
54	145	90/60	63	45	38	82	54	77	34	1567	2001	39	198	987	27
55	139	100/60	73	39	37	89	54	180	22	1899	1888	29	198	987	17
56	129	100/60	73	39	37	89	54	180	22	1899	1888	29	198	987	17
57	119	100/60	73	39	37	89	54	180	22	1899	1888	29	198	987	
58	130	80/60	66	39	37	83	62	89	28	1111	1421	29	221	965	
59	165	90/60	63	45	38	82	54	77	34	1567	2111	39	198	987	27
60	130	100/60	73	34	37	82	52	121	35	1601	1532	31	232	999	29
61	130	80/60	66	39	37	83	62	89	28	1111	1421	29	221	965	19
62	118	90/60	63	45	38	82	54	77	34	1567	2111	39	198	987	27
63	130	80/60	66	39	37	83	62	121	25	1111	1421	29	221	965	19

64	130	80/60	66	39	37	83	62	89	28	1111	1421	29	89	965	19
65	130	100/60	73	34	37	82	52	65	35	1601	1532	31	211	999	29
66	130	80/60	66	39	37	83	62	89	28	1111	1421	29	221	965	19
67	119	100/60	73	39	37	89	54	180	22	1899	1888	29	198	987	17
68	130	100/60	73	34	37	82	52	65	35	1601	1532	31	344	999	29
69	118	90/60	63	45	38	82	54	77	34	1567	2006	39	198	987	27
70	130	100/60	73	34	37	82	52	111	27	1601	1532	31	111	999	29
71	118	90/60	63	45	38	82	54	77	34	1567	1896	39	198	987	27
72	146	100/60	73	39	37	89	54	180	22	1899	1888	29	198	987	17
73	130	80/60	66	39	37	83	62	89	28	1111	1421	29	221	965	19
74	135	90/60	63	45	38	82	54	77	34	1567	1897	39	198	987	
75	129	100/60	73	39	37	89	54	180	22	1899	1888	29	198	987	
76	130	100/60	73	34	37	82	52	123	35	1601	1532	31	199	999	29
77	119	100/60	73	39	37	89	54	180	22	1899	1888	29	198	987	17
78	125	90/60	63	38	37	82	60	86	29	1689	1325	29	155	943	18
79	130	100/60	73	34	37	82	52	65	35	1601	1532	31	344	999	29
80	160	90/60	63	38	37	82	60	86	29	1689	1325	29	155	943	18
81	137	100/60	73	39	37	89	54	180	22	1899	1888	29	198	987	17
82	118	90/60	63	45	38	82	54	77	34	1567	897	39	198	987	27
83	138	90/60	63	45	38	87	54	145	27	1567	1897	39	198	987	27
84	129	90/60	63	45	38	82	54	77	34	1567	1234	39	198	987	27
85	130	100/60	73	34	37	82	52	65	35	1601	1532	31	199	999	29
86	130	100/60	73	34	37	82	52	459	28	1601	1532	31	209	999	29
87	136	100/60	73	39	37	89	54	180	22	1899	1888	29	198	987	17
88	130	80/60	66	39	37	83	62	89	28	1111	1421	29	221	965	19
89	130	80/60	66	39	37	83	62	89	28	1111	1421	29	221	965	19
90	139	100/60	55	39	37	89	54	890	22	1899	1888	29	198	987	17
91	150	80/60	66	39	37	83	62	89	28	1111	1421	29	221	965	19
92	130	100/60	73	34	37	82	52	65	35	1601	1532	31	99	999	29
93	130	80/60	66	39	37	88	62	567	25	1111	1421	29	221	965	19
94	130	80/60	66	39	37	83	62	89	28	1111	1421	29	221	965	19
95	130	100/60	73	34	37	82	52	65	35	1601	1532	31	209	999	29
96	166	80/60	66	39	37	99	62	689	28	1111	1421	29	99	965	19

97	158	90/60	63	45	38	90	54	77	27	1567	1987	39	198	987	27
98	134	100/60	55	39	37	89	54	180	22	1899	1888	29	198	987	17
99	130	80/60	66	39	37	83	62	89	28	1111	1421	29	167	965	19
100	145	80/60	55	39	37	99	62	355	28	1200	1421	29	177	965	19
101	165	100/60	73	39	37	89	54	180	22	1378	1888	29	89	987	17
102	145	100/60	73	34	37	88	52	156	27	1601	1532	31	199	999	29
103	139	90/60	55	45	38	88	54	166	22	1567	2111	39	198	987	27
104	130	100/60	55	34	37	88	52	65	29	1601	1237	31	98	678	29
105	145	90/60	55	45	38	82	54	77	27	1567	2009	39	198	987	27
106	189	100/60	73	39	37	89	54	180	22	1222	1888	29	89	987	17
107	227	90/60	63	45	38	82	54	77	34	1567	1789	39	198	987	27
108	142	100/60	73	39	37	89	54	180	22	1899	1888	29	198	987	17
109	189	80/60	66	39	37	87	62	89	28	1111	1421	29	221	965	19
110	137	90/60	55	41	37	99	60	111	27	1326	1111	28	135	689	29
111	135	90/60	63	35	38	99	60	200	19	1765	1124	19	88	677	16
112	133	90/60	63	45	38	82	54	77	34	1567	1999	39	198	987	27
113	145	90/60	55	38	38	82	60	556	29	1543	1988	21	137	588	21
114	130	100/60	55	34	37	82	52	65	29	1601	1532	31	79	999	29
115	130	100/60	73	34	37	82	52	65	28	1601	1532	31	122	999	29
116	155	80/60	55	39	37	99	62	457	26	2356	1421	29	87	378	19
117	176	90/60	63	38	37	82	60	86	29	1689	1325	29	78	589	18
118	145	80/60	66	39	37	83	62	89	28	2333	1421	29	99	655	19
119	130	100/60	73	34	37	82	52	65	29	1601	1231	31	178	999	29
120	110	90/60	63	41	37	83	60	85,71	28	1326	987	28	135	988	19
121	135	90/60	63	35	38	80	60	200	19	998	1124	19	133	988	17
122	118	90/60	63	38	38	82	60	200	29	1431	2143	21	137	888	19
123	155	80/60	55	39	37	99	62	457	26	2356	1421	29	87	378	19
124	165	100/60	73	39	37	89	54	180	22	1378	1888	29	89	987	17
